# Design, Synthesis, and Antitumor Activities of Novel Coumarin-Based Histone Deacetylase Inhibitors

**DOI:** 10.3390/biom16070978

**Published:** 2026-07-03

**Authors:** Sichang Yan, Jie Chang, Dongyu Lei, Xiangyang Lv, Yanzhuo Li, Yue Zhuo, Lu Jin, Le Pan

**Affiliations:** 1 College of Chemistry and Chemical Engineering, Xinjiang Agricultural University, Urumqi 830052, China; 2 Department of Physiology, Preclinical School, Xinjiang Medical University, Urumqi 830011, China

**Keywords:** coumarin derivatives, design and synthesis, HDAC inhibitors, anti-tumor activity, transcriptome sequencing

## Abstract

Histone deacetylases (HDACs) are important epigenetic regulatory enzymes contributing to cancer proliferation, which could be critical targets in cancer therapy. The structural similarities of the existing HDAC inhibitors have resulted in an increase in the drug resistance. In this study, coumarin was employed as the core scaffold for structural derivatisation to develop a novel class of HDAC inhibitors based on computer-aided design (CADD). Their anti-tumor activity was evaluated against esophageal squamous cell lines. The results showed that most compounds exhibited potent anti-proliferative activity against KYSE70 and KYSE150. Among them, compound **4s** and **4p** exhibited the most potent activity with IC_50_ values of 3.44 μM and 3.39 μM against KYSE70. To validate the target of the synthesized compounds, transcriptome sequencing was performed and the results revealed that a total of 487 genes were differentially expressed, including 190 up-regulated and 297 down-regulated genes. Among these, 79 genes were associated with the HDAC regulatory network, accounting for 16.2% of the differentially expressed genes. Molecular docking demonstrated that compound **4s** could effectively enter the active site of HDAC, engaging with the cap group, zinc-binding group, and linker region. This multiple interaction network provides a structural basis for the potent inhibitory activity of compound **4s**. In conclusion, a series of novel HDAC inhibitors with a coumarin scaffold were discovered, and their mode of action was revealed. This provides a valuable guide for the development of novel HDAC-targeting therapeutics.

## 1. Introduction

Cancer is still the leading cause of mortality worldwide [1]. In recent years, significant progress has been made in the development of targeted chemotherapeutics, which have improved survival outcomes for patients with various malignancies, such as epidermal growth factor receptor (EGFR) inhibitors [2,3] and immune checkpoint inhibitors [4] have demonstrated clinical efficacy in cancer trials [5]. However, clinically approved targeted agents remain limited, and the global rise of resistance has led to the increasing undermining of long-term efficacy of target agents by both intrinsic and acquired resistance mechanisms [6]. The scarcity of effective targeted therapies contributes to the poor prognosis, with fewer than 20% of patients surviving beyond five years post-diagnosis [7]. Therefore, there is an urgent need to develop novel targeted therapeutic agents for cancer treatment.

**Figure 1:**
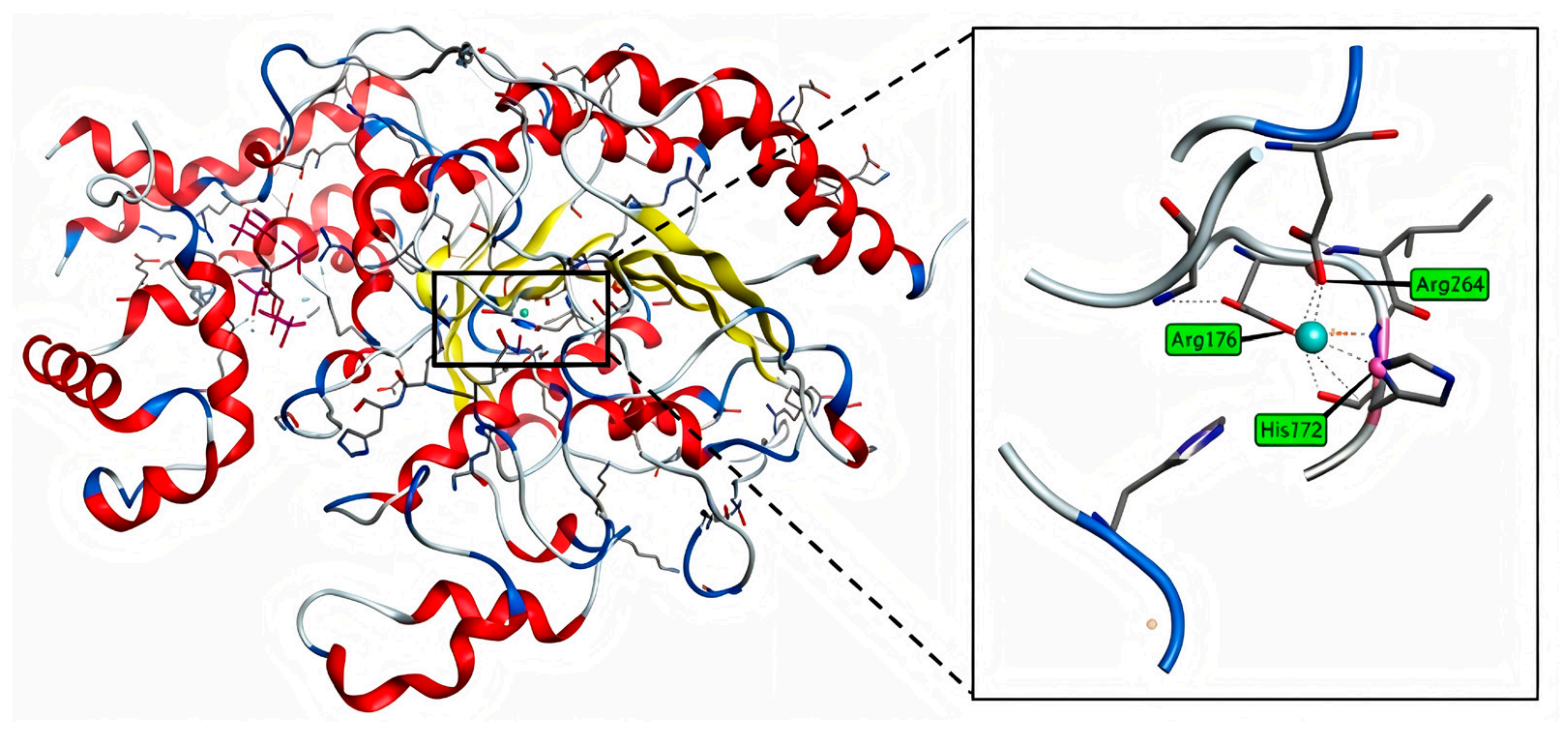
The catalytic domain of HDAC protein.

Histone deacetylases (HDACs) are important epigenetic regulatory enzymes that catalyze the removal of acetyl groups from histones, thereby influencing chromatin structure and gene expression [8]. Dysregulation of HDACs has been implicated in various diseases, including cancer [9], neurological disorders [10], and inflammatory conditions [11]. HDACs are classified into four classes. Class I comprises HDAC1, 2, 3, and 8, which localize to the nucleus and are primarily involved in basal transcriptional repression and cell cycle regulation [12]. Class II consists of HDAC4, 5, 6, 7, 9, and 10, which shuttle between the cytoplasm and nucleus and participate mainly in signal-dependent gene regulation and deacetylation of non-histone proteins [13]. Class III comprises NAD+-dependent deacetylases, including SIRT1-7, which function as energy and stress sensors and play central roles in metabolism, ageing, and maintenance of genomic stability [14]. Class IV contains only HDAC11, which has distinct structural and functional features compared to other HDAC classes and is primarily involved in immune regulation and cellular metabolism. Among these, Class I, II, and IV HDACs are Zn^2+^-dependent. The Zn^2+^-dependent catalytic domains and pharmacophore models of these three classes primarily comprise four binding regions: The Zn^2+^-dependent catalytic domains of Class of I, II, and IV HDACs share a common pharmacophore model consisting of four key binding regions (Figure 1). Inhibition is achieved when the cap group of the inhibitor interacts with the surface binding domain, while the linker group occupies the hydrophobic channel, stabilising the binding. Ultimately the zinc-binding group (ZBG) coordinates with the catalytic zinc ion in the active site [15], thereby blocking hydrolase activity. This interaction between the ZBG and the zinc ion is the structural basis for potent inhibitory activity. The key catalytic domain of the HDAC protein. Additionally, functional groups targeting internal cavities adjacent to the ZBG are often incorporated into inhibitor design to enhance isoform selectivity and inhibitory potency [16]. Therefore, histone deacetylases could be a promising target for anti-cancer agent development.

**Figure 2:**
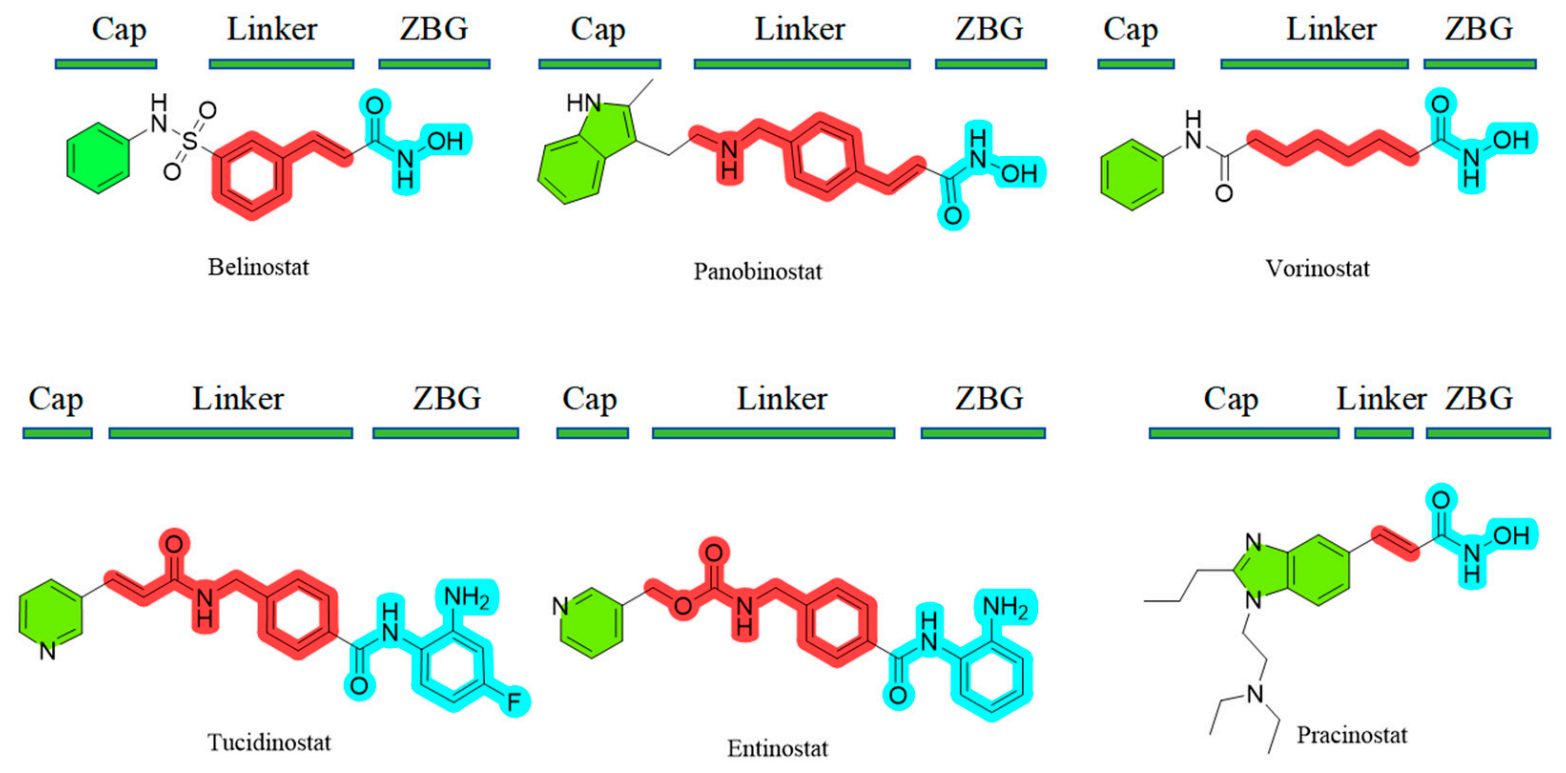
HDAC inhibitor drugs approved for marketing by the FDA up to 2025.

To date, a variety of selective and multi-targeted HDAC inhibitors have been developed, some of which have been approved by the U.S. Food and Drug Administration (FDA) for cancer treatment [17], including Belinostat [18], Vorinostat [19], and Panobinostat [20] (Figure 2). However, most HDAC inhibitors exhibit a high degree of structural similarity, a characteristic that often contributes to the development of drug resistance. To address this issue, discovering novel HDAC inhibitors with new skeletons has become very urgent.

**Figure 3:**
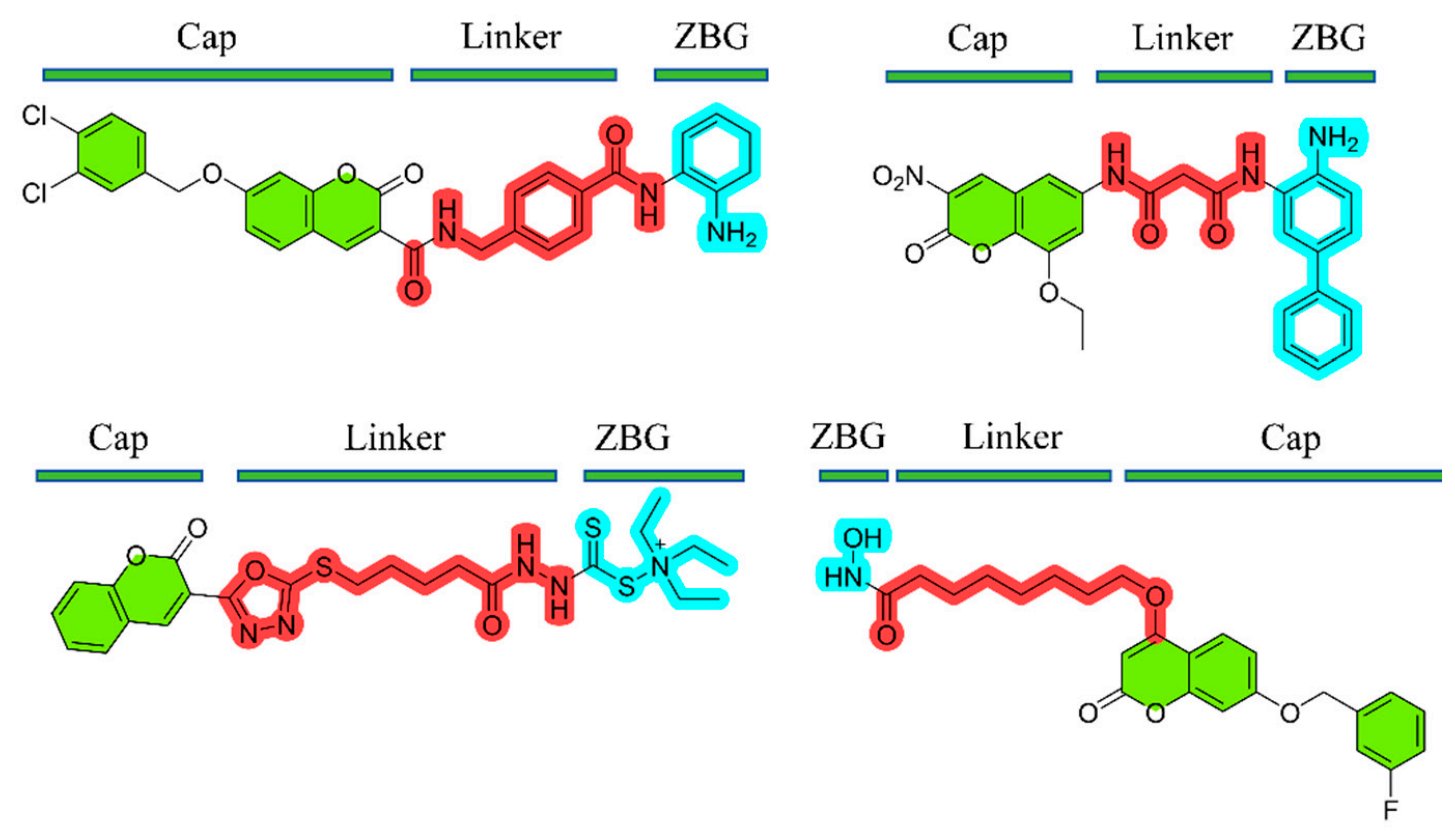
Previously reported coumarin-based HDAC inhibitors.

Coumarins are an important class of naturally abundant bioactive heterocyclic compounds [21] with excellent biological activity and biocompatibility. Both natural or synthetic coumarins and their derivatives exhibit diverse biological activities, including anticancer [22], antifungal [23], antiviral [24], and antioxidant effects [25]. In recent years, the synthesis of coumarins and their derivatives has garnered considerable attention in the development of anticancer therapeutics [26,27,28]. Herein, coumarin was proposed as the core scaffold to design and synthesize novel HDAC inhibitors by connecting the cap group, ZBG, and linker based on computer-aided drug design (CADD). In this study, commercially available HDAC inhibitors were employed as lead compounds for structural design [29,30]. Coumarin, featuring a rigid planar framework, was selected as the core scaffold and a potential bidentate zinc-binding mode was established via the coumarin lactone carbonyl and the benzyl hydroxyl group. This strategy integrates hydrophobic interactions, hydrogen bonding networks, and steric hindrance modulation to develop novel HDAC inhibitors with synergistic effects. In contrast to previously reported coumarin-based HDAC inhibitors (Figure 3) [31], which, like the traditional HDAC inhibitor SAHA, all feature a linear structure, our compound is built around an sp^3^-hybridized nitrogen atom, to which the cap, linker, and ZBG are linked, resulting in an overall trigonal pyramidal structure.

## 2. Materials and Methods

### 2.1. Chemistry

General Methods. All reagents and solvents were obtained from commercial suppliers and used without further purification. ^1^H and ^13^C NMR spectra were recorded on a Bruker 500 MHz spectrometer Technology Co., Ltd., Beijing, China) with tetramethylsilane (TMS) as the internal standard, and samples were prepared in DMSO-d_6_ as the solvent. Spin multiplicities are described as s (singlet), d (doublet), t (triplet), q (quartet), and m (multiplet); coupling constants (J) are reported in hertz (Hz). All reactions were carried out under anhydrous conditions and where necessary, under a nitrogen atmosphere. All solvents were removed under reduced pressure using a rotary evaporator at 45 °C. Melting points were determined on an SGWX-4 melting point apparatus (Shanghai Instrument Physical Optics Instrument Co., Ltd.) and are uncorrected. Compound names given in the experimental section were generated using ChemDraw 22.0.0.

#### 2.1.1. Synthesis of Coumarin Schiff Bases (**1a**–**1c** and **2a**–**2c**)

3-aminocoumarin (1.0 equiv) and 4-fluoro-2-hydroxybenzaldehyde (1.2 equiv) were dissolved in anhydrous methanol. A catalytic amount of glacial acetic acid (three drops) was added to the mixture. The mixture was stirred at 80 °C for 4 h. After completion of the reaction, the solvent was concentrated under reduced pressure using a rotary evaporator. and the product was purified by recrystallization from Methanol to afford 3-((2-hydroxy-4-fluorophenyl)imino)coumarin (**1a**) as a yellow solid.

#### 2.1.2. Synthesis of Reduced Coumarin Schiff Base Derivatives (**3a**–**3f**)

Compound 1a (1.0 equiv) was dissolved in anhydrous dichloromethane. Sodium borohydride (2.0 equiv) was added, and the mixture was stirred at 25 °C for 6 h. After completion of the reaction, the solvent was removed under reduced pressure using a rotary evaporator. The residue was purified by column chromatography on silica gel with dichloromethane/methanol (40:1, *v*/*v*), to afford the product 3-((2-hydroxy-4-fluorophenyl)amino)coumarin (**3a**) as a dark yellow solid.

#### 2.1.3. Synthesis of Coumarin-Benzylamine Hybrid Molecules (**4a**–**4t**)

Compound 3a (1.0 equiv), N-(3-dimethylaminopropyl)-N’-ethylcarbodiimide hydrochloride (EDCI) (3.0 equiv), and 4-(dimethylamino)pyridine (DMAP) (0.2 equiv) were dissolved in anhydrous dichloromethane. The mixture was stirred at 0 °C for 30 min, then allowed to warm to room temperature with continued stirring. 2-methylisobutyric acid(1.3 equiv) was then added dropwise to the mixture using a constant pressure dropping funnel. After completion of the reaction, the solvent was removed under reduced pressure using a rotary evaporator. The residue was purified by column chromatography on silica gel with dichloromethane/ethyl acetate (90:1, *v*/*v*), to afford the product 3-(N-(2-hydroxy-4-fluorobenzyl)-N-isobutyrylamino)coumarin (**4a**) as a yellow solid.

### 2.2. Pharmacology

#### 2.2.1. Preliminary In Vitro Anticancer Activity Screening

Stock solutions of all compounds were prepared at concentrations of 40 μg/mL and 10 μg/mL. Cells were seeded into 96-well microplates at a density of 4 × 10^4^ cells per well and incubated for 24 h. The cells were then treated with the prepared compound solutions for 24 h and 48 h. Subsequently, 20 μL of MTT solution (500 μg/mL) was added to each well, and the plates were incubated at 37 °C for 5 h. After incubation, the supernatant was carefully removed, and 150 μL of DMSO was added to each well to dissolve the blue-purple formazan crystals. Finally, the absorbance was measured at 490 nm using a microplate reader (Thermo Fisher Scientific, Waltham, MA, USA). The experiment was performed in triplicate. Untreated cells served as the negative compound group, and wells containing no cells served as the blank group. The inhibition rates of the compounds against cancer cells were calculated based on the recorded results.

#### 2.2.2. Half-Maximal Inhibitory Concentration (IC_50_) Determination

Based on the preliminary screening results, compounds exhibiting inhibition rates greater than 80% at a concentration of 10 μg/mL were selected for half-maximal inhibitory concentration (IC_50_) determination. The inhibition rates of all selected compounds were measured at concentrations of 10, 8, 6, 4, 2, 1, and 0.5 μg/mL. Cancer cells treated without any compound served as the negative control group, and wells without any cancer cells served as the blank group. All experiments were performed in triplicate. The IC_50_ values of the compounds against cancer cells were calculated based on concentration-dependent curves using SPSS 27.0.

#### 2.2.3. Annexin V/PI Apoptosis Assay

Apoptosis was analyzed using the Annexin V-FITC/PI Apoptosis Detection Kit. KYSE70 cells were seeded into six-well plates and treated with various concentrations of compound **4s** (0, 10, 20, and 40 μg/mL) for 48 h. Cells were harvested by trypsinization, washed twice with pre-cooled PBS, and resuspended in 1× Annexin V binding buffer. The cells were stained with Annexin V-FITC and PI for 15 min at room temperature in the dark. Flow cytometric analysis (Hangzhou POWCLIN Medical Technology Co., Ltd., Hangzhou, China) was performed within 1 h after staining. Quantitative analysis was performed, and data were analyzed using FlowJo software (version 10.8.1) [31].

#### 2.2.4. Reference-Guided Eukaryotic Transcriptome Sequencing

##### RNA Extraction and Library Construction

Total RNA was extracted using TRIzol reagent according to the manufacturer’s instructions. RNA purity and concentration were determined with a NanoDrop 2000 spectrophotometer (Thermo Scientific, USA), and RNA integrity was assessed with an Agilent 2100 Bioanalyzer (Agilent Technologies, Santa Clara, CA, USA). Transcriptome libraries were constructed using the VAHTS Universal V10 RNA-seq Library Prep Kit (Premixed Version) following the manufacturer’s protocol. Transcriptome sequencing and data analysis were performed by Shanghai OE Biotech Co., Ltd. (Shanghai, China).

##### RNA Sequencing and Differential Expression Gene Analysis

Libraries were sequenced on the Illumina NovaSeq 6000 platform to generate 150 bp paired-end reads. Approximately 24.55 million raw reads were obtained per sample. Raw reads in FASTQ format were processed using fastp softw (version 0.20.1) are [32] to remove low-quality reads, yielding clean reads for subsequent data analysis. Reference genome alignment was performed using HISAT2 software (version 2.1.0) [33], and gene expression levels were calculated as fragments per kilobase of transcript per million mapped reads (FPKM) [34]. Read counts for each gene were obtained using HTSeq-count [35]. Principal component analysis (PCA) of gene counts was performed and visualized using R (version 3.2.0) to assess biological replicates among samples.

Differential gene expression analysis was conducted using DESeq2 software [36], with genes meeting the thresholds of q-value < 0.05 and fold change > 2 or fold change < 0.5 defined as differentially expressed genes (DEGs). Hierarchical clustering analysis of DEGs was performed using R (version 3.2.0) to visualize expression patterns across different groups and samples. The R package ggradar was used to generate radar plots of the top 30 genes, illustrating expression changes of up-regulated and down-regulated genes.

Subsequently, enrichment analyses of GO [37], KEGG pathway [38], Reactome, and WikiPathways were performed for DEGs based on the hypergeometric distribution algorithm to identify significantly enriched functional terms. Bar plots, chord diagrams, and enrichment analysis circle plots for significantly enriched functional terms were generated using R (version 3.2.0).

Gene set enrichment analysis (GSEA) was performed using the GSEA softw (version 2.1.0) are [39,40]. Genes were ranked according to their expression differences between the two sample groups. Enrichment was then examined at the top or bottom of this ranked list using predefined gene sets.

#### 2.2.5. In Vitro HDAC Enzymatic Activity Assay

The BBcellProbe^®^ HDAC Activity Assay Kit (fluorescent method, BestBio, BB-491462) was used to detect HDAC enzyme activity in live cells in situ. This kit uses an acetylated fluorescent substrate capable of penetrating the cell membrane, with a fluorescent group and a quencher group attached to each end, which suppress fluorescence via the FRET effect. After the substrate enters the cell, it is deacetylated by active HDAC enzymes. Subsequently, a developer is added, and proteases specifically cleave the deacetylated substrate, releasing free fluorophores. The fluorescence intensity is directly proportional to HDAC enzyme activity.

The experiment was conducted in a black 96-well plate designed for fluorescence detection. At least 4 × 10^4^ cells were seeded into each well. After treatment with different concentrations of compounds **4s** and **4p** for a specified duration, 50 μL of the detection buffer working solution containing the HDAC fluorescent substrate (detection buffer: substrate = 50:1) was added to each well, and the plates were incubated at 37 °C in the dark for 3 h. Subsequently, 50 μL of developer solution is added to each well, and the plates are incubated at 37 °C in the dark for 3 h. Fluorescence intensity was measured using a fluorescence microplate reader (Thermo Fisher Scientific, Waltham, Massachusetts) at an excitation wavelength of 360 nm and an emission wavelength of 460 nm. The IC_50_ was determined by a four-parameter logistic (4PL) model.

#### 2.2.6. Molecular Docking Studies

Molecular docking studies were performed using MOE 2019.0102 to investigate the potential binding modes between the target compounds and histone deacetylase (HDAC). The target compounds were drawn using ChemDraw 22.0. The crystal structure of HDAC1 (PDB ID: 5ICN) was obtained from the RCSB Protein Data Bank (PDB, https://www.rcsb.org/), at a resolution of 3.30 Å. Prior to docking simulations, the receptor protein was pre-processed by removing surrounding water molecules, adding hydrogen atoms, and performing energy minimization. During docking simulations, the receptor was kept rigid while the ligands were treated as flexible. The optimal binding conformations were selected based on the docking results to analyze the binding interactions at the active site.

#### 2.2.7. Molecular Dynamics Simulation Studies

##### System Setup

The initial three-dimensional structure of human histone deacetylase 1 (HDAC1) was obtained from the RCSB Protein Data Bank (PDB ID: 5ICN). Missing residues in the structure were repaired using pdbfixer (version 1.12.0) [41]. The protein was modeled with the ff14SB force field using the lipid17 correction parameters [42]. The ligand was parameterized using Sobtop [43] based on the GAFF force field. The protein–ligand complex was placed in a cubic box and solvated using the TIP3P water model, with a minimum distance of 1.0 nm from the protein surface to the box boundary. Na^+^ and Cl^−^ ions were added to neutralize the system and achieve a physiological saline concentration.

##### Energy Minimization and System Equilibration

Molecular dynamics simulations of the protein–ligand complex were performed using the GROMACS (version 2025.4) [44]. First, energy minimization was carried out using the steepest descent method. Subsequently, a 2 ns constant number of particles, volume, and temperature (NVT) equilibration was performed at 298 K, during which positional restraints were applied to the atoms of the protein–ligand complex using the V-rescale thermostat. NPT equilibration was then conducted under the same positional restraints using the V-rescale thermostat and the Parrinello–Rahman barostat.

##### Production Simulation

A 100 ns NPT production simulation (298 K, 1 atm) was performed without any restraints, with a time step of 2 fs. The temperature and pressure coupling methods were the same as those used in the NPT equilibration. All bonds involving hydrogen atoms were constrained using the LINCS algorithm. Long-range electrostatic interactions were treated using the Particle Mesh Ewald (PME) method with a real-space cutoff of 1.2 nm and an FFT grid spacing of 0.16 nm. Van der Waals interactions were truncated at 1.2 nm, with a force-switch smoothing function applied between 1.0 and 1.2 nm. Coordinates were saved every 10 ps.

##### Trajectory Analysis

Root mean square deviation (RMSD) and root mean square fluctuation (RMSF) were calculated using **gmx rms** and **gmx rmsf**, respectively. Prior to calculation, the trajectories were least-squares fitted to the initial structure. Hydrogen bonds: Hydrogen bonds between the protein and the ligand were analyzed using the **gmx hbond** command with default geometric criteria (donor–acceptor distance ≤ 0.35 nm and angle ≤ 30°). Secondary structure was assigned using the DSSP algorithm via **gmx dssp** command [45]. Binding free energy: Binding free energies were calculated using the MM/PBSA method as implemented in the **gmx_MMPBSA** tool [46]. 21 frames were extracted uniformly from the last 80 ns of the simulation (80–100 ns). The total binding free energy was decomposed into van der Waals energy, electrostatic, polar solvation, and nonpolar solvation energy components.

#### 2.2.8. 3D-QSAR Model Establishment

The 3D-QSAR model was constructed using SYBYL-X 2.1.1 software. All compound structures were drawn using ChemDraw 22.0 and imported into the software. Energy minimization of all compounds was performed using the Tripos force field and Gasteiger-Hückel charges. Compound **4s**, the most potent compound, was selected as the template molecule, and the coumarin scaffold was used as the common substructure for molecular alignment. Among the 20 final target compounds, 15 were selected as the training set. The CoMFA model was constructed using partial least squares (PLS) analysis. Cross-validation was performed using the leave-one-out method to calculate the optimal cross-validated correlation coefficient (q^2^). Subsequently, the optimal number of components (NOC) was selected for non-cross-validated analysis to obtain the non-cross-validated correlation coefficient (r^2^), standard error of estimate (SEE), and Fisher’s F value. These statistical parameters were used to evaluate the reliability of the constructed CoMFA model. The remaining five compounds were selected as the test set to further assess the predictive ability of the established model.

## 3. Results

### 3.1. The Synthesis of HDACs

**Scheme 1:**
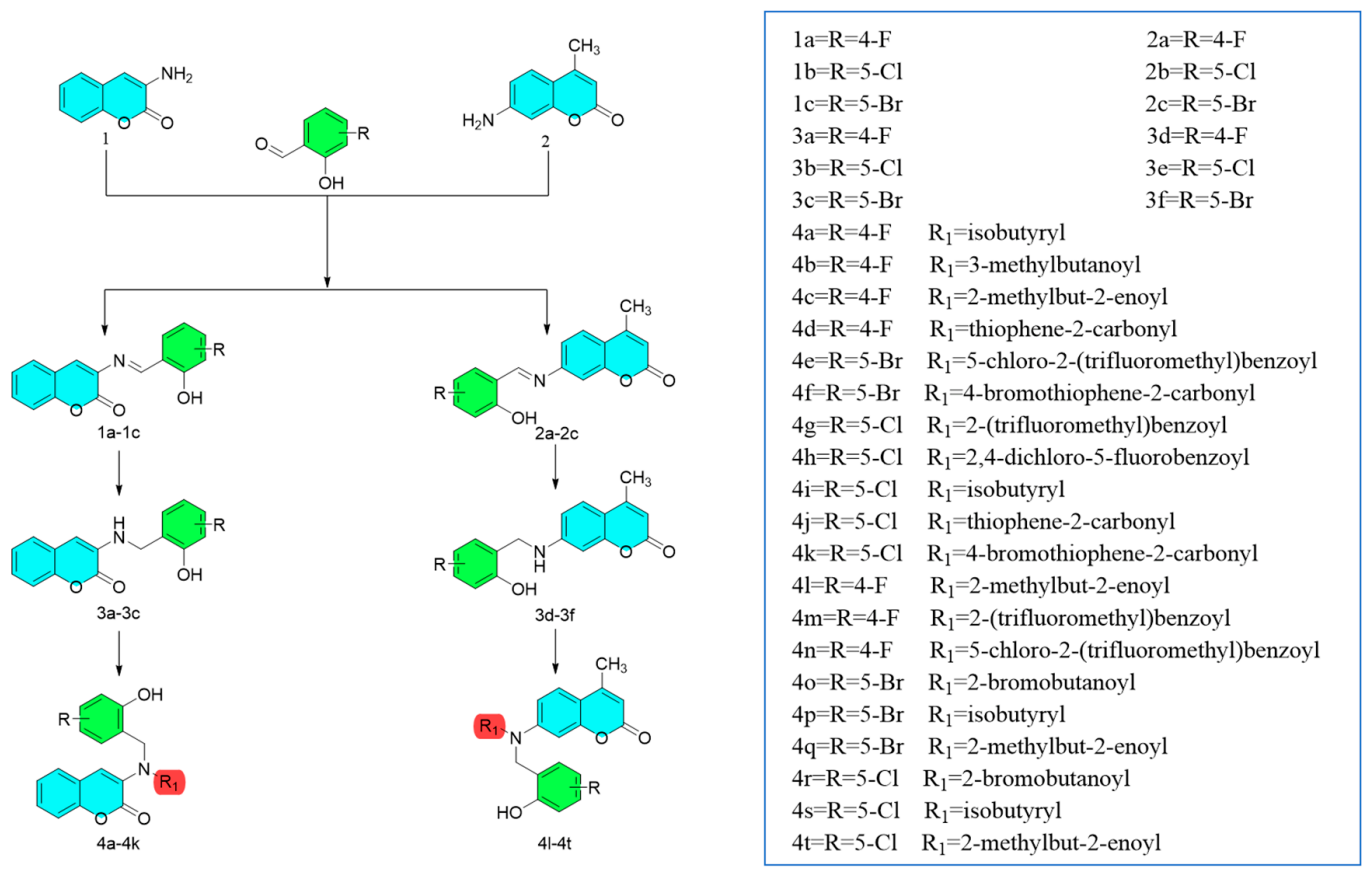
Synthetic route to target compounds.

The target compounds were synthesized from two commercially available compounds, 3-aminocoumarin and 7-amino-4-methylcoumarin as the parent scaffolds for derivatization (Scheme 1). The corresponding coumarin Schiff base intermediates were synthesized in 60–80% yields via aldehyde-amine condensation with commercially available benzaldehyde derivatives. Subsequently, the Schiff base linkages were reduced to C-N single bonds using sodium borohydride (NaBH_4_), yielding free amino groups for the subsequent amide coupling reaction. Finally, the reduced products were coupled with branched-chain or thiophene carboxylic acids using 1-ethyl-3-(3-dimethylaminopropyl)carbodiimide hydrochloride (EDCI) as the coupling reagent and 4-dimethylaminopyridine (DMAP) as the basic catalyst the target compounds were obtained in 50–80% yields.

### 3.2. Biological Evaluation

#### 3.2.1. In Vitro Anti-Tumor Screening

The in vitro inhibition rates of all 26 synthesized target compounds against human esophageal cancer cell lines KYSE70 and KYSE150 were evaluated using the MTT assay (Table 1). Preliminary screening results showed that most compounds exhibited antitumor effects against both cell lines, with compound **4s** demonstrating the highest inhibition rate of 94.71% at 10 μg/mL. Based on these results, the most active compounds were selected for further IC_50_ determination (Table 2). As shown in Table 2, compounds **4s** and **4p** exhibited IC_50_ values of 3.44 and 3.39 μM against KYSE70 cells, respectively.

To assess in vitro cytotoxicity, compounds **4o**, **4p**, **4r**, and **4s** were tested on immortalized human esophageal epithelial cells (NE6-T) (Table 3). All tested compounds showed selective toxicity. Among them, compounds **4p** and **4s**, which displayed the highest anticancer activity, had selectivity indices (SI) of 3.84 and 2.14 (against KYSE70 cells), respectively. Since the SI values of all tested compounds were greater than 1, these compounds exhibit greater cytotoxicity toward cancer cells than toward normal cells. These SI values are comparable to, or even better than, those reported for SAHA under similar conditions [42].

#### 3.2.2. Apoptosis Assay

**Figure 4:**
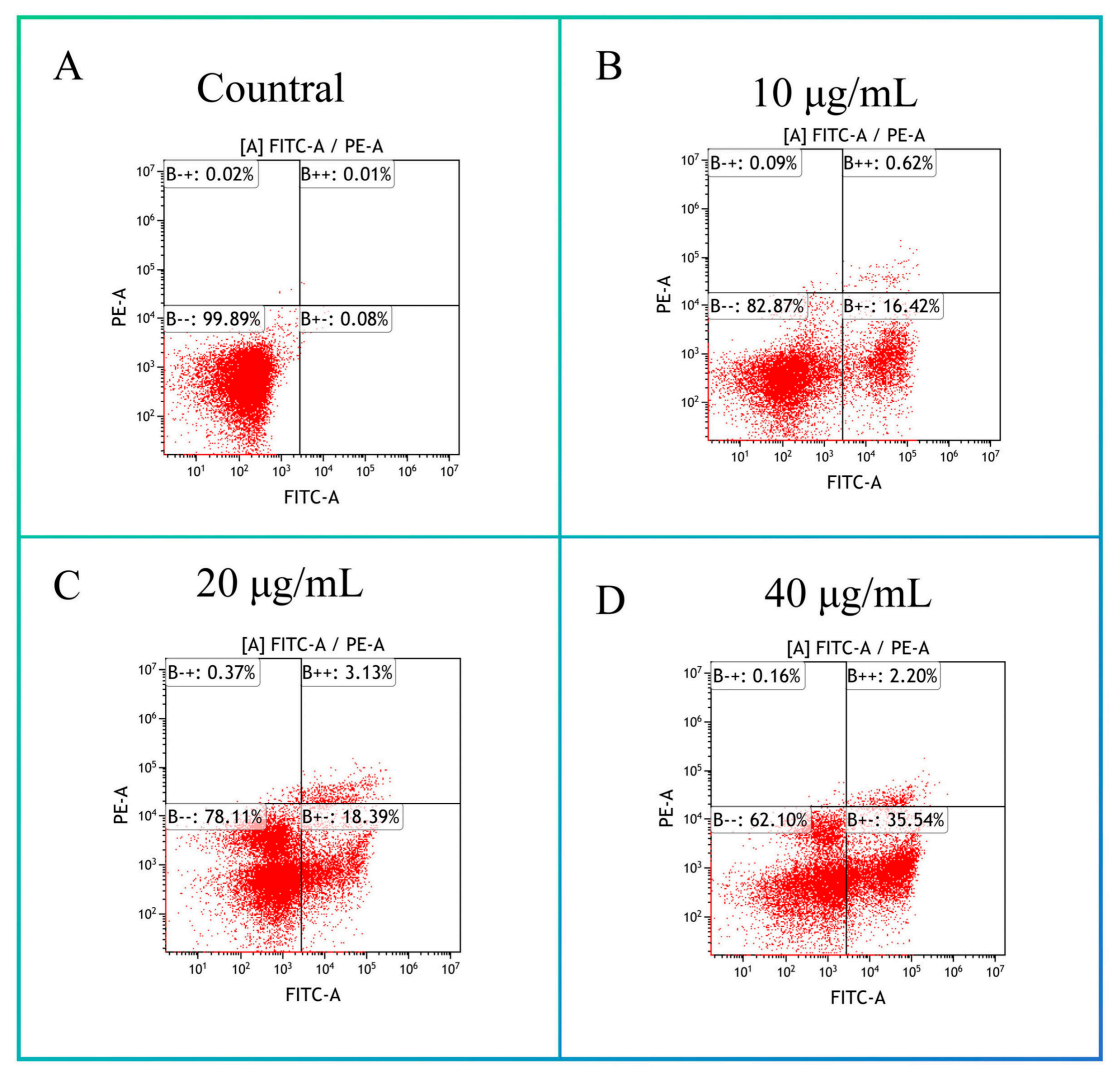
Flow cytometric analysis of apoptosis in KYSE70 cells induced by compound **4s** in a dose-dependent manner. KYSE70 cells exposed to increasing concentrations of compound **4s** (10 μg/mL, 20 μg/mL, and 40 μg/mL) were stained with Annexin V-FITC and PI. (Q4: live cells; Q3: early apoptotic cells; Q2: late apoptotic cells; Q1: necrotic cells). Flow cytometry data were analyzed using FlowJo software.

To evaluate pro-apoptotic effects, compound **4s** was selected for flow cytometric analysis using Annexin V-FITC/propidium iodide (PI) double staining in esophageal squamous cell carcinoma KYSE70 cells. As shown in Figure 4A–D, treatment with compound **4s** markedly increased apoptosis in a concentration-dependent manner. Following treatment with compound **4s** at 10, 20, and 40 μg/mL, the total apoptosis rate (early and late) increased from 0.09% in the control group to 17.04%, 21.52%, and 37.74%, respectively. Notably, the early apoptosis rate increased most markedly, rising from 0.08% in the control group to 16.42%, 18.39%, and 35.54%, respectively. In all cell populations treated with compound **4s**, the proportion of necrotic cells was less than 1%. These results indicate that compound **4s** primarily induces early apoptosis in a concentration-dependent manner, rather than exerting direct cytotoxicity.

### 3.3. D-QSAR Study

**Figure 5:**
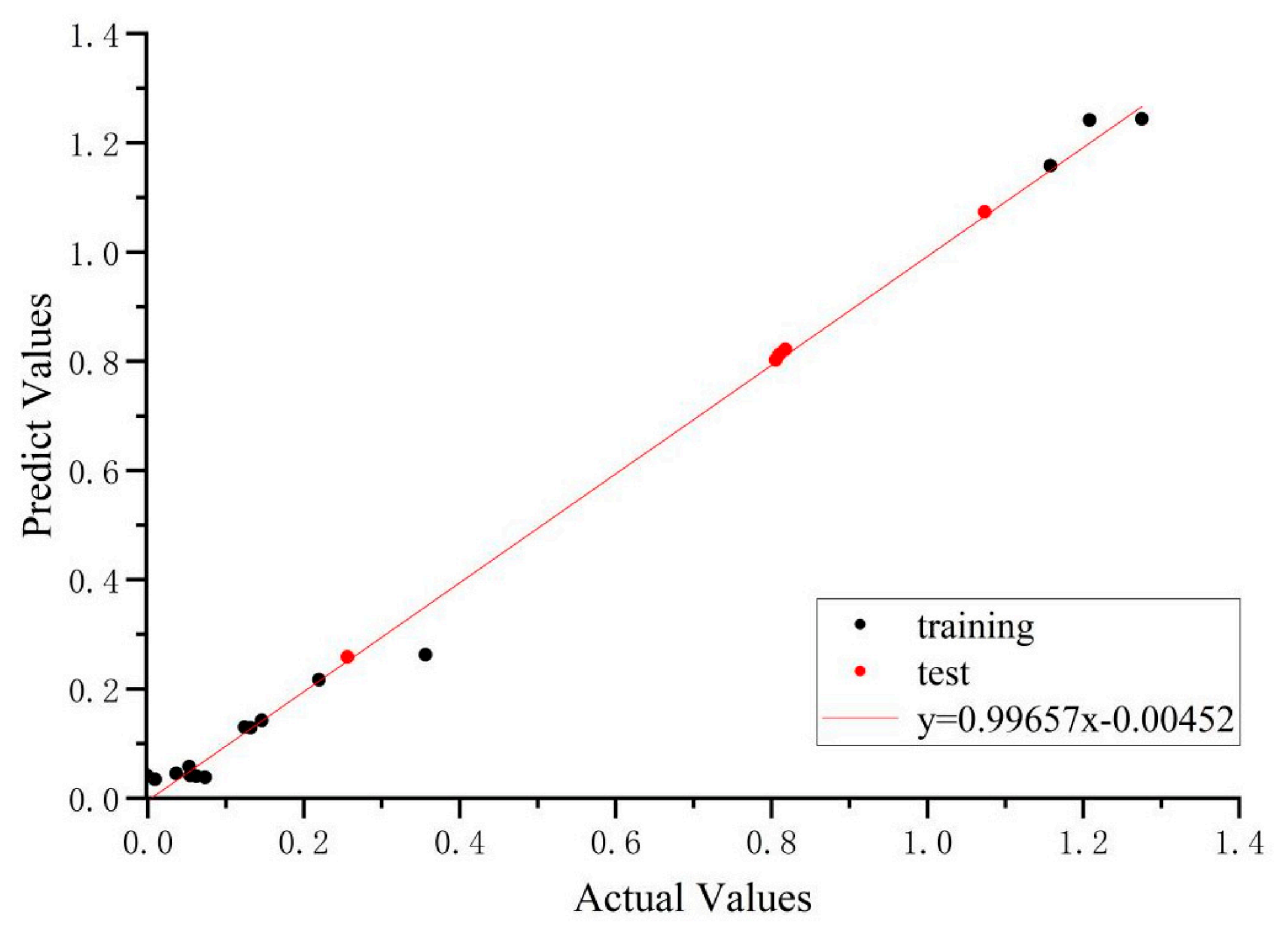
Correlation between predicted and actual values in the CoMFA model.

**Figure 6:**
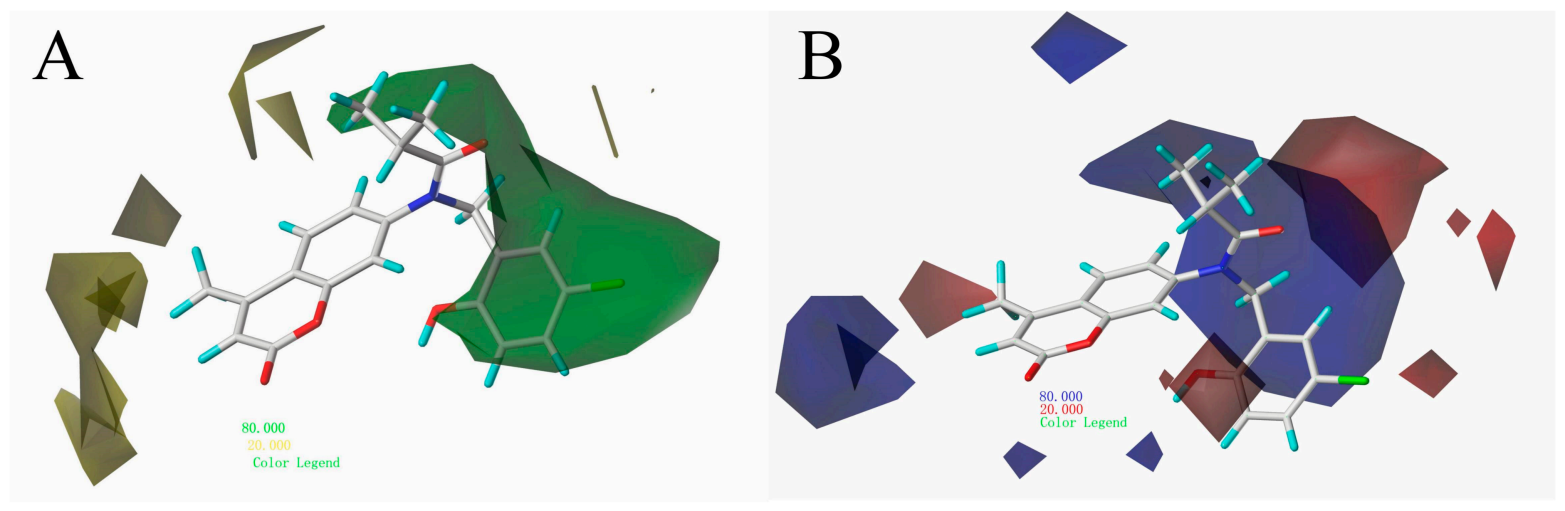
CoMFA contour maps illustrating steric (**A**) and electrostatic (**B**) field contributions.

To further investigate the structure-activity relationships of the target compounds, 3D-QSAR analysis was performed, focusing specifically on activity against esophageal squamous cell carcinoma KYSE70 cells. A CoMFA model was constructed using 20 target compounds, which were divided into a training set comprising 15 compounds and a test set comprising 5 compounds. Figure 5 illustrates the relationship between the actual and predicted biological activities of this series of compounds. Compound **4s**, which exhibited the most potent activity, was selected as the template molecule for alignment. A series of molecular descriptors were calculated to analyze molecular properties, and detailed statistical parameters are presented in Table 4 Partial least squares (PLS) analysis was conducted to establish a linear correlation between the cell biological activity of the compounds and their molecular descriptors. Furthermore, Figure 6 presents the contour maps of the CoMFA model. Panels A and B of Figure 5 illustrate the electrostatic and steric field contributions, respectively, indicating their influence on molecular activity at different positions. The 3D-QSAR model established in this study is based on cellular activity, which is influenced by factors such as cell permeability and drug efflux. Therefore, the results are only applicable to relative comparisons of structure–activity relationships within the same experimental system and cannot be directly extrapolated to predict the intrinsic inhibitory activity against the HDAC protein.

The presence of green contours around the benzene ring, coupled with yellow contours distributed around the introduced amide moiety, accounts for the observed structure-activity relationships: compounds incorporating long-chain carboxylic acids generally exhibited higher activity than those bearing benzoic acid or thiophene carboxylic acid moieties. Specifically, compounds **4s** and **4p** showed substantially higher activity than those featuring benzoyl or thiophene compounds’ carbonyl structures, such as **4m**, **4e**, and **4d**. Yellow contours near the C3 position of the coumarin ring, together with green contours at the C7 position, indicate that decreasing steric hindrance at C3 while increasing steric bulk at C7 enhances biological activity. This finding explains why, within this series, compounds derivatized at the C7 position generally exhibited higher activity than those derivatized at the C3 position. The presence of blue contours at the C3 position of the coumarin ring suggests that introducing electron-withdrawing groups at this position improves biological activity, explaining the higher activity of compound **4k** compared to **4j**. Additionally, blue and yellow contours surrounding the amide position indicate a preference for sterically undemanding structures and electron-donating groups at this location. This accounts for the observed activity trend, in which alkyl chain substituents at this position conferred higher activity than thiophene rings, which in turn outperformed benzene rings. In summary, the 3D-QSAR model we developed fully explains the structure-activity relationships of the compounds we designed and synthesized.

### 3.4. Action Mechanism Study

#### 3.4.1. Eukaryotic Transcriptome Sequencing with Reference Genomes

**Figure 7:**
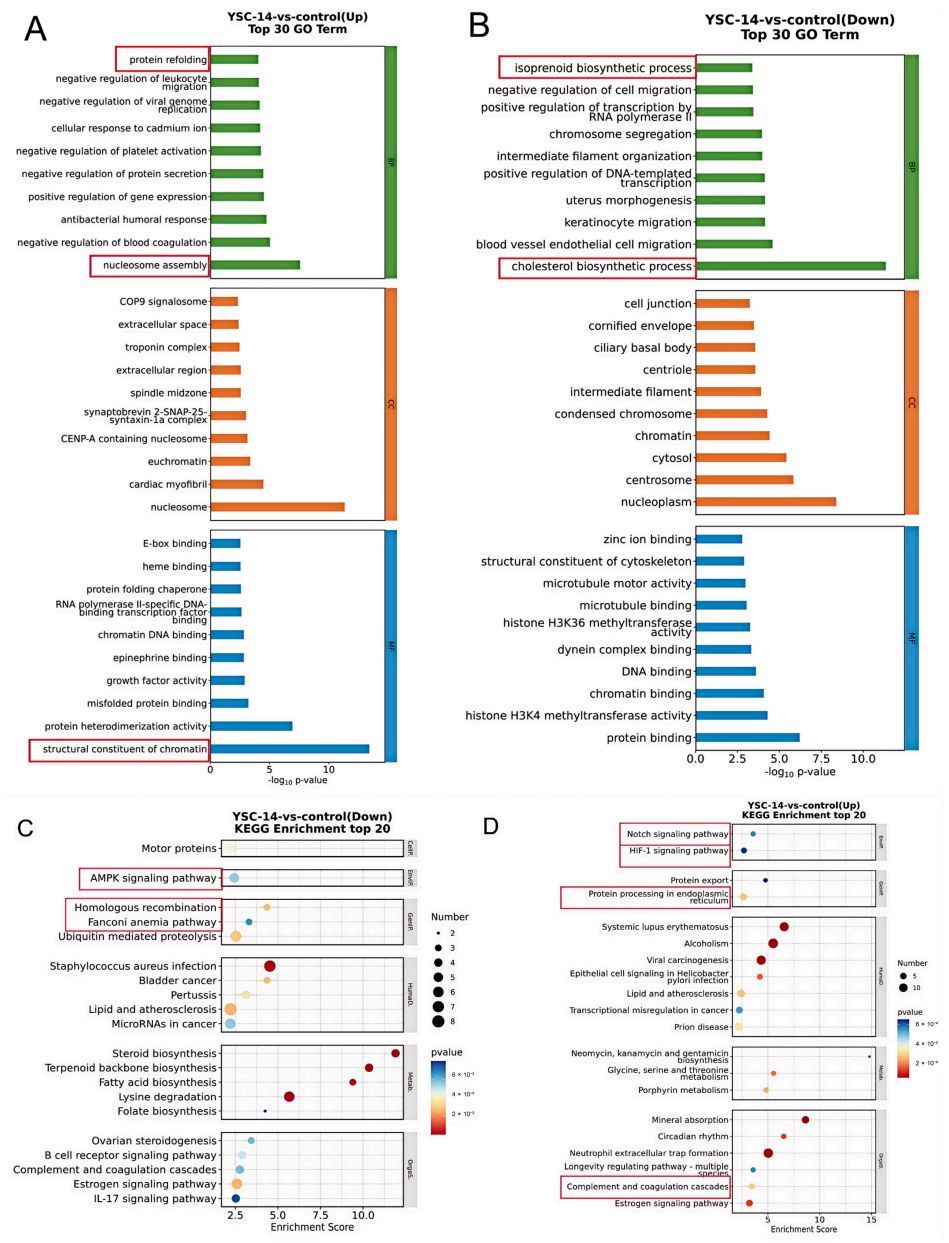

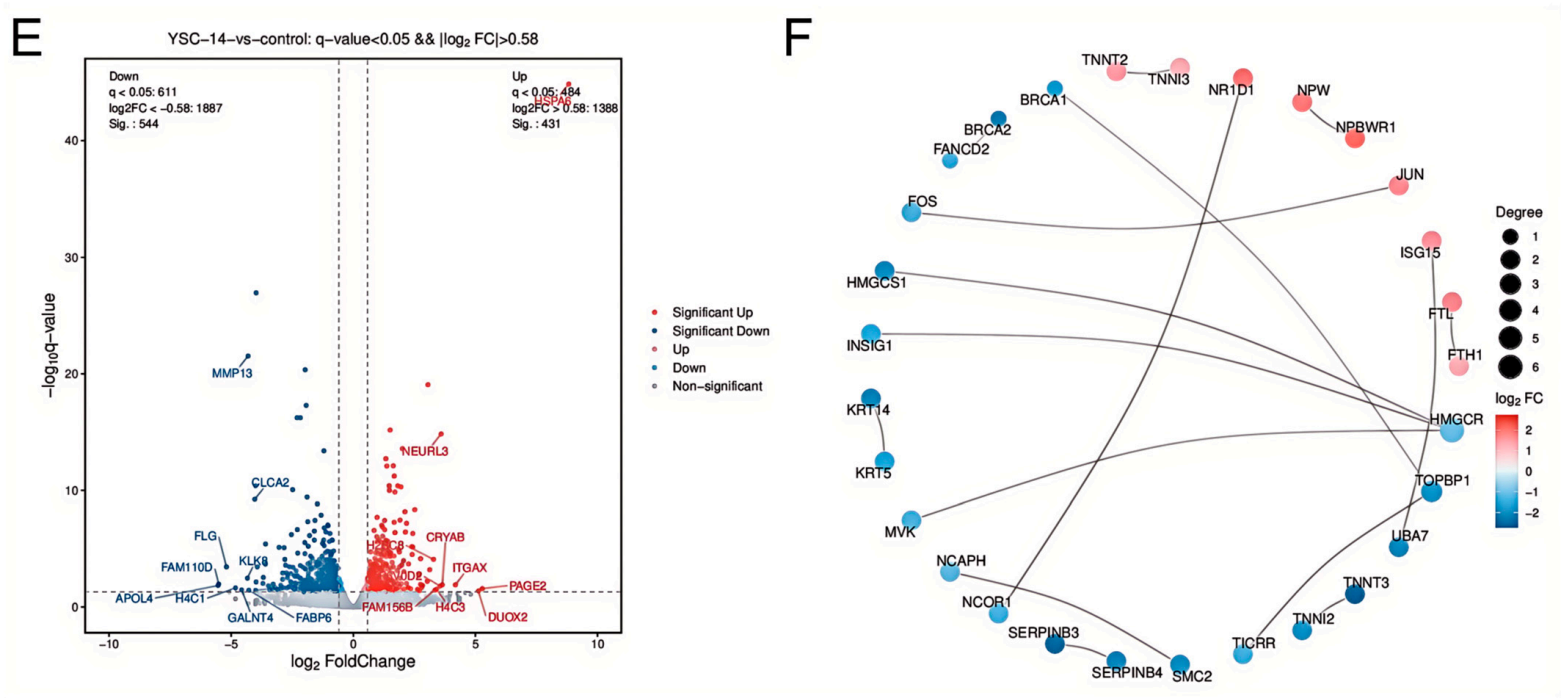
Transcriptome sequencing results. (**A**,**B**) GO enrichment pathways; (**C**,**D**) KEGG enrichment pathways; (**E**) Volcano plot of differentially expressed genes; (**F**) Top 30 protein–protein interaction (PPI) network analysis.

To investigate the mechanism of action of the compounds, the most potent inhibitor, compound **4s**, was selected for transcriptome sequencing. Differential gene expression analysis was employed to identify the potential mechanism of action of the compound. The differentially expressed genes induced by compound **4s** were enriched in multiple biological processes. GO enrichment analysis revealed significant enrichment in chromatin remodeling-related processes, including chromatin binding, nucleosome assembly, and positive regulation of transcription (Figure 7A,B). KEGG enrichment analysis indicated enrichment in the Notch signaling pathway and transcriptional dysregulation (Figure 7B,C). Additionally, pathways related to inhibition of DNA damage repair (e.g., homologous recombination and the Fanconi anemia pathway), endoplasmic reticulum stress response, and metabolic reprogramming were also enriched. Collectively, these results constitute a typical downstream regulatory network of HDAC inhibitors.

Zn^2+^ ion at the HDAC active site, a hallmark feature of HDAC inhibition.

The volcano plot of differentially expressed genes (Figure 7E) and protein–protein interaction (PPI) network analysis (Figure 7F) further supported the role of compound **4s** as an HDAC inhibitor. Under stringent thresholds (q < 0.05, |log_2_FC| > 0.58), treatment with **4s** resulted in significant differential expression of numerous genes. Notably, the invasion-associated gene MMP13 was significantly downregulated, while the stress response marker genes CRYAB and NEURL3 were significantly upregulated. In the PPI network, core DNA repair genes BRCA1, BRCA2, and FANCD2 formed a synergistic interaction module. Collectively, these findings suggest that compound **4s** may exert a dual effect at the transcriptomic level through inhibition of HDAC activity, characterized by activation of stress adaptation programs and shutdown of growth and proliferation programs. This dual effect, mediated by the synergistic actions of metabolic reprogramming, genomic instability, and inactivation of proliferative signaling, ultimately induces tumor cell death. These results are consistent with previously reported transcriptomic profiles of HDAC inhibitors, providing indirect evidence that compound **4s** could exert its anti-esophageal squamous cell carcinoma activity through HDAC inhibition [47].

#### 3.4.2. In Vitro Validation of HDAC Enzymatic Activity

To verify whether the synthesized compounds exert their antiproliferative effects by inhibiting HDAC enzyme activity, we performed in situ enzyme activity assays in KYSE70 cells using a fluorescent HDAC activity assay kit. After treating the cells with different concentrations of the compounds for 48 h, the residual HDAC activity within the cells was measured, using the DMSO-treated group as the negative control and the broad-spectrum HDAC inhibitor SAHA as the positive control. The results are shown in Table 5. Both compound **4s** and **4p** exhibited concentration-dependent inhibition of HDAC enzymatic activity. As the concentration increased from 0.5 μg/mL to 8 μg/mL, the inhibition rates of HDAC1 by compounds **4s** and **4p** were 24.02 to 90.16% and 42.3 to 97.54%. the IC_50_ values for the inhibition of HDAC activity by compounds **4s** and **4p** were calculated to be 3.07 μg/mL and 2.39 μg/mL, respectively. For reference, the IC_50_ of SAHA for HDAC activity in this experimental system was 3.64 μg/mL. These results directly demonstrate that compounds **4s** and **4p** can effectively inhibit the catalytic activity of intracellular HDACs, indicating that compounds **4s** and **4p** are HDAC inhibitors.

#### 3.4.3. Molecular Docking

**Figure 8:**
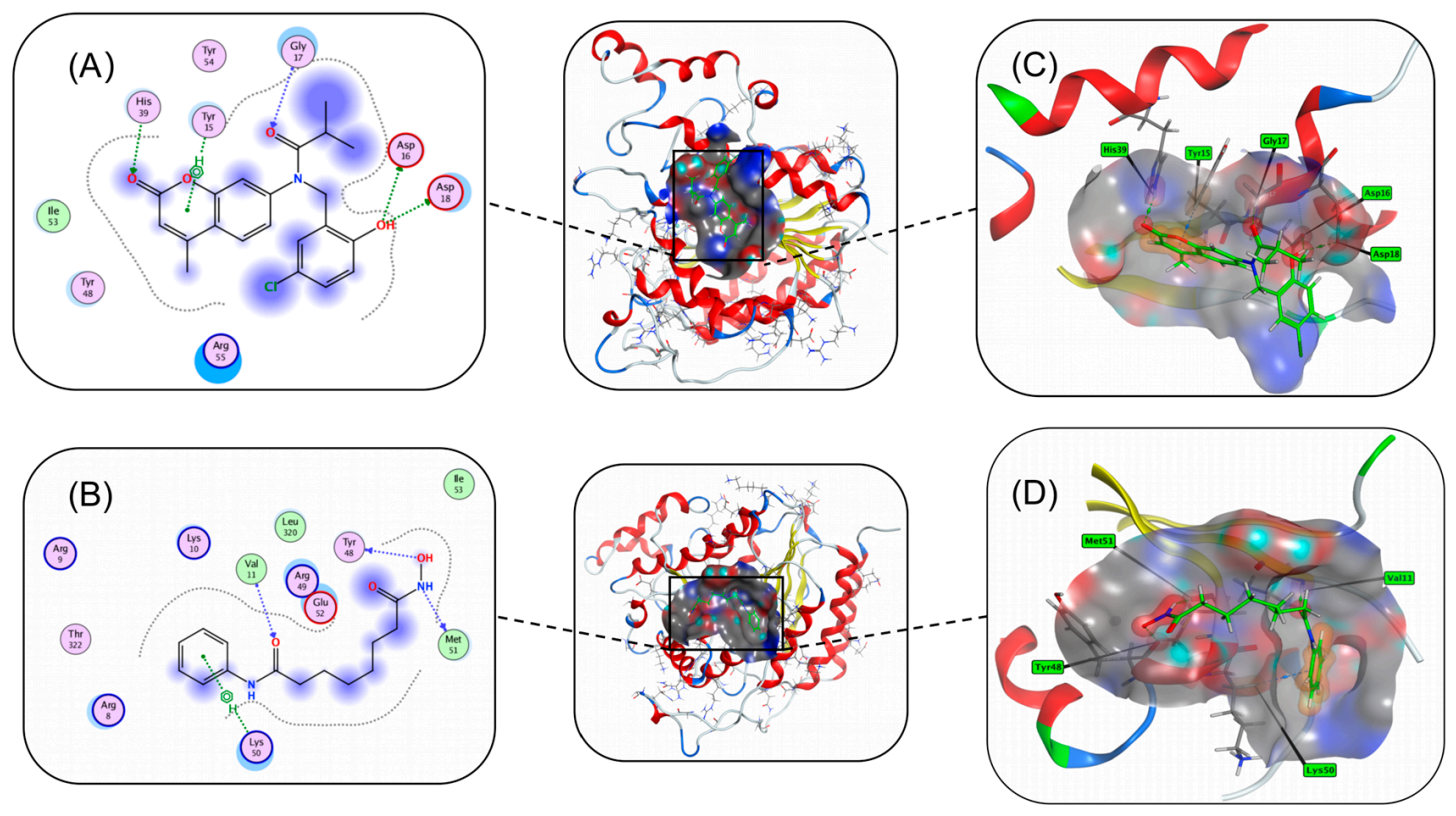
Molecular docking of compound 4s and SAHA with HDAC1 (PDB: 5ICN). (**A**) 2D diagram of 4s-5ICN; (**B**) 2D diagram of SAHA-5ICN; (**C**) 3D diagram of 4s-5ICN; (**D**) 3D diagram of SAHA-5ICN.

To elucidate the potential binding mode and to evaluate its selectivity for HDAC isoforms of compound **4s**, molecular docking studies were conducted using the crystal structure of HDAC1 (PDB ID: 5ICN) [48], HDAC2(PDB ID: 4LXZ) [49] and HDAC6 (PDB ID: 5EDU) [50] with the well-known HDAC inhibitor SAHA serving as a positive control (The docking results for HDAC2 and HDAC6 are presented in the Supporting Information.). As illustrated in Figure 8, compound **4s** adopted a binding mode analogous to that of SAHA. Specifically, the carbonyl oxygen of the coumarin pyran ring and the hydroxyl group on the benzene ring of compound **4s** formed hydrogen bonds with His39 and Asp16, respectively. Meanwhile, the hydroxamic acid moiety of SAHA formed a strong coordination interaction with Tyr48. Notably, both compounds targeted the catalytic However, distinct interaction patterns were also observed. The pyran ring of compound **4s** formed *π-π* stacking interactions with Tyr15. This contrasts with SAHA, for which the benzene ring and alkyl chain participate in extensive electrostatic and hydrogen bonding networks with a positively charged patch composed of Arg8, Arg9, Lys10, and Arg49. Rather than relying on such broad interactions with the arginine patch, compound **4s** adopted a distinct surface-binding strategy: its halogenated aromatic ring formed *π-π* stacking with Tyr54, while the chlorine atom potentially could establish a halogen bond with Arg55. These unique interactions may contribute to its distinctive binding affinity and potential isoform selectivity.

Furthermore, structural differences in the linker region influenced their access to the hydrophobic channel. SAHA, with its canonical six-carbon alkyl chain, inserts deeply into the hydrophobic tunnel formed by Met51 and Leu320. In contrast, compound **4s**, featuring a shorter alkyl linker, occupies a shallower hydrophobic pocket defined by residues such as Ile53. This configuration positions its cap group closer to the catalytic domain, thereby altering contact patterns with hydrophobic channel residues. Such differences may result in distinct kinetic inhibition profiles compared to SAHA.

#### 3.4.4. Molecular Dynamics Simulation Study

**Figure 9:**
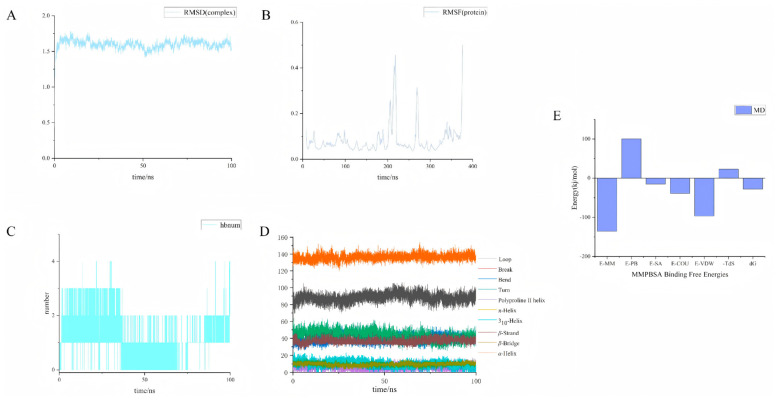
Molecular dynamics (MD) simulation of compound 4s with HDAC1 (PDB: 5ICN). (**A**) Root mean square deviation (RMSD); (**B**) Root mean square fluctuation (RMSF); (**C**) Hydrogen bond analysis; (**D**) Dictionary of secondary structure of proteins (DSSP); (**E**) Molecular mechanics/Poisson–Boltzmann surface area (MM/PBSA) calculations: E-MM, molecular mechanics energy; E-PB, polar solvation energy; E-SA, non-polar solvation energy; E-COU, electrostatic interaction energy; E-VDW, van der Waals interaction energy; –TΔS, negative temperature times entropy change; ΔG, Gibbs free energy of binding.

To investigate the binding stability of compound 4s with HDAC1 (PDB: 5ICN), a 100 ns molecular dynamics simulation was performed. RMSD analysis of the protein–ligand complex (Figure 9A) showed a rapid increase during the first 20 ns, followed by stable fluctuations between 1.2 and 1.7 nm over the remaining 80 ns, with an average RMSD of 1.60 ± 0.07 nm, indicating that the complex reached dynamic equilibrium and remained globally stable. RMSF analysis (Figure 9B) revealed that most residues, including the key catalytic residues His140, Asp176, and His178, exhibited low fluctuations (<0.15 nm), preserving the active-site geometry. In contrast, three flexible regions were identified—residues 202–221, 265–272, and the C-terminus—which are distant from the binding pocket and likely involved in loop motions or protein protein interactions. DSSP analysis (Figure 9D) further confirmed that the secondary structure of HDAC1 remained largely intact during the simulation, with α-helices (130–145 residues) and β-strands (30–45 residues) as the dominant elements, and only local fluctuations in random coils corresponding to the flexible loops observed in RMSF.

Hydrogen bond analysis (Figure 9C) indicated that compound 4s formed on average only 1.08 ± 0.83 hydrogen bonds with HDAC1, suggesting that hydrogen bonding plays a secondary, auxiliary role in complex stabilization. This is consistent with the MM/PBSA binding free energy calculation (Figure 9E), which yielded a ΔG of −27.86 kJ/mol. Energy decomposition showed that van der Waals interactions (−96.70 kJ/mol) and nonpolar solvation energy (−15.25 kJ/mol) were the major favorable contributors, while polar solvation energy was strongly unfavorable (+100.16 kJ/mol). The large positive polar solvation term indicates substantial desolvation of the ligand upon binding, a hallmark of hydrophobic association. Collectively, these results demonstrate that compound 4s forms a stable complex with HDAC1 primarily driven by hydrophobic effects and tight van der Waals packing, in good agreement with reported binding modes of HDAC inhibitors [51].

## 4. Conclusions

Based on a pharmacophore model, a series of coumarin-benzylamine hybrid molecules were designed and synthesized as HDAC inhibitors. Notably, among the synthesized compounds, compound **4s** exhibited the most potent inhibitory activity against KYSE70 cells, with an IC_50_ value of 3.44 μM. Flow cytometry analysis revealed that the compound’s inhibitory effect on cell viability was concentration-dependent. Following treatment with compound **4s**, GO and KEGG enrichment analyses identified pathways that are known to be associated with the mechanism of action of HDAC inhibitors, including chromatin assembly, transcriptional dysregulation, and DNA repair. Enzyme activity assays confirmed that compounds **4s** and **4p** exhibited significant HDAC inhibitory activity. In this experimental system, both compounds **4s** and **4p** exhibited enzymatic activity superior to that of the positive control, SAHA. To explore the binding mode and isoform selectivity of compound 4s with HDAC proteins, the known HDAC inhibitor SAHA was selected as a control, and molecular docking was performed against HDAC1, HDAC2, and HDAC6. The results indicated that compound 4s is a class I HDAC inhibitor and exhibits the typical features of HDAC inhibitors, with potential binding interactions in the cap, ZBG, and linker regions of the HDAC protein. A 100 ns molecular dynamics simulation indicated that compound **4s** primarily interacts with the HDAC enzyme via van der Waals forces and hydrophobic interactions during binding, which is consistent with the results of molecular dynamics simulations of previously published HDAC inhibitors. Finally, a 3D-QSAR CoMFA model was constructed to elucidate the structure-activity relationship of the synthesized compounds.

In summary, a series of novel HDAC inhibitors were designed and synthesized in this study. Their mechanism of action and structure-activity relationships were investigated through transcriptomics, enzyme activity validation, molecular docking, molecular dynamics simulation and molecular modeling, demonstrating their potential as targeted therapeutic agents for cancer. Therefore, compound **4s** could serve as a promising lead molecule for the future development of HDAC inhibitors, which could provide new strategies for the development of novel anti-tumor agents.

## Figures and Tables

**Table 1:** In Vitro Inhibitory Activity of Target Compounds at Different Concentrations Against KYSE70 and KYSE150 Cell Lines Using the MTT Assay.

Compd	KYSE70 ^a^				KYSE150 ^b^			
	24 h		48 h		24 h		48 h	
	40.00 (μg/mL)	10 (μg/mL)	40.00 (μg/mL)	10 (μg/mL)	40.00 (μg/mL)	10 (μg/mL)	40.00 (μg/mL)	10 (μg/mL)
**3a**	75.71 ± 4.78	18.10 ± 1.96	85.31 ± 2.81	19.99 ± 1.79	22.35 ± 1.19	11.83 ± 1.91	50.39 ± 1.18	25.81 ± 1.52
**3b**	75.68 ± 2.74	15.16 ± 1.43	83.65 ± 1.08	2.99 ± 0.89	26.35 ± 1.89	16.74 ± 2.34	28.17 ± 1.77	19.21 ± 0.89
**3c**	84.55 ± 3.78	13.16 ± 3.12	91.14 ± 0.26	21.90 ± 1.10	19.56 ± 0.17	15.53 ± 1.73	37.01 ± 2.42	34.40 ± 1.99
**3d**	24.31 ± 3.09	14.27 ± 2.94	11.83 ± 2.09	24.18 ± 2.24	17.91 ± 1.51	11.84 ± 1.41	26.68 ± 1.69	11.00 ± 0.52
**3e**	79.04 ± 2.49	72.94 ± 0.86	86.47 ± 1.31	90.64 ± 2.49	51.65 ± 2.31	31.41 ± 2.48	66.76 ± 1.99	36.25 ± 1.07
**3f**	70.11 ± 3.34	60.21 ± 3.88	82.45 ± 2.98	67.35 ± 1.74	45.33 ± 2.98	29.37 ± 2.31	57.35 ± 0.89	31.74 ± 2.42
**4a**	40.66 ± 2.64	20.55 ± 2.65	43.42 ± 1.94	26.07 ± 2.07	9.95 ± 0.42	4.79 ± 0.36	10.18 ± 1.62	5.01 ± 1.63
**4b**	15.92 ± 2.22	8.16 ± 1.95	35.38 ± 1.59	28.64 ± 3.58	8.40 ± 2.37	4.76 ± 0.92	36.95 ± 1.92	14.82 ± 2.41
**4c**	9.84 ± 2.03	1.94 ± 0.76	21.81 ± 2.75	11.52 ± 2.79	4.50 ± 0.43	1.41 ± 0.73	8.52 ± 0.21	2.02 ± 0.32
**4d**	42.89 ± 3.06	24.51 ± 2.27	32.55 ± 1.64	25.16 ± 1.66	25.02 ± 1.04	14.54 ± 1.74	26.19 ± 1.28	17.80 ± 1.35
**4e**	16.42 ± 2.56	4.25 ± 1.15	21.75 ± 1.52	7.88 ± 1.17	13.96 ± 0.71	3.91 ± 0.05	25.95 ± 1.23	8.98 ± 2.81
**4f**	85.46 ± 3.29	53.71 ± 1.87	85.82 ± 1.15	66.15 ± 0.79	57.46 ± 2.46	31.84 ± 0.64	72.13 ± 1.38	20.42 ± 1.10
**4g**	25.94 ± 2.82	14.46 ± 1.85	18.57 ± 1.42	12.13 ± 1.42	10.87 ± 1.08	2.79 ± 0.92	12.02 ± 2.84	1.26 ± 2.13
**4h**	94.45 ± 1.69	49.54 ± 2.61	87.64 ± 2.05	39.83 ± 2.83	50.73 ± 2.96	8.31 ± 0.75	62.84 ± 2.29	19.24 ± 1.30
**4i**	3.15 ± 0.07	0.54 ± 0.24	5.41 ± 1.25	2.32 ± 0.67	3.24 ± 1.33	1.22 ± 0.53	4.11 ± 1.45	0.45 ± 0.09
**4j**	24.56 ± 1.98	10.42 ± 2.42	26.33 ± 2.48	13.42 ± 1.22	8.96 ± 2.09	2.97 ± 0.47	14.09 ± 9.90	6.41 ± 1.53
**4k**	91.73 ± 0.50	55.91 ± 1.83	90.53 ± 1.59	42.13 ± 2.24	66.59 ± 1.29	20.70 ± 1.74	79.78 ± 0.06	8.46 ± 2.75
**4l**	60.84 ± 3.92	22.59 ± 2.12	84.06 ± 1.60	24.31 ± 1.96	63.46 ± 2.42	8.65 ± 1.46	84.35 ± 1.20	15.35 ± 1.85
**4m**	26.78 ± 1.82	7.84 ± 1.15	16.07 ± 1.54	15.94 ± 1.58	22.62 ± 2.05	24.54 ± 2.56	28.40 ± 2.45	26.95 ± 1.95
**4n**	9.66 ± 3.93	1.44 ± 0.67	ND	ND	6.49 ± 5.90	1.41 ± 1.34	6.91 ± 1.21	0.47 ± 1.35
**4o**	90.03 ± 0.69	81.49 ± 1.68	92.17 ± 1.20	93.04 ± 1.31	86.79 ± 0.69	48.92 ± 2.67	98.59 ± 0.60	57.59 ± 2.68
**4p**	84.5 ± 1.60	79.75 ± 2.76	93.42 ± 0.63	93.80 ± 1.82	84.56 ± 0.85	45.72 ± 2.09	92.51 ± 1.75	63.40 ± 0.98
**4q**	64.26 ± 2.76	66.67 ± 1.32	85.75 ± 2.97	80.47 ± 3.49	49.95 ± 2.92	14.82 ± 1.41	54.60 ± 2.52	55.69 ± 1.52
**4r**	83.04 ± 1.40	74.53 ± 2.69	90.02 ± 1.06	92.64 ± 3.17	69.59 ± 1.01	38.29 ± 2.73	74.21 ± 1.92	61.59 ± 2.95
**4s**	83.90 ± 3.51	83.00 ± 2.90	94.68 ± 1.33	94.71 ± 2.35	55.59 ± 0.07	45.07 ± 3.46	76.21 ± 1.62	69.59 ± 1.86
**4t**	66.44 ± 1.45	51.34 ± 1.29	42.72 ± 2.99	25.12 ± 1.95	1.86 ± 0.04	18.61 ± 1.39	31.86 ± 2.72	27.84 ± 2.04

Data are presented as the mean of three independent experiments performed in quadruplicate. ND—not detected. ^a,b^ Human esophageal squamous cell carcinoma cell lines.

**Table 2:** In vitro cytotoxicity of compounds **3e**, **4o**, **4p**, **4r** and **4s** against KYSE70 cells.

Compound	IC_50_ (μM) ^c^	95% Confidence Interval	Recurrence Relation	R^2^
**3e**	7.08	6.25–7.97	Y = −1.39 + 3.58 × X	0.98
**4o**	4.69	4.01–5.44	Y = −0.97 + 2.70 × X	0.97
**4p**	3.39	2.84–4.11	Y = −0.44 + 2.90 × X	0.97
**4r**	5.16	4.45–5.94	Y = −1.12 + 2.97 × X	0.99
**4s**	3.44	2.83–4.12	Y = −0.32 + 2.59 × X	0.98

^c^ IC_50_ values are the concentrations (μM) required for 50% inhibition of cancer cell growth.

**Table 3:** In vitro cytotoxicity of compounds **4o**, **4p**, **4r**, and **4s** against NE6-T.

Conpound	IC_50_ (μM)	95% Confidence Interval	Recurrence Relation	R^2^	Selectivity Index ^d^
**4o**	12.11	11.04–13.35	y = −4.25 + 5.33 × x	0.98	2.58
**4p**	13.04	9.11–20.92	y = −2.09 + 2.71 × x	0.85	3.84
**4r**	7.34	6.30–8.56	y = −1.37 + 2.57 × x	0.99	1.42
**4s**	7.39	6.22–8.76	y = −1 + 2.24 × x	0.97	2.14

^d^ to IC_50_ in cancer cells (KYSE70). SI > 1 indicates preferential cytotoxicity toward cancer cells. Selectivity index (SI) was calculated as the ratio of IC_50_ in normal cells (NE6-7).

**Table 4:** Statistical parameters of the CoMFA model.

Statistical Parameter	CoMFA	Verification Standard
q^2 a^	0.748	>0.5
NOC ^b^	5	
R^2 c^	0.998	>0.9
SEE ^d^	0.029	
F ^e^	746.588	>100
fraction of field contributionsg ^g^		
Steric	0.676	
Electrostatic	0.324	

^a^ Cross-validated correlation coefficient (q^2^). ^b^ Optimal number of components (ONC). ^c^ Non-cross-validated correlation coefficient (r^2^). ^d^ Standard error of estimate (SEE). ^e^ Fisher’s F value. ^g^ Steric and electrostatic field contributions.

**Table 5:** In vitro activity of compounds **4s**, **4p** and SAHA against HDAC enzymes.

Compound	IC_50_ (μM)	95% Confidence Interval	Recurrence Relation	R^2^
**4s**	9.98	8.29–11.92	y = 2.5 + 96.2/[1 + (3.3.87/x)^1.58^]	0.94
**4p**	5.55	4.60–6.72	y = 40.2 + 59.3/[1 + (3.72/x)^3.85^]	0.99
SAHA	13.77	9.91–19.14	y = 30.1 + 44.9/[1 + (4.63/x)^1.65^]	0.97

## Data Availability

The original contributions presented in this study are included in the article/supplementary Material. Further inquiries can be directed to the corresponding author(s).

## Supplementary Materials

The following supporting information can be downloaded at: https://www.mdpi.com/article/10.3390/biom16070978/s1, Figure S1: Molecular docking of compound 4s and SAHA with HDAC2 and HDAC6 (PDB: 4LXZ and 5EDU). (A) 2D diagram of 4s-4LXZ; (B) 2D diagram of SAHA-4LXZ; (C) 2D diagram of 4s-5EDU; (D) 2D diagram of SAHA-5EDU; (E) 3D diagram of 4s-4LXZ; (F) 3D diagram of SAHA-4LXZ; (G) 3D diagram of 4s-5EDU; (H) 3D diagram of SAHA-5EDU.; Table S1: Molecular docking scores of compound 4s with three HDAC proteins (HDAC1, HDAC2, and HDAC6).
