# Supplementary material for: Design, Synthesis, and Antitumor Activities of Novel Coumarin-Based Histone Deacetylase Inhibitors
Source: Biomolecules. 2026 Jul 3;16(7):978. doi: 10.3390/biom16070978 (PMC13406824; doi:10.3390/biom16070978)
Supplement: Supplementary file 1 [file biomolecules-16-00978-s001.zip › biomolecules-4276741-supplementary.pdf]

Design, Synthesis, and Antitumor Activities of Novel Coumarin-Based Histone Deacetylase Inhibitors

Sichang Yan[1]、Jie Chang[1]、Dongyu Lei[1]、Xiangyang Lv[1]、Yanzhuo Li[1]、Yue Zhuo[1]、Lu Jin[1]、Le Pan[1,*]

1. College of Chemistry and Chemical Engineeing, Xinjiang Agricultural University, Urumqi 830052, China

2. Department of Physiology, Preclinical School, Xinjiang Medical University, Urumqi 830011, China

* Corresponding authors: chempan03@163.com

Data of ^{1}H NMR, ^{13}C NMR

3-((2-Hydroxy-4-fluorophenyl)imino)coumarin (1a)

Yellow solid; yield: 83%; mp:210℃-212℃.

^{1}H NMR (600 MHz, $CDCl_3$) δ 9.46 (s, 1H), 7.70 (s, 1H), 7.60 – 7.52 (m, 2H), 7.44 – 7.31 (m, 3H), 6.72 – 6.64 (m, 2H).

^{13}C NMR (150 MHz, $CDCl_3$) *δ* 166.55, 164.03, 158.26, 152.50, 135.56, 134.78, 131.92, 131.71, 128.43, 125.34, 119.89, 116.83, 107.89, 107.73, 104.91, 104.76 .

3-((2-Hydroxy-5-bromophenyl)imino)coumarin (1b)

Yellow solid; yield: 63%; mp:210℃-212℃.

^{1}H NMR (600 MHz, $CDCl_3$) δ 12.97 (s, 1H, OH), 9.46 (s, 1H), 7.75 (s, 1H), 7.59 (d, *J* = 9.8 Hz, 1H), 7.58 – 7.55 (m, 2H), 7.47 (d, *J* = 8.9 Hz, 1H), 7.39 (d, *J* = 7.9 Hz, 1H), 7.34 (t, *J* = 8.2 Hz, 1H), 6.92 (d, *J* = 8.9 Hz, 1H).

^{13}C NMR (150 MHz, DMSO-d_6) δ 163.56, 159.69, 152.33, 138.85, 136.57, 133.53, 131.78, 130.95, 128.97, 125.30, 125.18, 119.65, 116.40, 110.64.

3-((2-Hydroxy-5-chlorophenyl)imino)coumarin (1c)

Yellow solid; yield: 69%; mp:164℃-167℃.

^{1}H NMR (600 MHz, DMSO-d_6) δ 10.97 (s, 1H), 9.22 (s, 1H), 7.59 (s, 1H), 7.55 (d, *J* = 8.8 Hz, 1H), 7.41 (t, *J* = 7.3 Hz, 1H), 7.27 (d, *J* = 7.8 Hz, 1H), 7.24 – 7.19 (m, 2H), 7.03 (d, *J* = 8.7 Hz, 1H), 6.71 (s, 1H).

^{13}C NMR (150 MHz, DMSO-d_6) δ 159.77, 157.94, 148.17, 136.03, 133.60, 127.66, 125.62, 125.09, 124.78, 123.75, 123.61, 122.08, 119.80, 115.71, 107.91.

7-((4-Fluoro-2-hydroxyphenyl)imino)-4-methylcoumarin (2a)

Orange-yellow solid; yield: 86%; mp:201℃-202℃.

^{1}H NMR (600 MHz, $CDCl_3$) δ 9.84 (s, 1H), δ 8.62 (s, 1H), 7.65 (d, *J* = 8.3 Hz, 1H), 7.42 (t, *J* = 7.5 Hz, 1H), 7.24 – 7.16 (m, 2H), 6.74 (d, *J* = 10.5 Hz, 1H), 6.70 (t, *J* = 8.4 Hz, 1H), 6.29 (s, 1H), 2.46 (s, 3H, CH_3).

^{13}C NMR (150 MHz, $CDCl_3$) δ 165.86, 163.78, 161.00, 154.86, 152.30, 151.65, 135.02, 126.02, 118.98, 118.64, 114.98, 109.04, 107.97, 107.82, 104.85, 19.08.

8-((2-Hydroxy-5-bromophenyl)imino)-4-methylcoumarin (2b)

Yellow solid; yield: 76%; mp:245℃-246℃.

^{1}H NMR (600 MHz, $CDCl_3$) δ 10.94 (s, 1H), 8.59 (s, 1H), 7.66 (d, *J* = 8.2 Hz, 1H), 7.56 (s, 1H), 7.50 (d, *J* = 6.3 Hz, 1H), 7.24 – 7.19 (m, 2H), 6.96 (d, *J* = 8.8 Hz, 1H), 6.30 (s, 1H), 2.47 (s, 3H).

^{13}C NMR (150 MHz, $CDCl_3$) δ 163.49, 160.92, 160.62, 154.81, 151.38, 137.01,

135.07, 126.10, 120.18, 119.83, 119.27, 118.51, 115.17, 111.22, 109.28, 19.10.

7-((2-Hydroxy-5-chlorophenyl)imino)-4-methylcoumarin (2c)

Yellow solid; yield: 70%; mp:243℃-245℃. ^{1}H NMR (600 MHz, $CDCl_3$) δ 12.78 (s, 1H), 8.59 (s, 1H), 7.66 (d, *J* = 7.5 Hz, 1H), 7.44 – 7.35 (m, 2H), 7.24 – 7.18 (m, 2H), 7.01 (d, *J* = 8.3 Hz, 1H), 6.30 (s, 1H), 2.47 (s, 3H).

^{13}C NMR (150 MHz, DMSO-d_6) δ 163.17, 158.67, 153.69, 133.20, 130.62, 126.32, 122.63, 120.58, 118.62, 118.12, 113.62, 108.57, 17.95.

4-((2-Hydroxy-4-fluorophenyl)amino)coumarin (3a)

Dark yellow solid; yield: 89%; mp:191℃-192℃. ^{1}H NMR (600 MHz, DMSO-d_6) δ 10.24 (s, 1H), 7.37 (d, *J* = 5.4 Hz, 1H), 7.27 (d, *J* = 8.0 Hz, 1H), 7.22 – 7.17 (m, 2H), 6.65 (d, *J* = 8.2 Hz, 1H), 6.57 – 6.53 (m, 1H), 6.45 (d, *J* = 4.5 Hz, 2H), 4.26 (d, *J* = 6.2 Hz, 2H).

^{13}C NMR (150 MHz, DMSO-d_6) δ 162.52, 160.92, 158.51, 156.30, 147.24, 132.78, 125.33, 125.06, 124.64, 121.69, 120.33, 115.48, 105.35, 104.28, 102.10, 40.61.

3-((2-Hydroxy-5-bromophenyl)amino)coumarin (3b)

White solid; yield: 93%; mp:186℃-188℃.

^{1}H NMR (600 MHz, DMSO-d_6) δ 10.06 (s, 1H), 7.38 (d, *J* = 5.6 Hz, 1H), 7.31 – 7.26 (m, 2H), 7.24 – 7.21 (m, 2H), 6.81 (d, *J* = 8.5 Hz, 1H), 6.59 (d, *J* = 6.4 Hz, 1H), 6.46 (s, 1H), 4.28 (d, *J* = 6.4 Hz, 2H).

^{13}C NMR (150 MHz, DMSO-d_6) δ 158.48, 154.36, 147.30, 132.75, 130.43, 130.07, 127.05, 125.44, 125.10, 124.68, 121.62, 117.12, 115.53, 110.23, 104.29, 40.58.

3-((2-Hydroxy-5-chlorophenyl)amino)coumarin (3c)

White solid; yield: 91%; mp:189℃-190℃.

^{1}H NMR (400 MHz, DMSO-d_6) δ 10.02 (s, 1H), 7.37 (d, *J* = 7.3 Hz, 1H), 7.29 (d, *J* = 7.8 Hz, 1H), 7.25 – 7.17 (m, 2H), 7.15 (s, 1H), 7.10 (d, *J* = 11.3 Hz, 1H), 6.85 (d, *J* = 8.6 Hz, 1H), 6.45 (s, 1H), 4.28 (d, *J* = 6.4 Hz, 2H).

^{13}C NMR (101 MHz, DMSO-d_6) δ 158.46, 153.88, 147.29, 132.74, 127.49, 127.22, 126.49, 125.41, 125.08, 124.64, 122.55, 121.61, 116.54, 115.50, 104.28.

7-((2-Hydroxy-4-fluorophenyl)amino)-4-methylcoumarin (3d)

Yellow solid; yield: 88%; mp:215℃-217℃.

^{1}H NMR (600 MHz, DMSO-d_6) δ 10.31 (s, 1H), 7.42 (d, *J* = 8.8 Hz, 1H), 7.18 – 7.10 (m, 2H), 6.69 (d, *J* = 8.2 Hz, 1H), 6.63 (d, *J* = 8.7 Hz, 1H), 6.57 (d, *J* = 5.9 Hz, 1H), 6.38 (s, 1H), 5.90 (s, 1H), 4.21 (s, 2H), 2.29 (s, 3H).

^{13}C NMR (150 MHz, DMSO-d_6) δ 162.51, 160.73, 156.52, 155.57, 153.78, 152.34,

129.62, 129.55, 125.98, 121.06, 121.04, 108.86, 107.52, 105.15, 102.11, 40.39, 18.04.

7-((2-Hydroxy-5-bromophenyl)amino)-4-methylcoumarin (3e)

Brown solid; yield: 92%; mp:214°C-216°C.

^{1}H NMR (600 MHz, DMSO-*d6*) δ 10.10 (s, 1H), 7.43 (d, *J* = 8.6 Hz, 1H), 7.27 (s, 1H), 7.25 – 7.11 (m, 2H), 6.83 (d, *J* = 8.4 Hz, 1H), 6.63 (d, *J* = 8.7 Hz, 1H), 6.37 (s, 1H), 5.91 (s, 1H), 4.25 (s, 2H), 2.29 (s, 3H).

^{13}C NMR (150 MHz, DMSO-*d6*) δ 161.00, 155.86, 154.83, 154.09, 152.46, 130.78, 130.77, 128.04, 126.46, 117.54, 110.65, 110.41, 109.40, 108.07, 96.93, 50.30, 18.43.

7-((2-Hydroxy-5-chlorophenyl)amino)-4-methylcoumarin (3f)

Yellow solid; yield: 85%; mp:217°C-218°C.

^{1}H NMR (600 MHz, DMSO-*d6*) δ 7.38 (d, *J* = 8.9 Hz, 1H), 7.30 (s, 1H), 6.72 (s, 1H), 6.64 – 6.57 (m, 2H), 6.38 (s, 1H), 6.03 (d, *J* = 8.4 Hz, 1H), 5.85 (s, 1H), 4.07 (s, 2H), 2.28 (s, 3H).

^{13}C NMR (150 MHz, DMSO-*d6*) δ 160.88, 157.19, 155.75, 153.04, 148.79, 134.16, 127.49, 126.86, 126.06, 125.75, 119.69, 113.52, 108.25, 106.90, 96.52, 48.59, 18.05.

3-(N-(2-Hydroxy-4-fluorobenzyl)-N-isobutyrylamino)coumarin (4a)

Yellow solid; yield: 63%; mp:152°C-153°C.

^{1}H NMR (600 MHz, DMSO-*d6*) δ 7.39 (d, *J* = 8.5 Hz, 1H), 7.32 (d, *J* = 9.3 Hz, 1H), 7.28 (d, *J* = 9.5 Hz, 1H), 7.24 – 7.17 (m, 2H), 7.14 – 7.06 (m, 2H), 6.65 (d, *J* = 6.2 Hz, 1H), 4.24 (s, 2H), 1.95 – 1.88 (m, 1H), 1.40 – 1.21 (m, 2H), 0.97 (d, *J* = 6.7 Hz, 3H), 0.87 (t, *J* = 7.4 Hz, 3H).

^{13}C NMR (150 MHz, DMSO-*d6*) δ 171.02, 161.91, 160.30, 158.31, 149.15, 147.33, 132.52, 129.51, 125.56, 125.03, 124.68, 121.44, 115.52, 113.09, 110.54, 104.56, 40.31, 31.31, 28.68, 19.04, 11.17.

HRMS (ESI): m/z [M+H]+ calcd for $C_{20}H_{18}FNO_4$: 355.37, found: 356.13.

3-(N-(2-Hydroxy-4-fluorobenzyl)-N-(3-methylbutanoyl)amino)coumarin (4b)

White solid; yield: 55%; mp:114°C-116°C.

^{1}H NMR (600 MHz, DMSO-*d6*) δ 7.39 (d, *J* = 8.5 Hz, 1H), 7.32 (d, *J* = 9.3 Hz, 1H), 7.28 (d, *J* = 9.5 Hz, 1H), 7.24 – 7.17 (m, 2H), 7.14 – 7.06 (m, 2H), 6.65 (d, *J* = 6.2 Hz, 1H), 6.32 (s, 1H), 4.24 (s, 2H), 1.95 – 1.88 (m, 1H), 1.40 – 1.21 (m, 2H), 0.97 (d, *J* = 6.7 Hz, 3H), 0.87 (t, *J* = 7.4 Hz, 3H).

^{13}C NMR (150 MHz, DMSO-*d6*) δ 171.02, 161.91, 160.30, 158.31, 149.15, 147.33, 132.52, 129.51, 125.56, 125.03, 124.68, 121.44, 115.52, 113.09, 110.54, 104.56, 40.31, 31.31, 28.68, 19.04, 11.17.

HRMS (ESI): m/z [M+NA]+ calcd for $C_{21}H_{20}FNO_4$: 369.39, found: 370.14.

3-(N-(2-Hydroxy-4-fluorobenzyl)-N-(2-methylbut-2-enoyl)amino)coumarin (4c)

White solid; yield: 62%; mp:142℃-144℃.

^{1}H NMR (600 MHz, DMSO-d_6) δ 7.39 (d, J = 8.7 Hz, 1H), 7.33 – 7.26 (m, 2H), 7.25 – 7.18 (m, 2H), 7.14 – 7.08 (m, 2H), 6.60 (t, J = 6.3 Hz, 1H), 6.33 (s, 1H), 4.25 (s, 2H), 1.89 (s, 3H), 1.83 (d, J = 7.3 Hz, 3H).

^{13}C NMR (150 MHz, DMSO-d_6) δ 161.93, 160.32, 158.31, 149.49, 147.30, 140.65, 132.51, 129.45, 126.99, 126.54, 125.52, 125.02, 124.66, 121.47, 115.49, 112.93, 110.64, 104.50, 40.60, 14.54, 12.09.

HRMS (ESI): m/z [M+H]+ calcd for $C_{21}H_{18}FNO_4$: 367.38, found: 368.13.

3-(N-(2-Hydroxy-4-fluorobenzyl)-N-(thiophene-2-carbonyl)amino)coumarin (4d)

White solid; yield: 58%; mp:143℃-144℃.

^{1}H NMR (600 MHz, DMSO-d_6) δ 7.77 – 7.75 (m, 1H), 7.67 (d, J = 3.8 Hz, 1H), 7.43 (d, J = 6.4 Hz, 1H), 7.33 (d, J = 6.8 Hz, 1H), 7.31 (d, J = 5.8 Hz, 1H), 7.28 (d, J = 8.1 Hz, 1H), 7.24 – 7.21 (m, 1H), 7.19 (d, J = 6.0 Hz, 1H), 7.14 (d, J = 8.5 Hz, 1H), 6.67 (d, J = 6.3 Hz, 1H), 6.34 (s, 1H), 4.33 (d, J = 6.2 Hz, 2H).

^{13}C NMR (150 MHz, DMSO-d_6) δ 161.92, 160.07, 158.34, 148.86, 147.31, 136.14, 132.53, 131.25, 129.39, 128.33, 127.83, 126.59, 125.54, 125.06, 124.66, 121.44, 115.51, 113.27, 110.77, 104.56, 40.52.

HRMS (ESI): m/z [M+H]+ calcd for $C_{21}H_{14}FNO_4S$: 395.40, found: 396.07.

3-(N-(2-Hydroxy-5-bromobenzyl)-N-(5-chloro-2-(trifluoromethyl)benzoyl)amino)coumarin (4e)

White solid; yield: 56%; mp:177℃-179℃.

^{1}H NMR (600 MHz, DMSO-d_6) δ 7.97 (d, J = 8.5 Hz, 1H), 7.93 (d, J = 8.4 Hz, 1H), 7.63 (d, J = 6.1 Hz, 1H), 7.59 (s, 1H), 7.38 (d, J = 8.5 Hz, 1H), 7.29 – 7.25 (m, 2H), 7.24 – 7.21 (m, 1H), 7.18 (d, J = 6.1 Hz, 1H), 6.77 (d, J = 6.4 Hz, 1H), 6.37 (s, 1H), 4.37 (s, 2H).

^{13}C NMR (150 MHz, DMSO-d_6) δ 162.75, 158.28, 147.41, 147.35, 137.87, 133.29, 132.78, 132.45, 131.37, 130.96, 130.95, 130.73, 129.16, 129.13, 126.13, 125.91, 125.62, 124.99, 122.11, 121.32, 119.36, 115.50, 104.75, 40.71.

HRMS (ESI): m/z [M+H]+ calcd for $C_{24}H_{14}BrClF_3NO_4$: 552.73, found: 553.98.

3-(N-(2-Hydroxy-5-bromobenzyl)-N-(4-bromothiophene-2-carbonyl)amino)coumarin (4f)

White solid; yield: 69%; mp:198℃-200℃.

^{1}H NMR (600 MHz, DMSO-d_6) δ 7.89 (s, 1H), 7.58 – 7.55 (m, 2H), 7.33 – 7.30 (m, 2H), 7.28 (d, J = 8.2 Hz, 1H), 7.23 (d, J = 5.6 Hz, 1H), 7.19 (d, J = 6.1 Hz, 1H), 6.75 (d, J = 6.4 Hz, 1H), 6.36 (s, 1H), 4.36 (s, 2H).

^{13}C NMR (150 MHz, DMSO-d_6) δ 158.71, 158.31, 147.40, 147.37, 138.57, 133.36, 132.48, 131.08, 130.58, 128.50, 127.62, 125.62, 125.20, 125.08, 124.66, 121.37, 118.88, 115.53, 109.82, 104.59, 40.66.

HRMS (ESI): m/z [M+H]+ calcd for $C_{21}H_{13}Br_2NO_4S$: 535.21, found: 536.89.

3-(N-(2-Hydroxy-5-chlorobenzyl)-N-(2-(trifluoromethyl)benzoyl)amino)coumarin (4g)

White solid; yield: 49%; mp:146℃-147℃.

^{1}H NMR (600 MHz, DMSO-d_6) δ 8.27 (d, J = 5.5 Hz, 1H), 7.98 (d, J = 7.4 Hz, 1H), 7.92 – 7.86 (m, 2H), 7.49 (d, J = 5.9 Hz, 1H), 7.44 (s, 1H), 7.40 (d, J = 8.6 Hz, 1H), 7.30 – 7.25 (m, 2H), 7.24 – 7.20 (m, 1H), 7.17 (t, J = 7.4 Hz, 1H), 6.80 (d, J = 6.4 Hz, 1H), 6.34 (s, 1H), 4.37 (s, 2H).

^{13}C NMR (150 MHz, DMSO-d_6) δ 163.83, 158.01, 147.11, 146.63, 132.80, 132.75, 132.71, 132.21, 130.86, 130.79, 128.50, 128.10, 127.40, 126.86, 126.83, 125.35, 124.73, 124.36, 124.03, 123.98, 121.06, 115.24, 104.38, 40.36.

HRMS (ESI): m/z [M+H]+ calcd for $C_{24}H_{15}ClF_3NO_4$: 473.83, found: 475.06.

3-(N-(2-Hydroxy-5-chlorobenzyl)-N-(2,4-dichloro-5-fluorobenzoyl)amino)coumarin (4h)

White solid; yield: 73%; mp:168℃-170℃.

^{1}H NMR (600 MHz, DMSO-d_6) δ 8.25 (d, J = 9.4 Hz, 1H), 7.49 – 7.45 (m, 2H), 7.42 (d, J = 8.3 Hz, 1H), 7.28 – 7.24 (m, 2H), 7.23 – 7.20 (m, 1H), 7.17 (d, J = 6.0 Hz, 1H), 6.69 (d, J = 6.4 Hz, 1H), 6.40 (s, 1H), 4.39 (s, 2H).

^{13}C NMR (150 MHz, DMSO-d_6) δ 161.32, 158.27, 156.63, 154.98, 147.28, 133.02, 132.76, 132.39, 130.87, 129.11, 129.08, 128.30, 128.26, 125.58, 124.96, 124.66, 124.58, 121.30, 120.08, 119.91, 115.46, 104.70, 41.02.

HRMS (ESI): m/z [M+H]+ calcd for $C_{23}H_{13}Cl_3FNO_4$: 492.71, found: 494.98.

3-(N-(2-Hydroxy-5-chlorobenzyl)-N-isobutyrylamino)coumarin (4i):

Light yellow solid; yield: 77%; mp:152℃-154℃.

^{1}H NMR (600 MHz, DMSO-d_6) δ 7.38 (d, J = 3.3 Hz, 1H), 7.33 (d, J = 5.9 Hz, 1H), 7.29 (d, J = 8.1 Hz, 1H), 7.24 (d, J = 7.3 Hz, 1H), 7.21 – 7.18 (m, 2H), 6.75 (d, J = 6.4 Hz, 1H), 6.32 (s, 1H), 4.26 (s, 2H), 2.17 – 2.11 (m, 1H), 1.00 (d, J = 6.6 Hz, 6H).

^{13}C NMR (150 MHz, DMSO-d_6) δ 171.03, 158.31, 147.40, 147.23, 132.74, 132.52, 130.36, 128.09, 127.56, 125.66, 125.08, 124.72, 124.69, 124.46, 121.40, 115.57,

104.57, 42.15, 25.17, 22.16.

HRMS (ESI): m/z [M+H]+ calcd for $C_{20}H_{18}ClNO_4$: 371.82, found: 373.09.

3-(N-(2-Hydroxy-5-chlorobenzyl)-N-(thiophene-2-carbonyl)amino)coumarin (4j):

White solid; yield: 67%; mp:190°C-192°C.

^{1}H NMR (600 MHz, DMSO-d_6) δ 8.70 (d, J = 4.0 Hz, 1H), 7.75 (dd, J = 5.2, 2.9 Hz, 1H), 7.67 (d, J = 5.1 Hz, 1H), 7.42 (d, J = 8.1 Hz, 2H), 7.37 (d, J = 8.2 Hz, 1H), 7.31 (d, J = 6.1 Hz, 1H), 7.27 (d, J = 8.1 Hz, 1H), 7.24 – 7.20 (m, 1H), 7.18 (t, J = 7.4 Hz, 1H), 6.74 (d, J = 6.4 Hz, 1H), 6.35 (s, 1H), 4.34 (s, 2H).

^{13}C NMR (150 MHz, DMSO-d_6) δ 159.91, 158.01, 147.08, 146.79, 135.79, 132.65, 132.21, 130.98, 130.23, 128.00, 127.78, 127.53, 127.30, 125.31, 124.80, 124.47, 124.35, 121.08, 115.22, 104.29, 40.42.

HRMS (ESI): m/z [M+H]+ calcd for $C_{21}H_{14}ClNO_4S$: 411.86, found: 413.03.

3-(N-(2-Hydroxy-5-chlorobenzyl)-N-(4-bromothiophene-2-carbonyl)amino)coumarin (4k)

White solid; yield: 75%; mp:171°C-172°C.

^{1}H NMR (600 MHz, DMSO-d_6) δ 8.09 (s, 1H), 7.45 – 7.42 (m, 2H), 7.40 (d, J = 9.2 Hz, 1H), 7.31 (d, J = 5.9 Hz, 1H), 7.27 (d, J = 8.2 Hz, 1H), 7.22 (d, J = 8.1 Hz, 1H), 7.18 (d, J = 6.0 Hz, 1H), 6.70 (d, J = 6.4 Hz, 1H), 6.39 (s, 1H), 4.37 (s, 2H).

^{13}C NMR (150 MHz, DMSO-d_6) δ 158.65, 158.28, 147.33, 146.81, 136.93, 132.98, 132.95, 132.75, 132.44, 130.78, 128.16, 127.99, 125.59, 125.07, 124.68, 124.63, 121.36, 115.50, 110.31, 104.60, 40.76.

HRMS (ESI): m/z [M+H]+ calcd for $C_{21}H_{13}BrClNO_4S$: 490.75, found: 491.94.

7-(N-(2-Hydroxy-4-fluorobenzyl)-N-(2-methylbut-2-enoyl)amino)-4-methylcoumarin (4l):

Yellow solid; yield: 72%; mp:116°C-118°C.

^{1}H NMR (600 MHz, DMSO-d_6) δ 7.43 (d, J = 8.8 Hz, 1H), 7.38 (d, J = 6.4 Hz, 1H), 7.19 – 7.12 (m, 2H), 6.60 (d, J = 6.4 Hz, 1H), 6.34 (s, 1H), 5.93 (d, J = 1.3 Hz, 1H), 4.22 (s, 2H), 2.30 (s, 3H), 1.87 (s, 3H), 1.82 (d, J = 7.1 Hz, 3H).

^{13}C NMR (150 MHz, DMSO-d_6) δ 165.21, 160.62, 155.48, 153.72, 151.92, 149.62, 140.57, 129.61, 129.55, 127.21, 127.19, 126.95, 126.06, 112.89, 112.75, 110.51, 109.18, 107.84, 40.72, 18.02, 14.51, 12.07.

HRMS (ESI): m/z [M+H]+ calcd for $C_{22}H_{20}FNO_4$: 381.40, found: 382.14.

7-(N-(2-Hydroxy-4-fluorobenzyl)-N-(2-(trifluoromethyl)benzoyl)amino)-4-methylcoumarin (4m)

Brown solid; yield: 48%; mp:129°C-130°C.

^{1}H NMR (600 MHz, DMSO-*d$_6$*) δ 7.99 (d, *J* = 9.1 Hz, 1H), 7.86 (d, *J* = 9.5 Hz, 1H), 7.81 – 7.75 (m, 2H), 7.66 – 7.61 (m, 2H), 7.44 – 7.40 (m, 2H), 7.37 – 7.31 (m, 2H), 7.26 – 7.20 (m, 2H), 6.81 (d, *J* = 6.4 Hz, 1H), 6.25 (s, 1H), 2.20 (s, 3H).

^{13}C NMR (150 MHz, DMSO-*d$_6$*) δ 167.18, 163.59, 162.60, 152.97, 152.68, 149.50, 134.20, 133.25, 132.76, 131.54, 128.93, 127.08, 126.86, 126.03, 125.28, 124.27, 123.05, 118.51, 116.00, 114.86, 114.07, 110.34, 110.18, 47.37, 18.12.

HRMS (ESI): m/z [M+H]+ calcd for $C_{25}H_{17}F_4NO_4$: 471.41, found: 472.11.

7-(N-(2-Hydroxy-4-fluorobenzyl)-N-(5-chloro-2-(trifluoromethyl)benzoyl)amino)-4-methylcoumarin (4n)

Light yellow solid; yield: 58%; mp:177°C-179°C.

^{1}H NMR (600 MHz, DMSO-*d$_6$*) δ 7.97 (s, 1H), 7.93 (d, *J* = 8.0 Hz, 1H), 7.46 (d, *J* = 8.6 Hz, 1H), 7.41 – 7.37 (m, 2H), 7.24 (d, *J* = 5.8 Hz, 1H), 7.15 (s, 1H), 6.60 (d, *J* = 6.5 Hz, 1H), 6.35 (s, 1H), 5.92 (d, *J* = 1.2 Hz, 1H), 4.34 (s, 2H), 2.29 (s, 3H).

^{13}C NMR (150 MHz, DMSO-*d$_6$*) δ 162.45, 160.57, 155.44, 153.64, 151.86, 148.82, 137.77, 132.73, 130.94, 130.54, 130.18, 130.12, 129.13, 127.31, 126.23, 126.04, 123.89, 122.08, 113.69, 110.39, 109.24, 107.91, 96.78, 40.86, 18.01.

HRMS (ESI): m/z [M+H]+ calcd for $C_{25}H_{16}ClF_4NO_4$: 505.85, found: 507.07.

7-(N-(2-Hydroxy-5-bromobenzyl)-N-(2-bromobutanoyl)amino)-4-methylcoumarin (4o)

Light yellow solid; yield: 67%; mp:120°C-121°C.

^{1}H NMR (600 MHz, DMSO-*d$_6$*) δ 7.54 (s, 1H), 7.46 (d, *J* = 8.8 Hz, 1H), 7.22 – 7.17 (m, 2H), 6.62 (d, *J* = 11.1 Hz, 1H), 6.35 (d, *J* = 2.4 Hz, 1H), 5.95 (s, 1H), 5.02 – 4.97 (m, 1H), 4.28 (s, 2H), 2.30 (s, 3H), 2.21 – 2.01 (m, 2H), 1.05 (t, *J* = 7.3 Hz, 3H).

^{13}C NMR (150 MHz, DMSO-*d$_6$*) δ 167.63, 160.55, 155.41, 153.72, 151.63, 147.32, 133.65, 131.26, 130.91, 126.26, 125.05, 124.60, 119.08, 117.22, 109.51, 108.17, 58.84, 40.47, 27.41, 18.03, 10.16.

HRMS (ESI): m/z [M+H]+ calcd for $C_{21}H_{20}BrNO_4$: 509.19, found: 511.08.

7-(N-(2-Hydroxy-5-bromobenzyl)-N-isobutyrylamino)-4-methylcoumarin (4p):

White solid; yield: 76%; mp:161°C-162°C.

^{1}H NMR (600 MHz, DMSO-*d$_6$*) δ 7.52 (d, *J* = 8.5 Hz, 1H), 7.50 (s, 1H), 7.46 (d, *J* = 8.8 Hz, 1H), 7.17 (d, *J* = 6.0 Hz, 1H), 7.14 (d, *J* = 8.4 Hz, 1H), 6.34 (s, 1H), 5.94 (s, 1H), 4.24 (s, 2H), 2.93 – 2.86 (m, 1H), 2.30 (s, 3H), 1.25 (d, *J* = 7.0 Hz, 6H).

^{13}C NMR (150 MHz, DMSO-*d$_6$*) δ 174.68, 160.58, 155.44, 153.74, 151.72, 147.91,

133.72, 131.03, 130.67, 129.91, 126.25, 125.02, 118.43, 110.30, 109.44, 108.10, 96.82, 40.64, 33.37, 18.66, 18.05.

HRMS (ESI): m/z [M+NA]+ calcd for $C_{21}H_{20}BrNO_4$: 430.30, found: 452.04.

7-(N-(2-Hydroxy-5-bromobenzyl)-N-(2-methylbut-2-enoyl)amino)-4-methylcoumarin (4q)

White solid; yield: 78%; mp: 154–155 °C.

^{1}H NMR (600 MHz, DMSO-d_6) δ 7.49 (s, 1H), 7.44 (d, J = 8.7 Hz, 1H), 7.19 – 7.13 (m, 2H), 7.10 (d, J = 5.6 Hz, 1H), 6.62 – 6.58 (m, 1H), 6.34 (s, 1H), 5.94 (s, 1H), 4.25 (s, 2H), 1.87 (s, 3H), 1.83 (d, J = 5.8 Hz, 3H).

^{13}C NMR (151 MHz, DMSO-d_6) δ 165.77, 161.04, 155.91, 154.17, 152.17, 148.64, 141.04, 134.32, 131.42, 131.16, 127.43, 126.64, 125.60, 118.73, 110.81, 109.83, 108.48, 97.25, 41.20, 18.49, 14.98, 12.54.

HRMS (ESI): m/z [M+H]+ calcd for $C_{22}H_{20}BrNO_4$: 442.31, found: 443.06.

7-(N-(2-Hydroxy-5-chlorobenzyl)-N-(2-bromobutanoyl)amino)-4-methylcoumarin (4r)

Yellow solid; yield: 68%; mp:156°C-157°C.

^{1}H NMR (600 MHz, DMSO-d_6) δ 7.46 (d, J = 8.7 Hz, 1H), 7.43 (d, J = 8.5 Hz, 1H), 7.41 (s, 1H), 7.27 (d, J = 8.6 Hz, 1H), 6.62 (d, J = 6.4 Hz, 1H), 6.35 (s, 1H), 5.95 (d, J = 1.2 Hz, 1H), 5.03 – 4.95 (m, 1H), 4.28 (s, 2H), 2.30 (s, 3H), 2.23 – 2.01 (m, 2H), 1.05 (t, J = 7.3 Hz, 3H).

^{13}C NMR (150 MHz, DMSO-d_6) δ 167.70, 160.55, 155.41, 153.71, 151.63, 146.82, 133.33, 130.85, 128.29, 127.95, 126.25, 124.52, 124.26, 122.27, 109.51, 108.17, 58.83, 40.53, 27.42, 18.03, 10.16.

HRMS (ESI): m/z [M+NA]+ calcd for $C_{21}H_{20}BrClNO_4$: 464.74, found: 465.13.

7-(N-(2-Hydroxy-5-chlorobenzyl)-N-isobutyrylamino)-4-methylcoumarin (4s)

White solid; yield: 66%; mp:149°C-151°C.

^{1}H NMR (600 MHz, DMSO-d_6) δ 7.46 (d, J = 8.8 Hz, 1H), 7.39 (d, J = 5.9 Hz, 1H), 7.36 (s, 1H), 7.21 – 7.16 (m, 2H), 6.34 (s, 1H), 5.94 (s, 1H), 4.23 (d, J = 6.0 Hz, 2H), 2.93 – 2.87 (m, 1H), 2.30 (s, 3H), 1.25 (d, J = 7.0 Hz, 6H).

^{13}C NMR (150 MHz, DMSO-d_6) δ 174.73, 160.57, 155.43, 153.73, 151.72, 147.40, 133.36, 130.21, 128.07, 127.72, 127.17, 126.24, 124.63, 110.30, 109.44, 108.10, 96.83, 40.70, 33.36, 18.66, 18.04.

HRMS (ESI): m/z [M+H]+ calcd for $C_{21}H_{20}ClNO_4$: 385.84, found: 386.11.

7-(N-(2-Hydroxy-5-bromobenzyl)-N-(2-methylbut-2-enoyl)amino)-4-methylcoumarin (4t)

White solid; yield: 73%; mp:151°C-152°C.

^{1}H NMR (600 MHz, DMSO-d_6) δ 7.45 (d, J = 8.7 Hz, 1H), 7.39 (d, J = 5.9 Hz, 1H), 7.36 (s, 1H), 7.23 (d, J = 8.5 Hz, 1H), 7.17 (s, 1H), 6.62 – 6.59 (m, 1H), 6.35 (d, J = 2.3 Hz, 1H), 5.94 (s, 1H), 4.25 (d, J = 6.0 Hz, 2H), 2.30 (s, 3H), 1.88 (s, 3H), 1.83 (d, J = 5.8 Hz, 3H). ^{13}C NMR (150 MHz, DMSO-d_6) δ 165.38, 160.58, 155.45, 153.71, 151.72, 147.68, 140.56, 133.51, 130.07, 128.01, 127.74, 126.98, 126.18, 124.77, 110.35, 109.37, 108.03, 96.80, 40.81, 18.03, 14.52, 12.09.

HRMS (ESI): m/z [M+H]+ calcd for $C_{22}H_{20}ClNO_4$: 397.85, found: 399.11.

Spectra of ^{1}H NMR, ^{13}C NMR

^{1}H NMR of compound 1a.

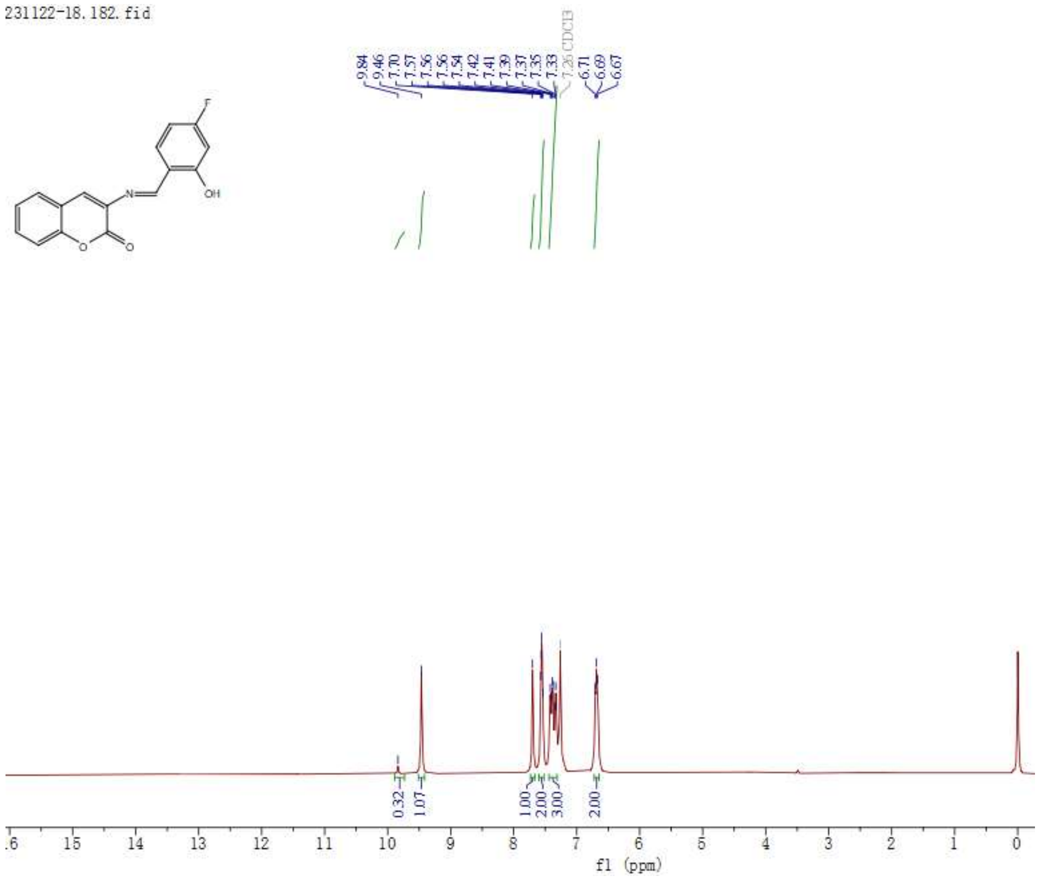

^{13}C NMR of compound 1a.

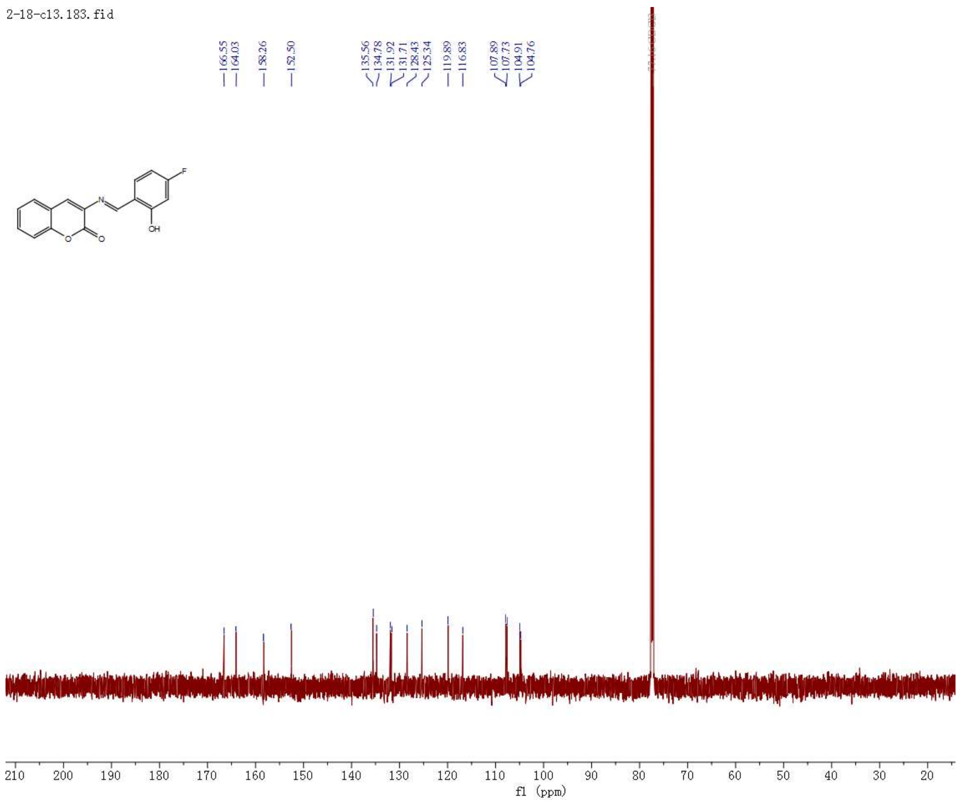

^{1}H NMR of compound 1b.

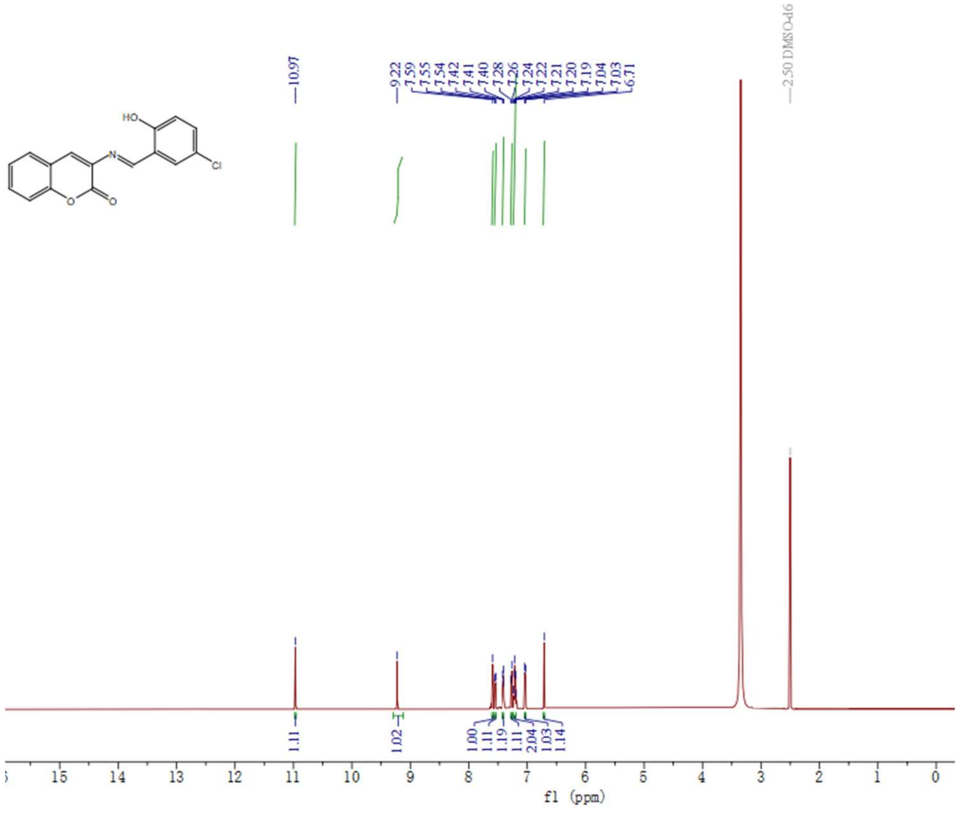

^{13}C NMR of compound 1b.

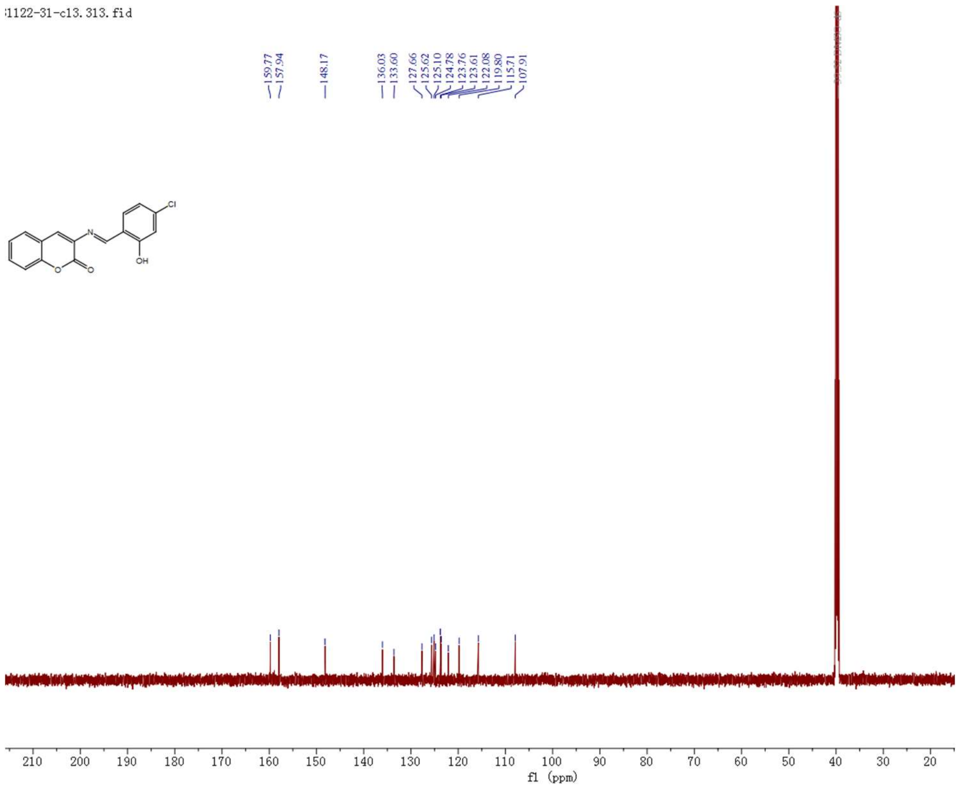

^{1}H NMR of compound 1c.

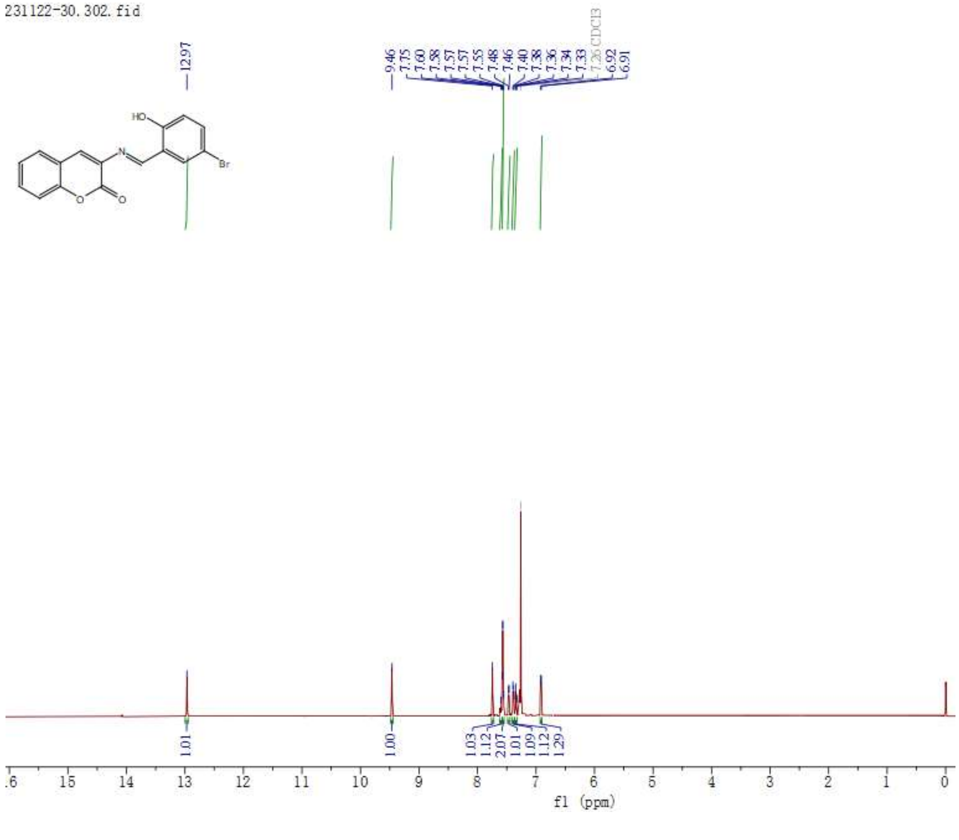

^{13}C NMR of compound 1c.

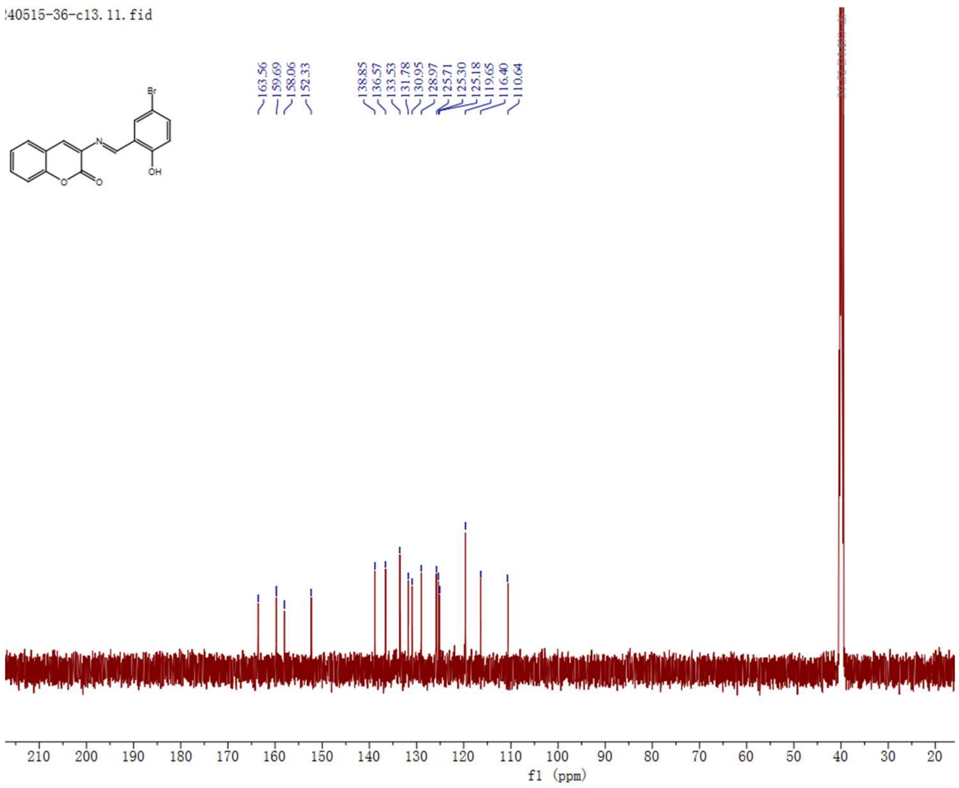

^{1}H NMR of compound 2a.

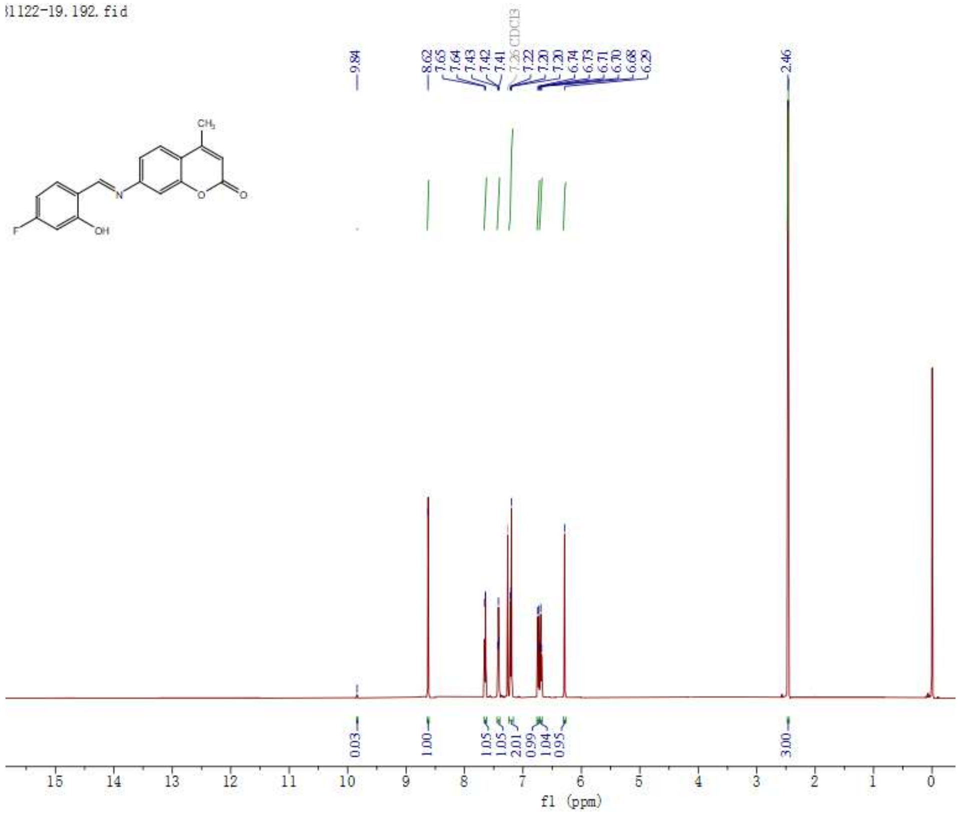

^{13}C NMR of compound 2a.

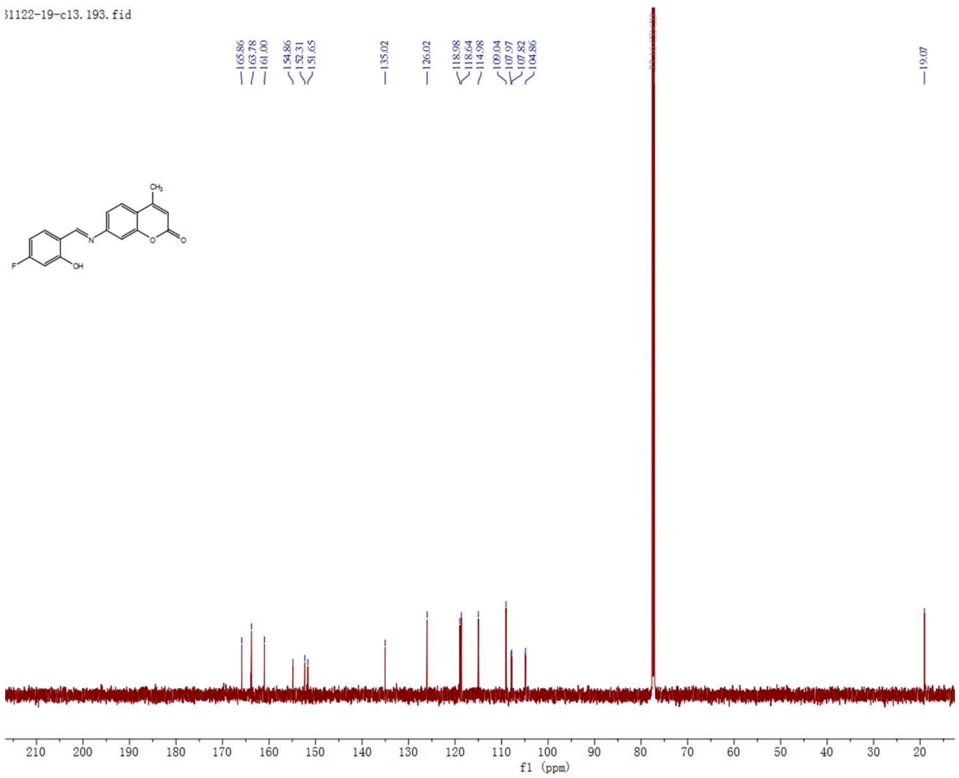

^{1}H NMR of compound 2b.

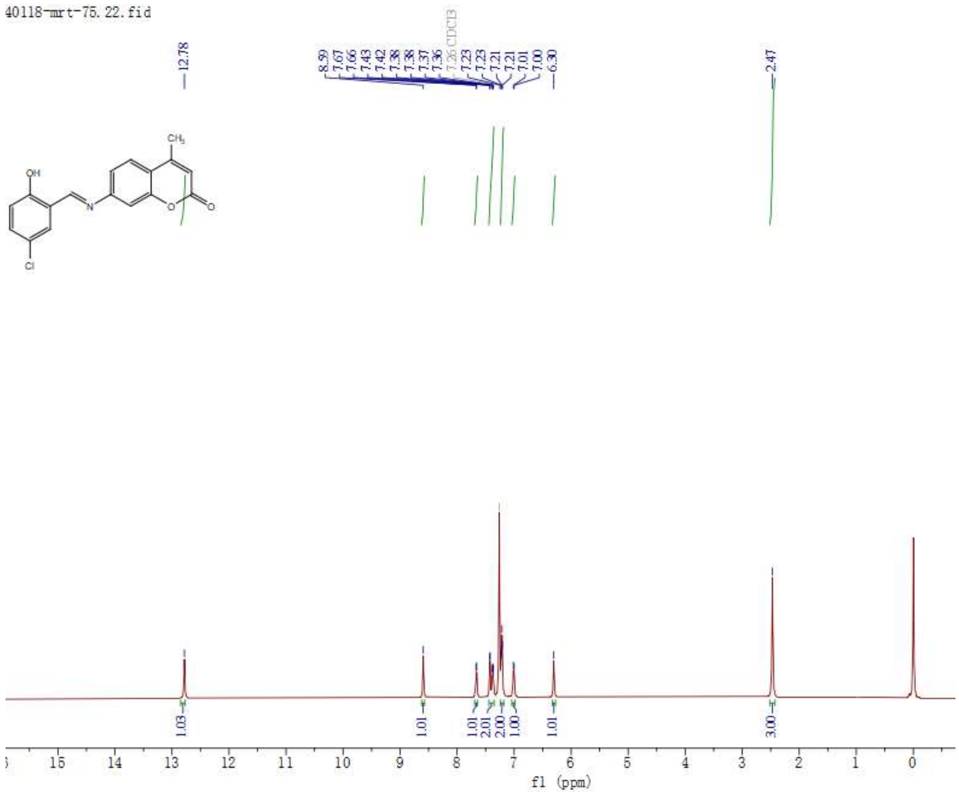

^{13}C NMR of compound 2b.

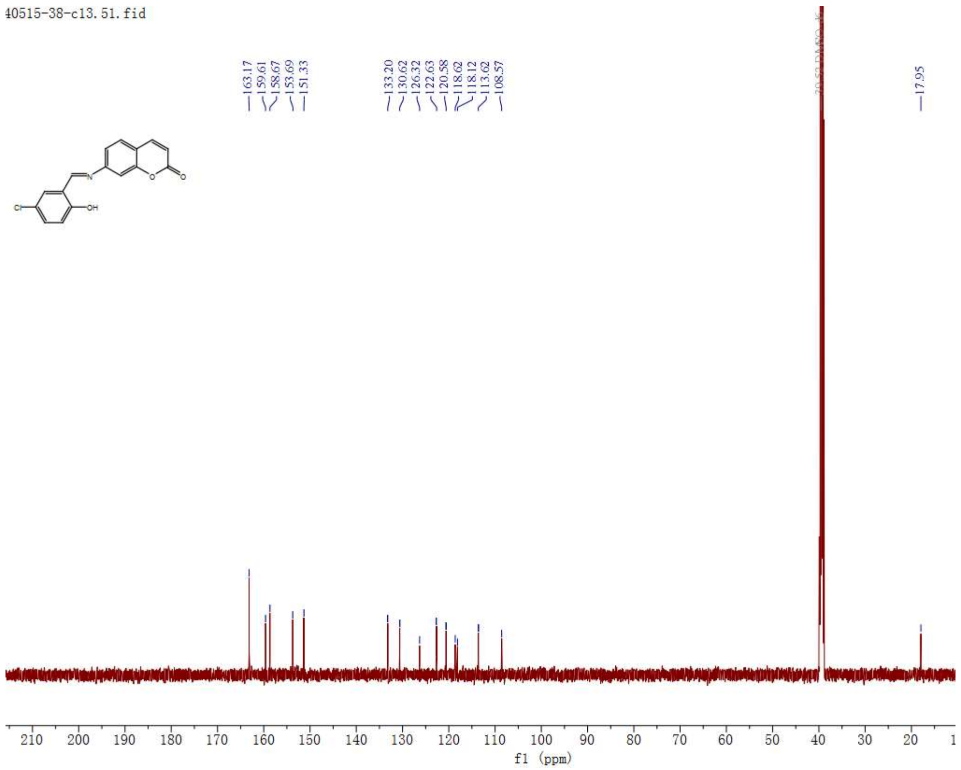

^{1}H NMR of compound 2c.

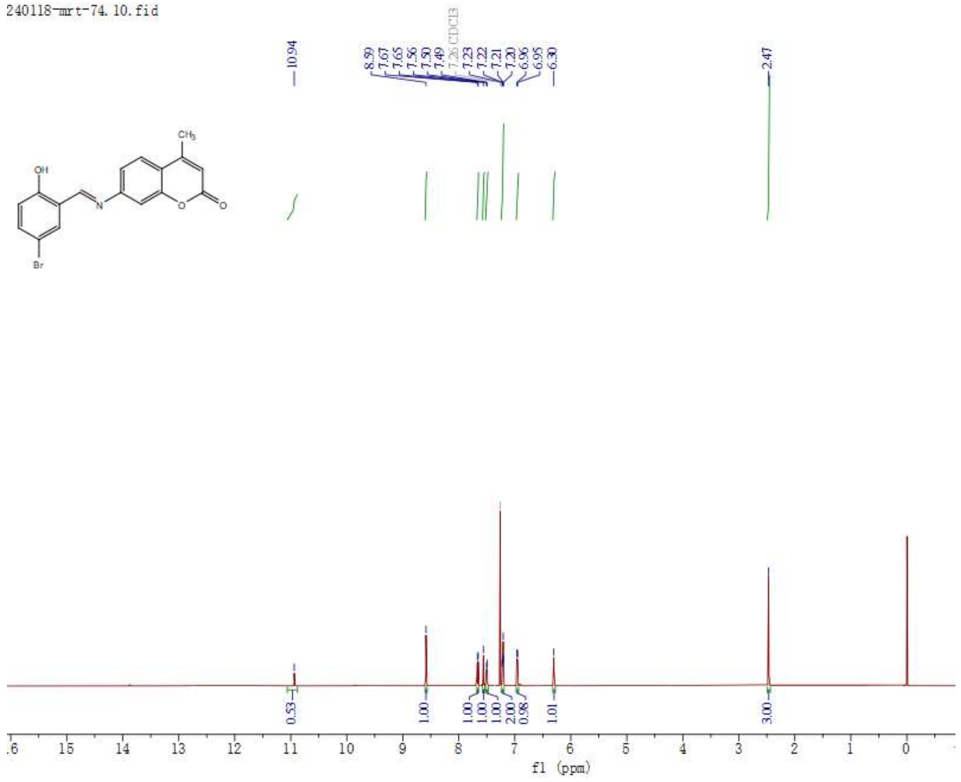

^{13}C NMR of compound 2c.

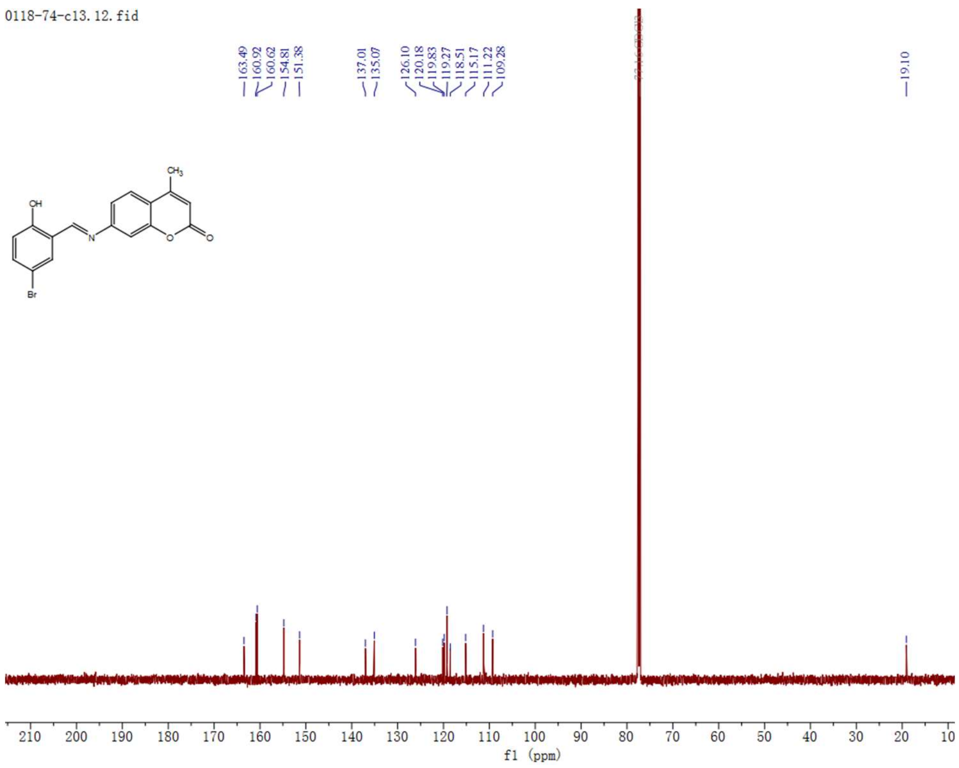

^{1}H NMR of compound 3a.

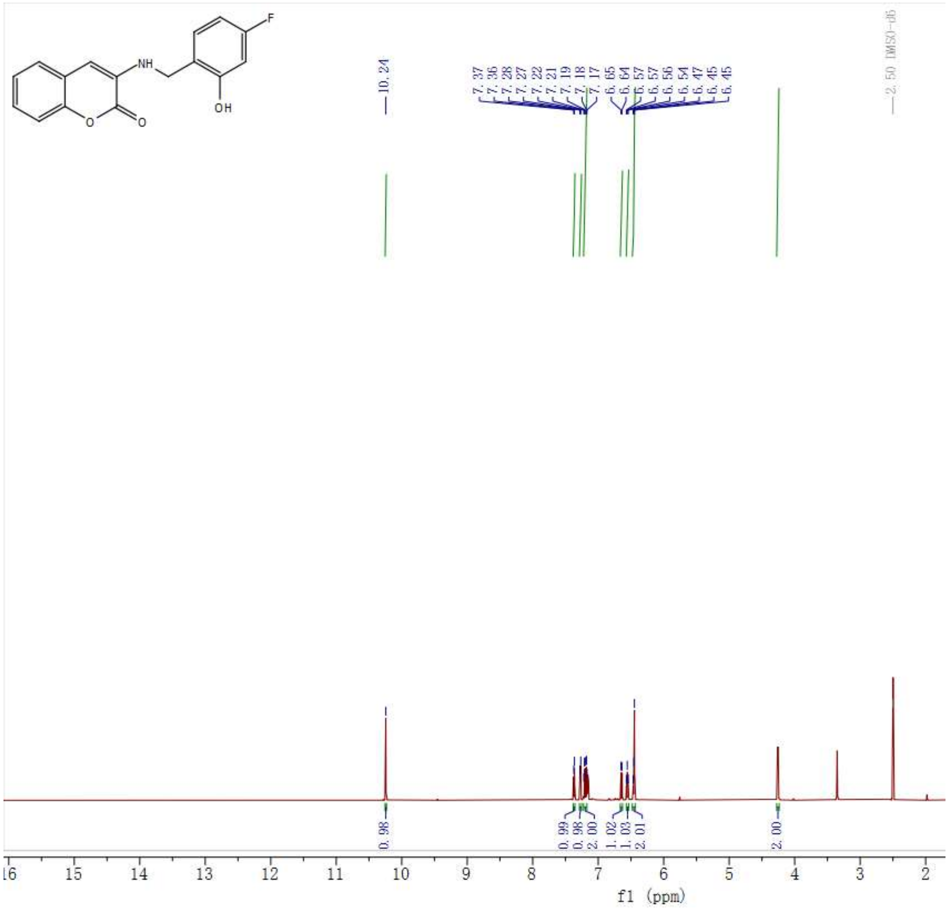

^{13}C NMR of compound 3a.

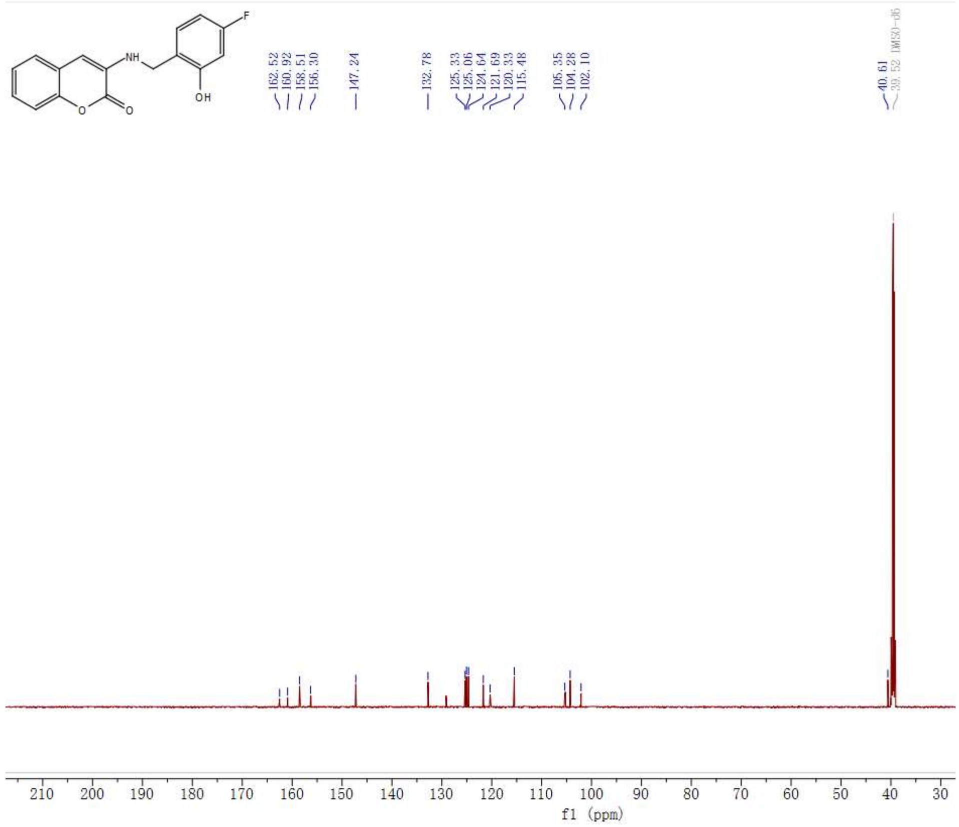

^{1}H NMR of compound 3b.

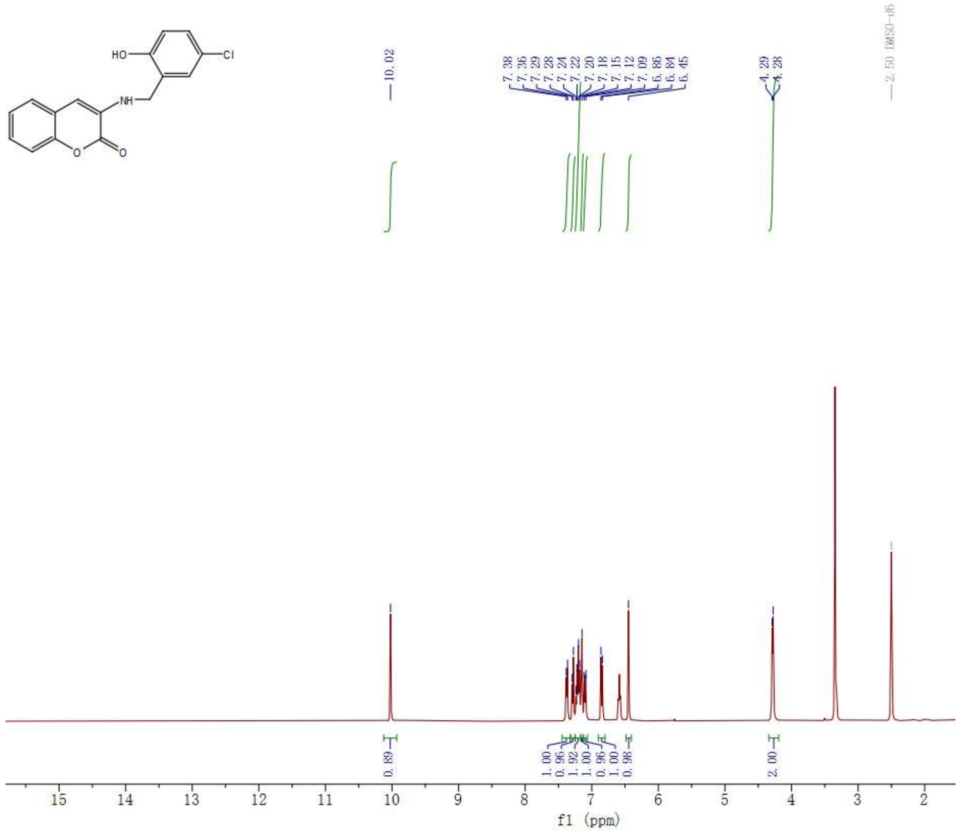

^{13}C NMR of compound 3b.

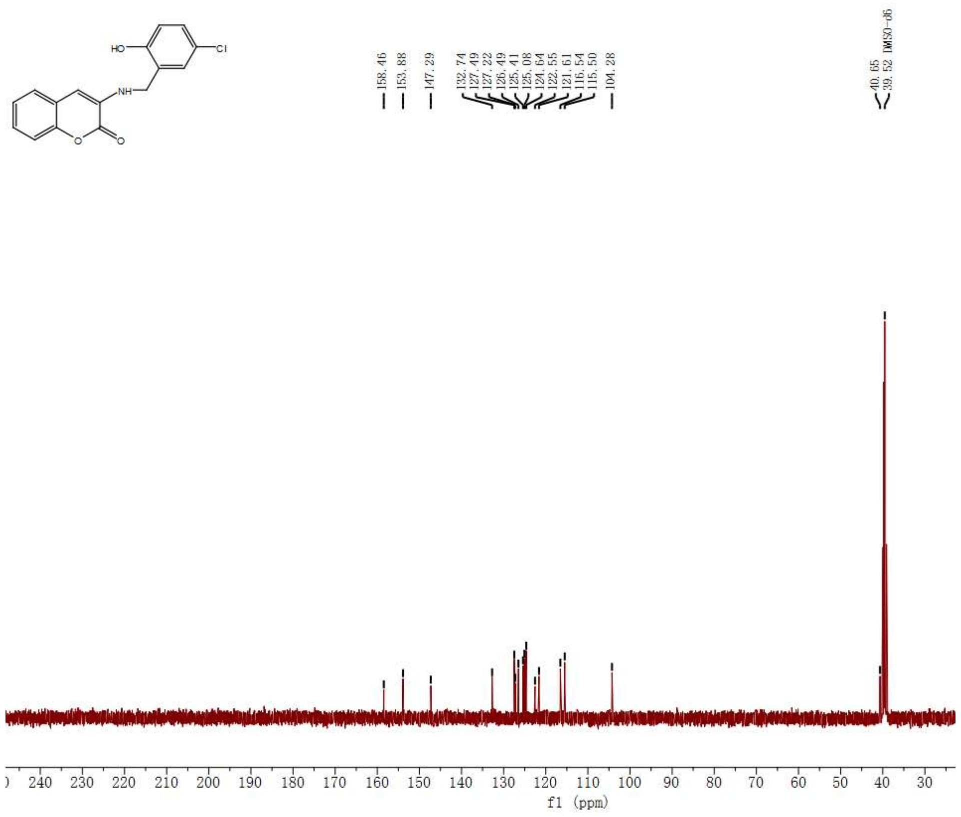

^{1}H NMR of compound 3c.

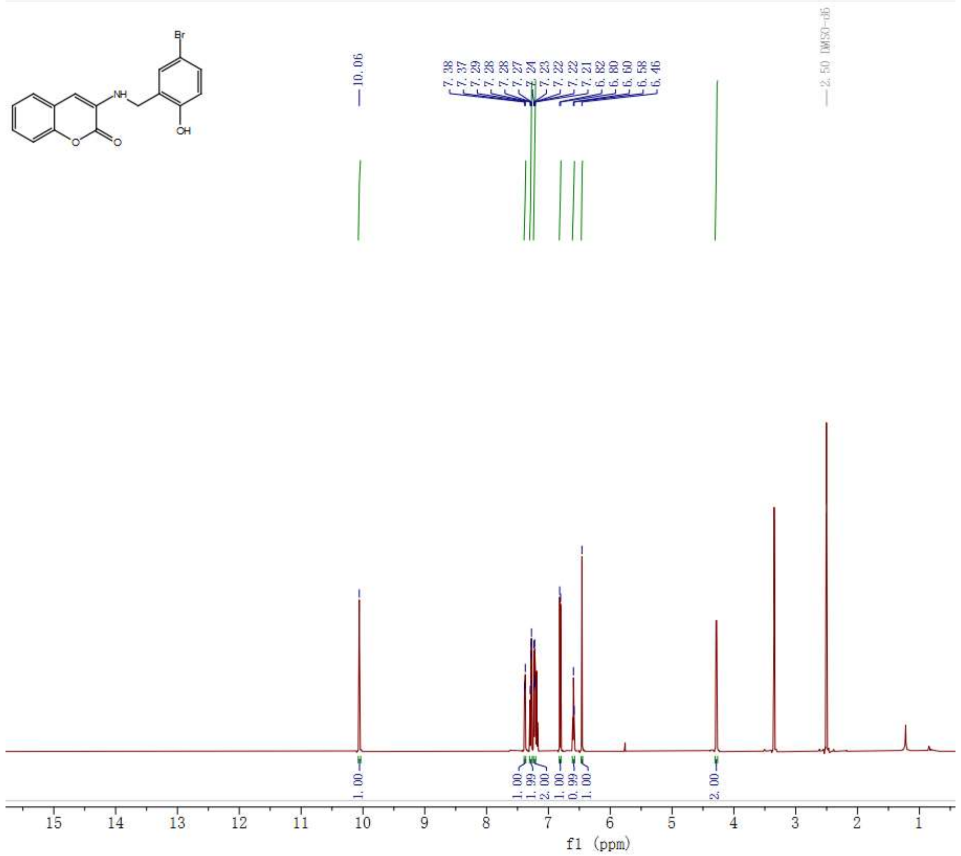

^{13}C NMR of compound 3c.

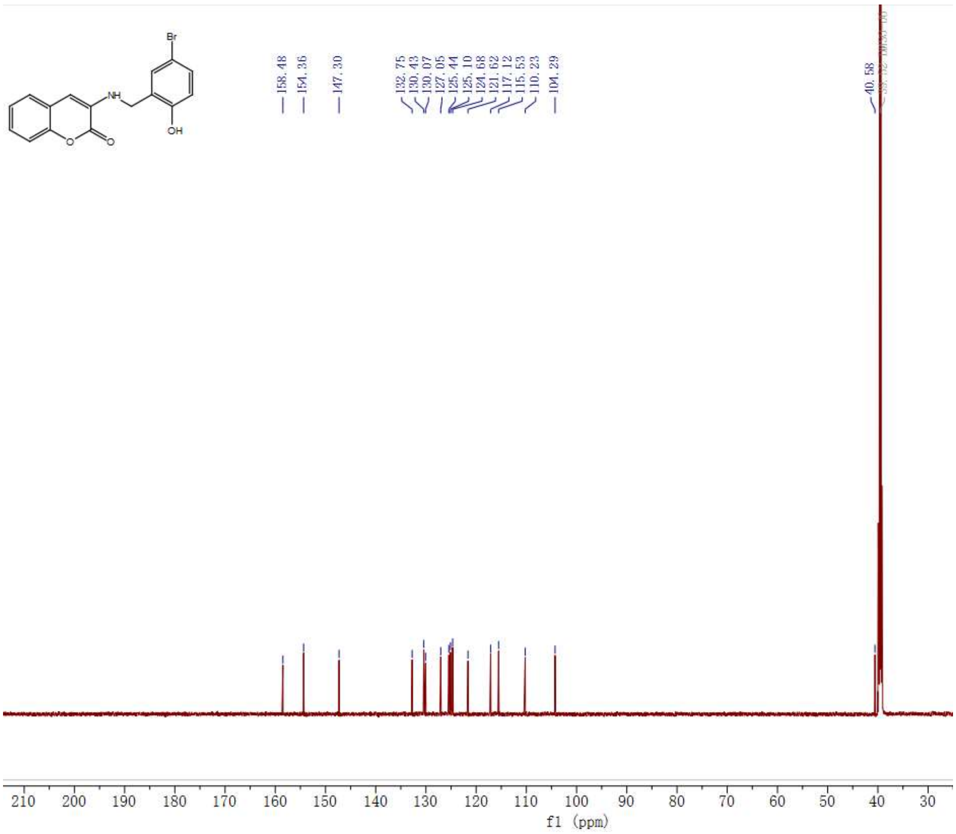

^{1}H NMR of compound 3d.

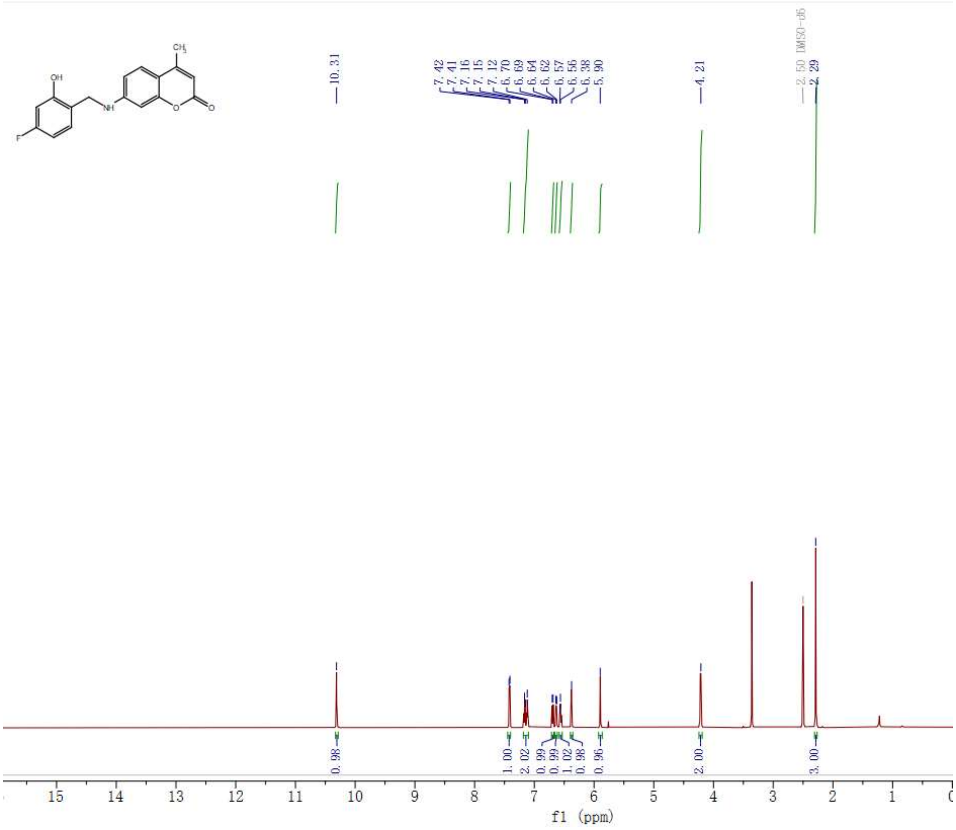

^{13}C NMR of compound 3d.

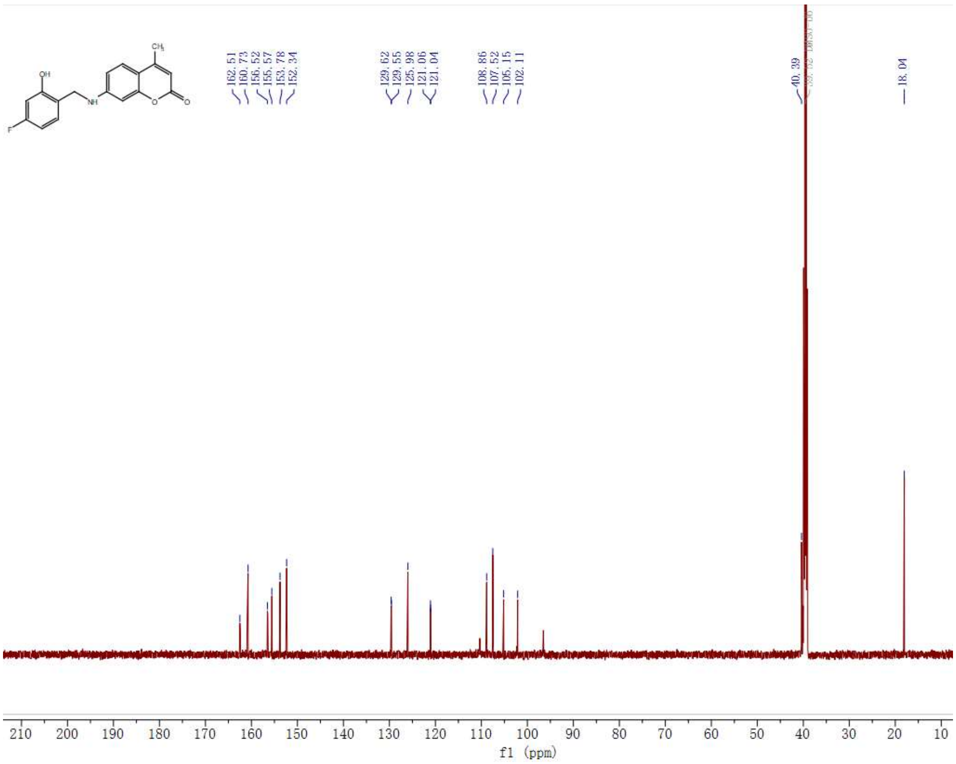

^{1}H NMR of compound 3e.

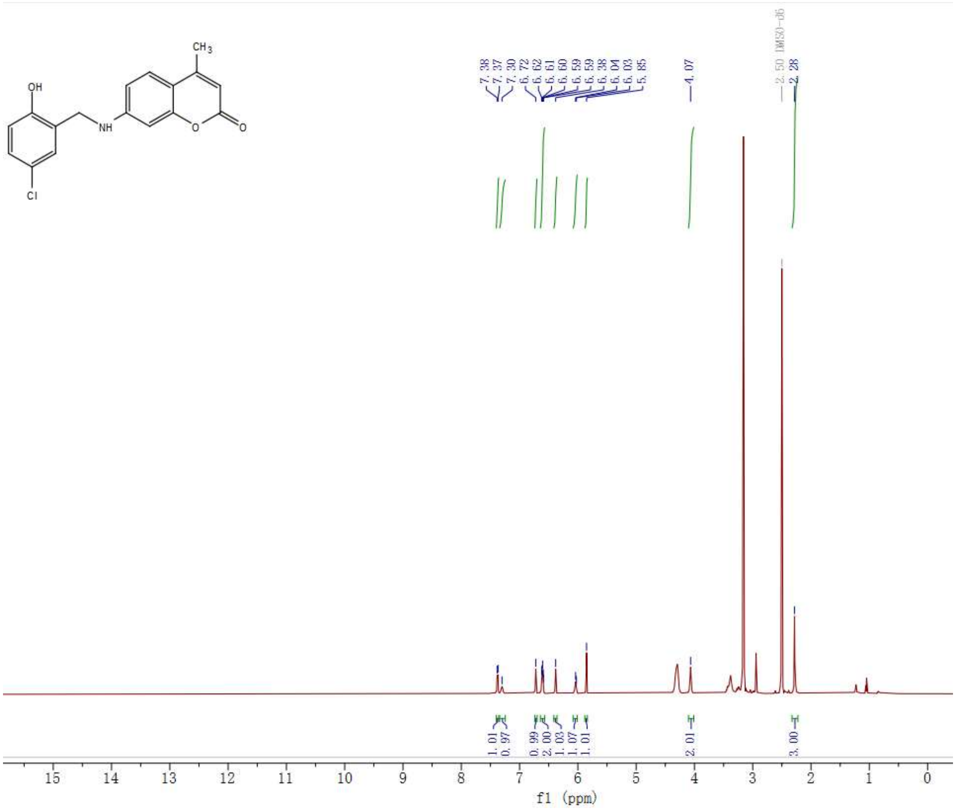

^{13}C NMR of compound 3e.

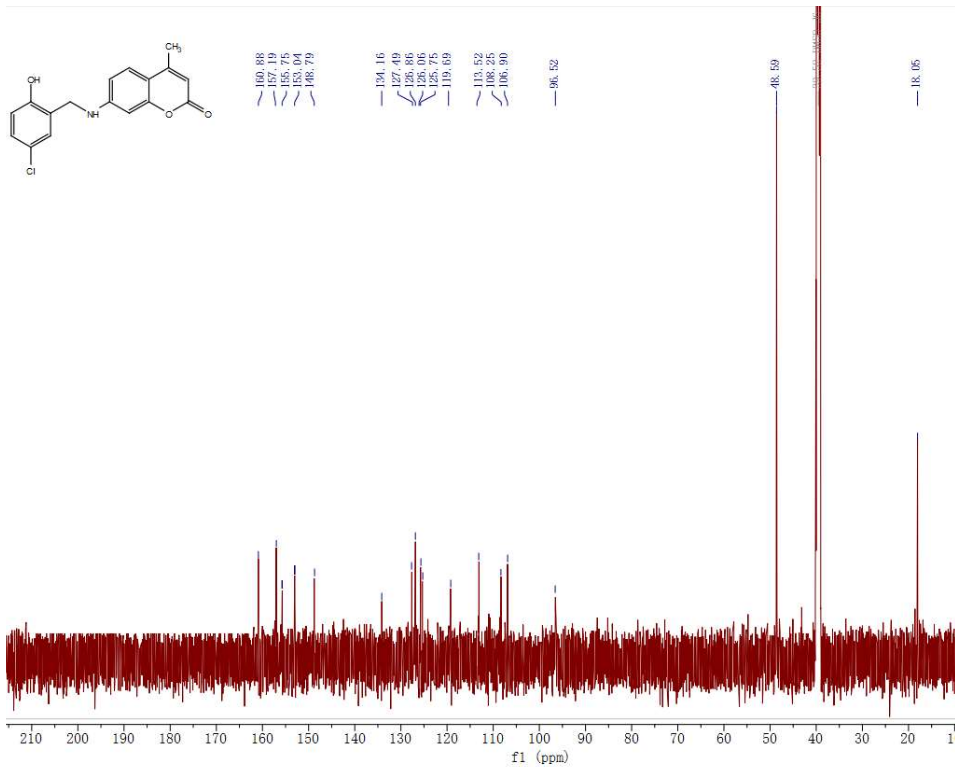

^{1}H NMR of compound 3f.

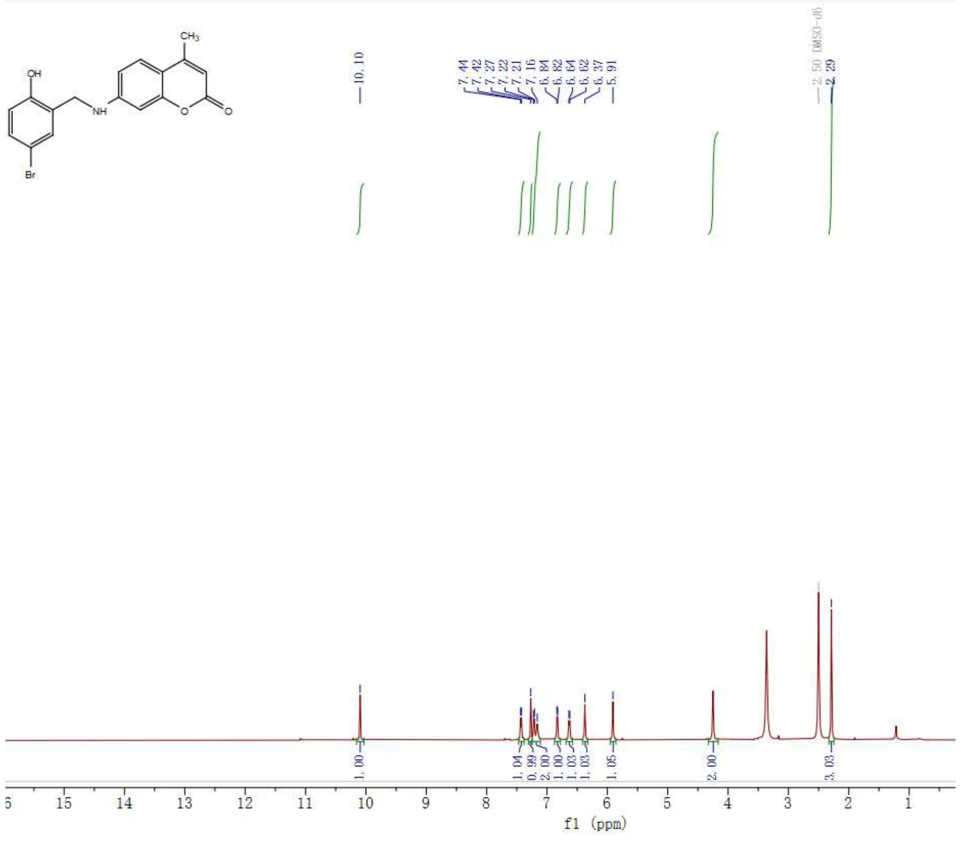

^{13}C NMR of compound 3f.

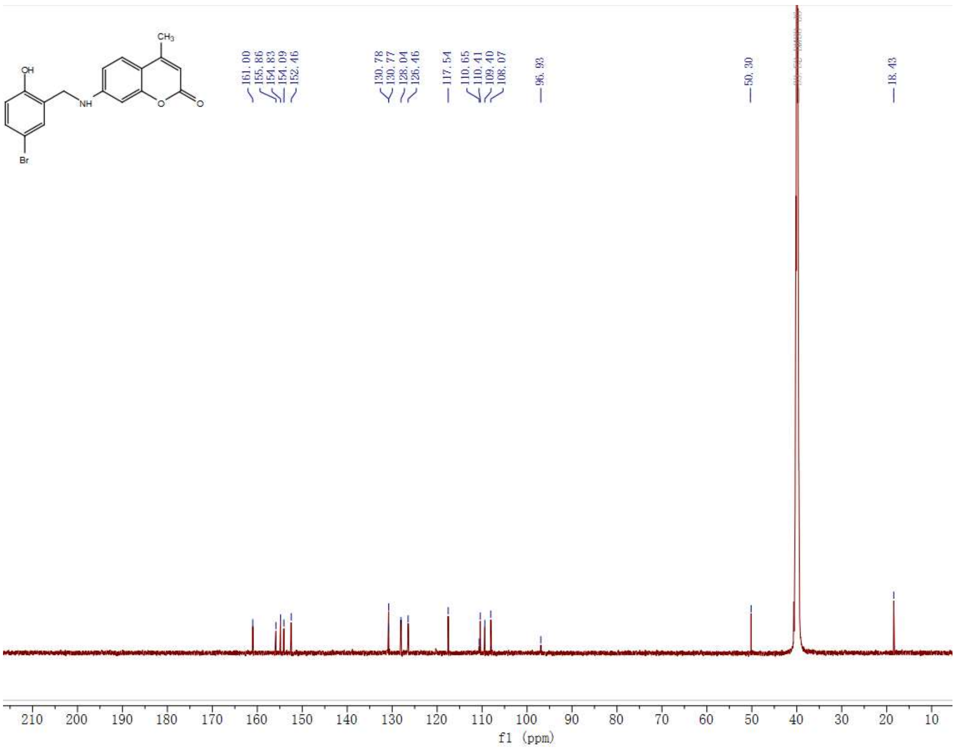

^{1}H NMR of compound 4a.

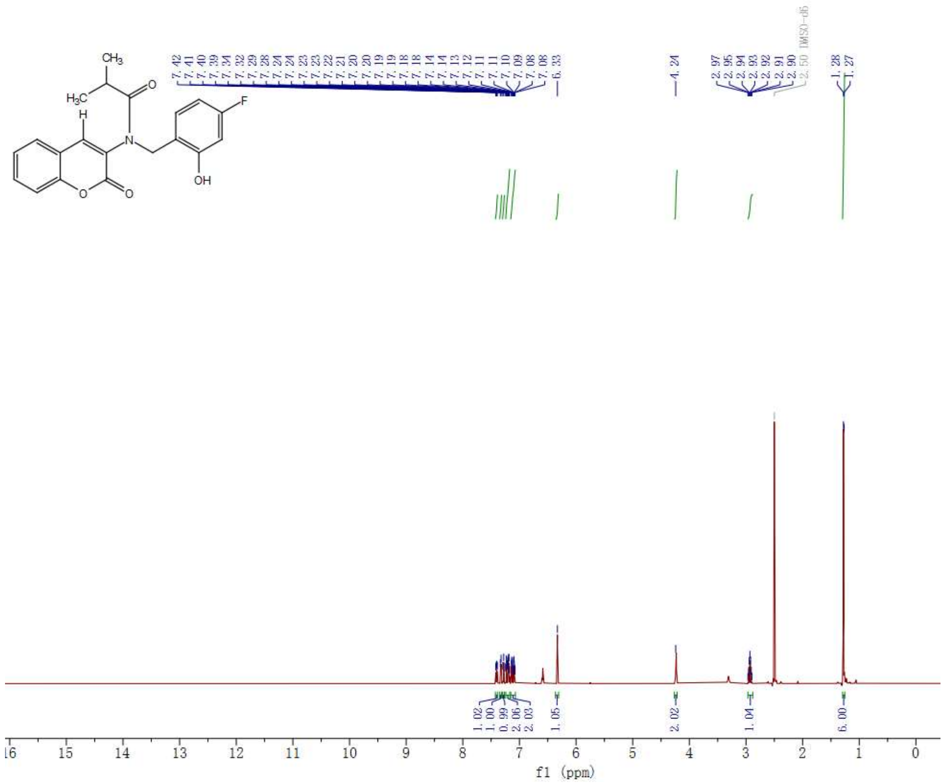

^{13}C NMR of compound 4a.

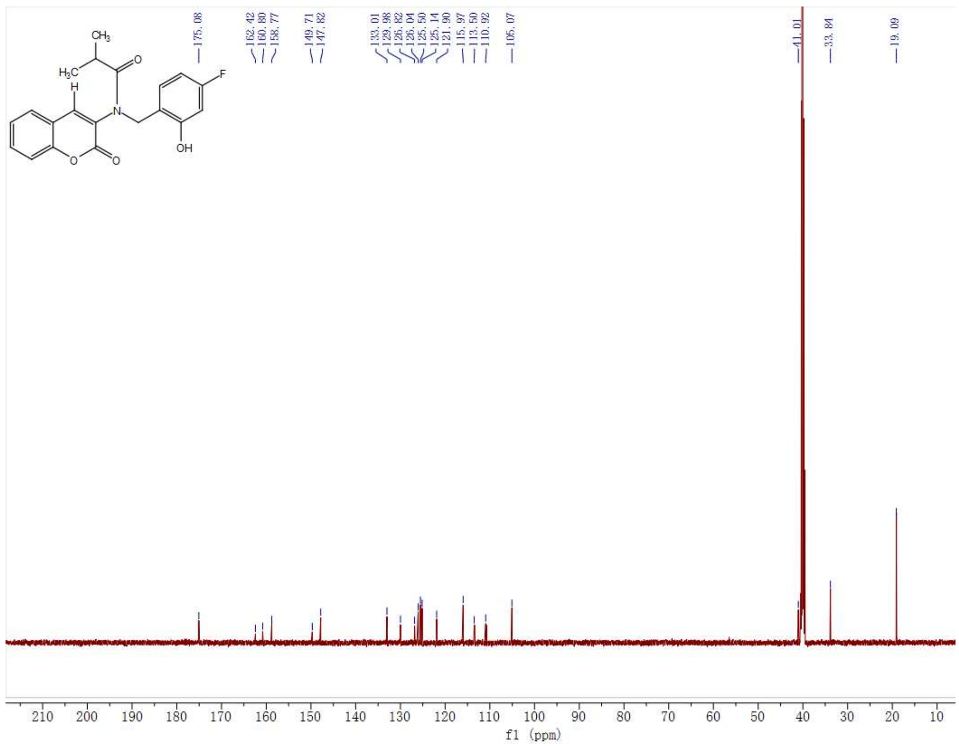

^{1}H NMR of compound 4b.

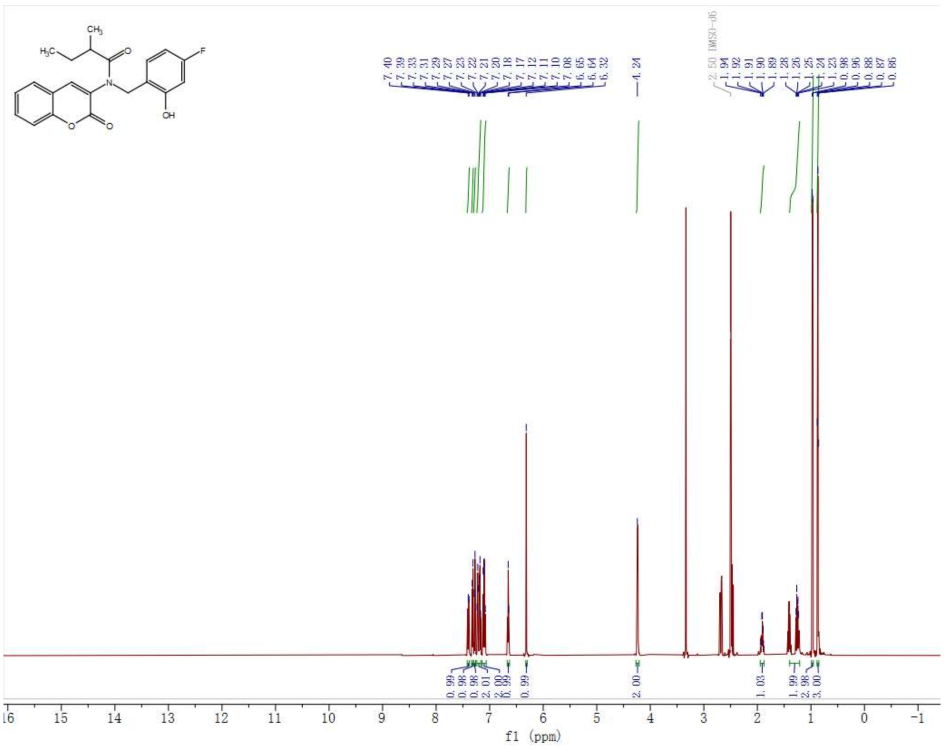

^{13}C NMR of compound 4b.

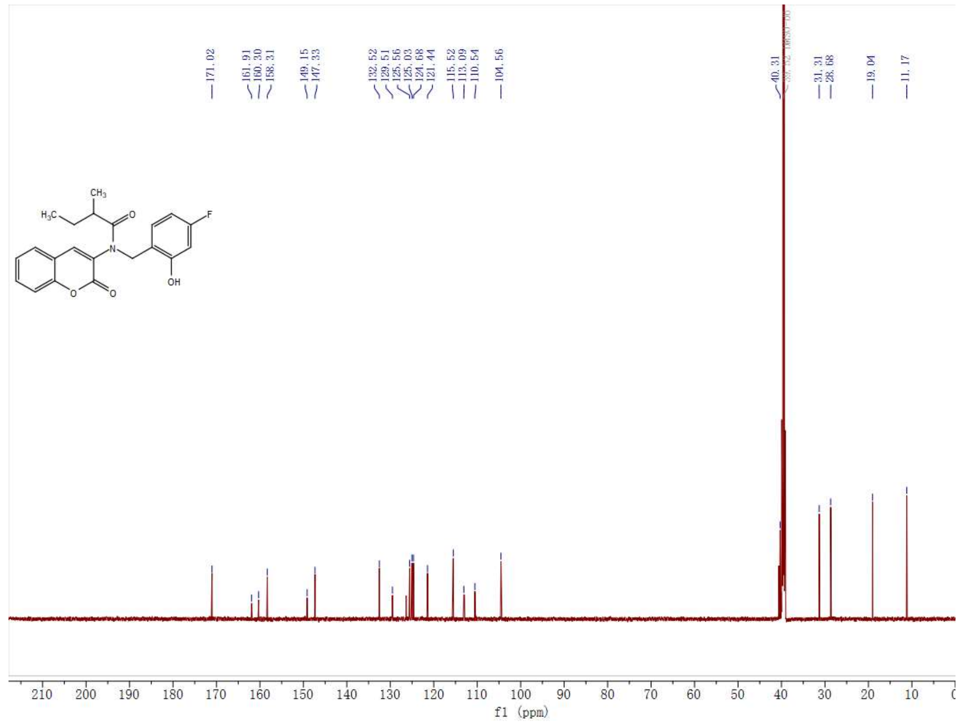

^{1}H NMR of compound 4c.

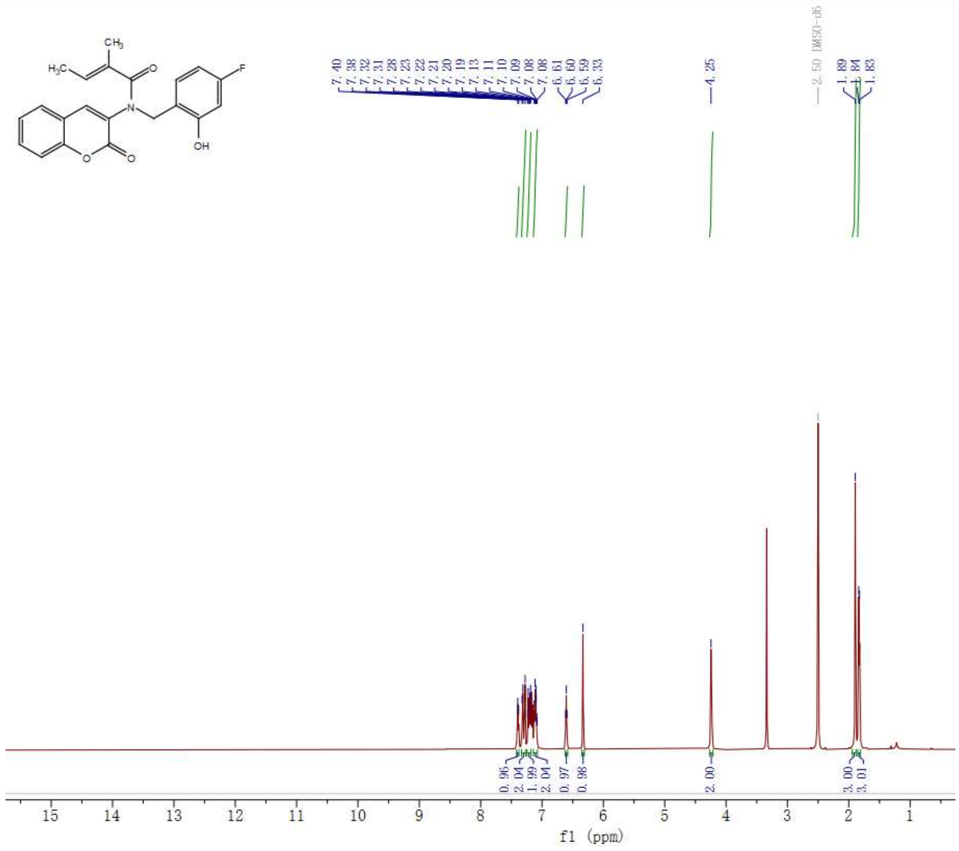

^{13}C NMR of compound 4c.

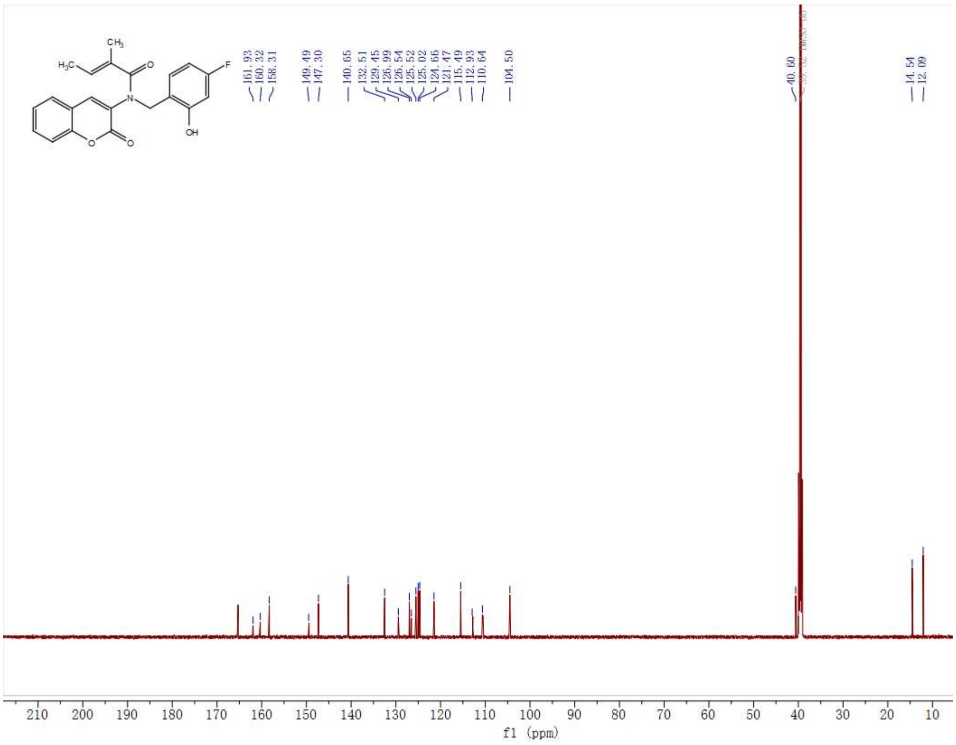

^{1}H NMR of compound 4d.

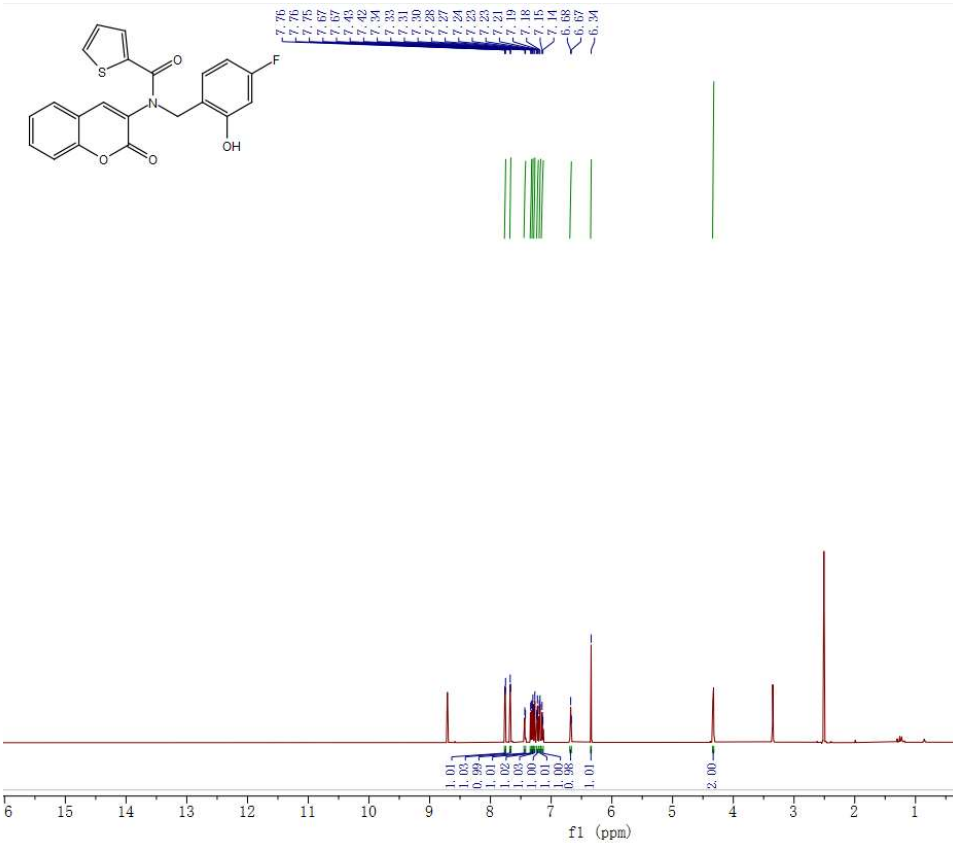

^{13}C NMR of compound 4d.

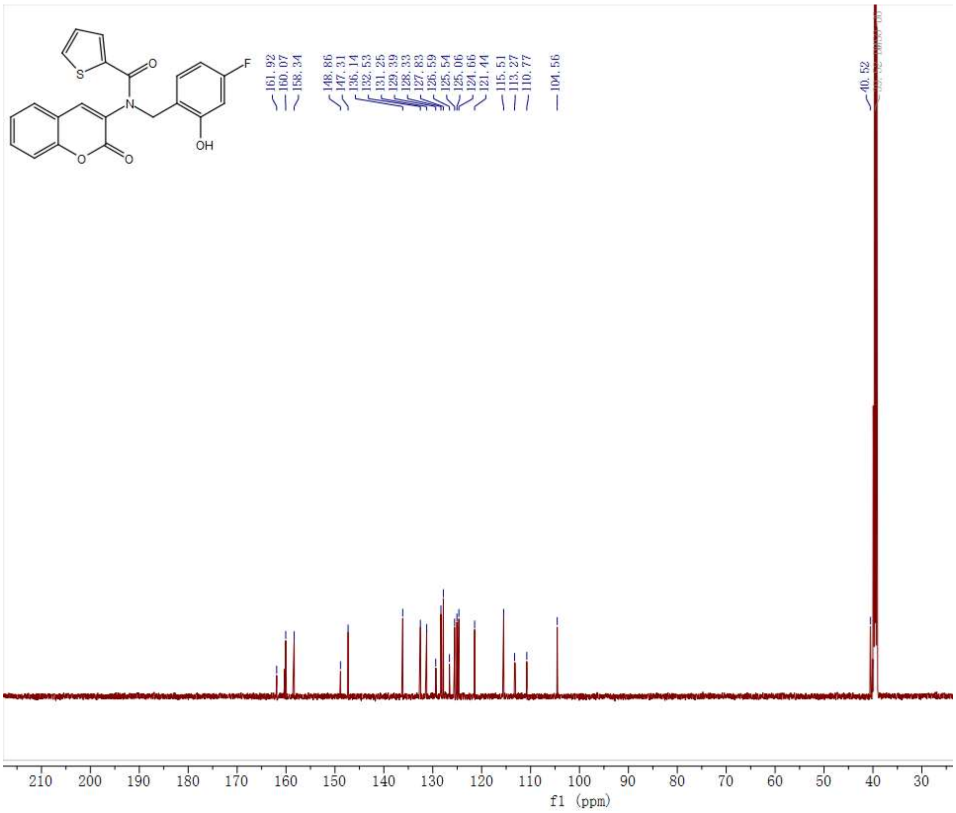

^{1}H NMR of compound 4e.

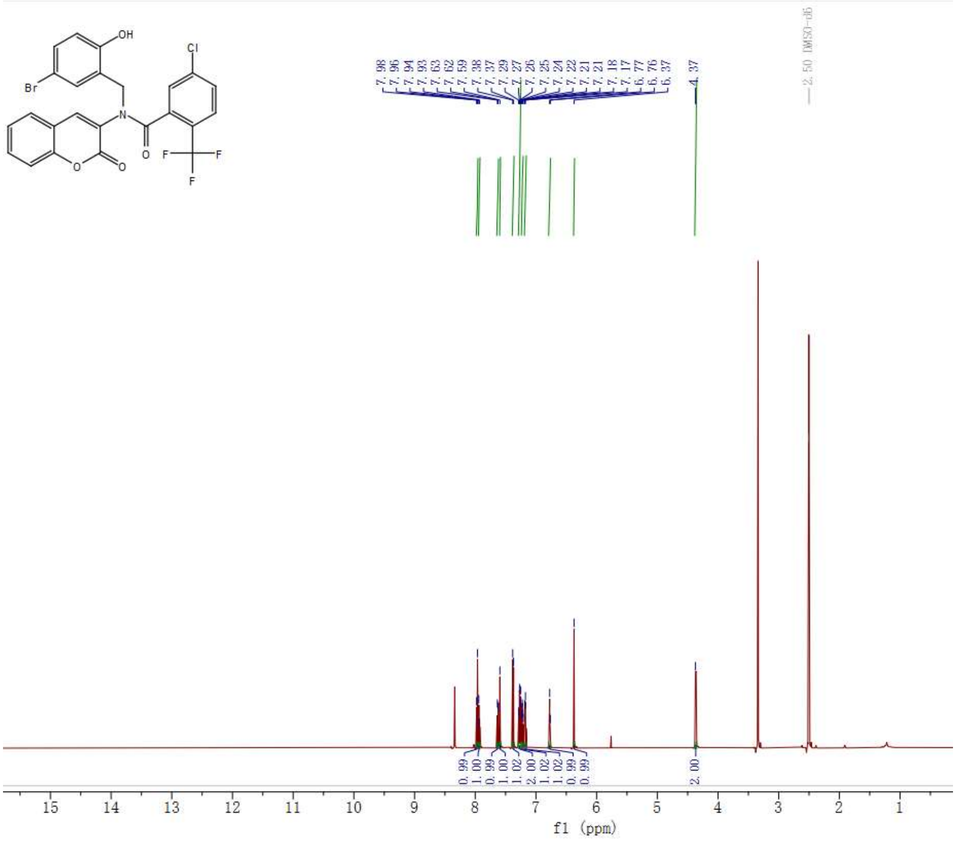

^{13}C NMR of compound 4e.

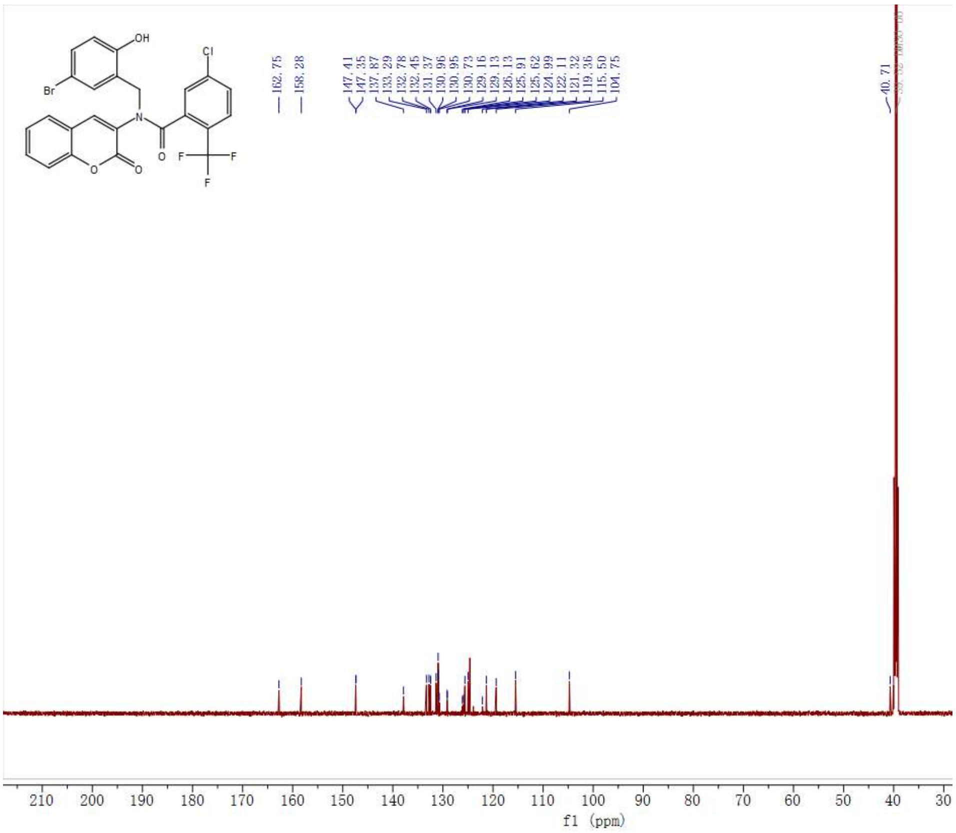

^{1}H NMR of compound 4f.

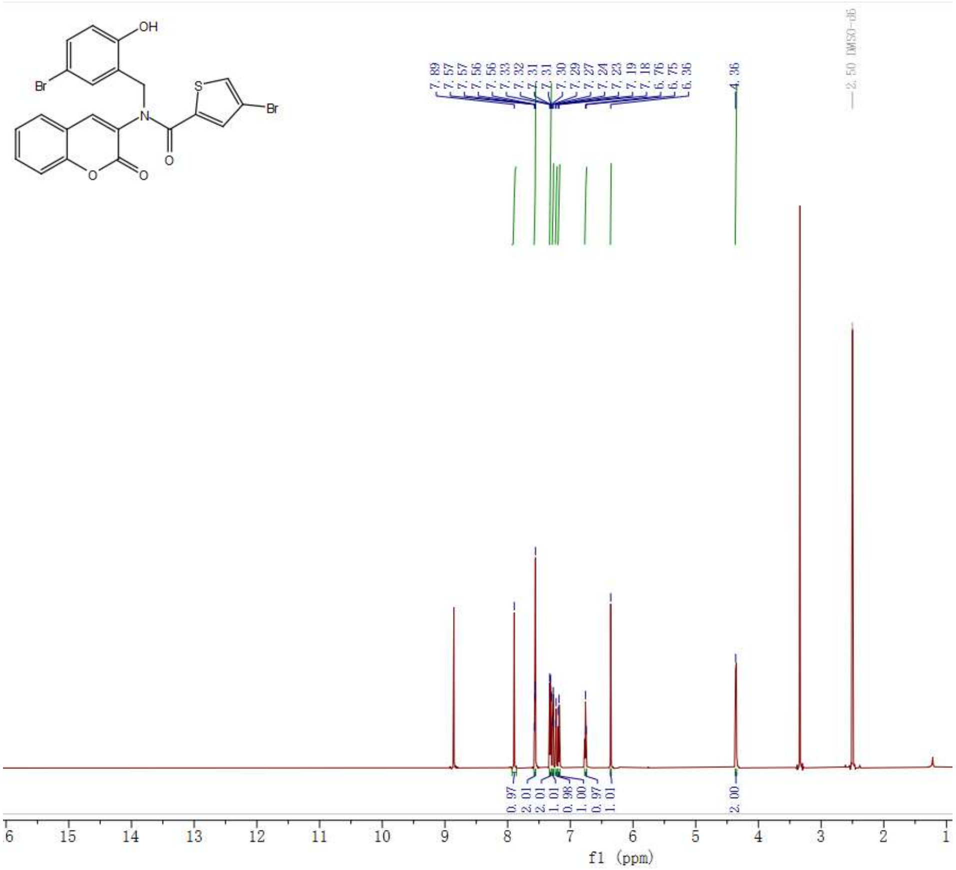

^{13}C NMR of compound 4f.

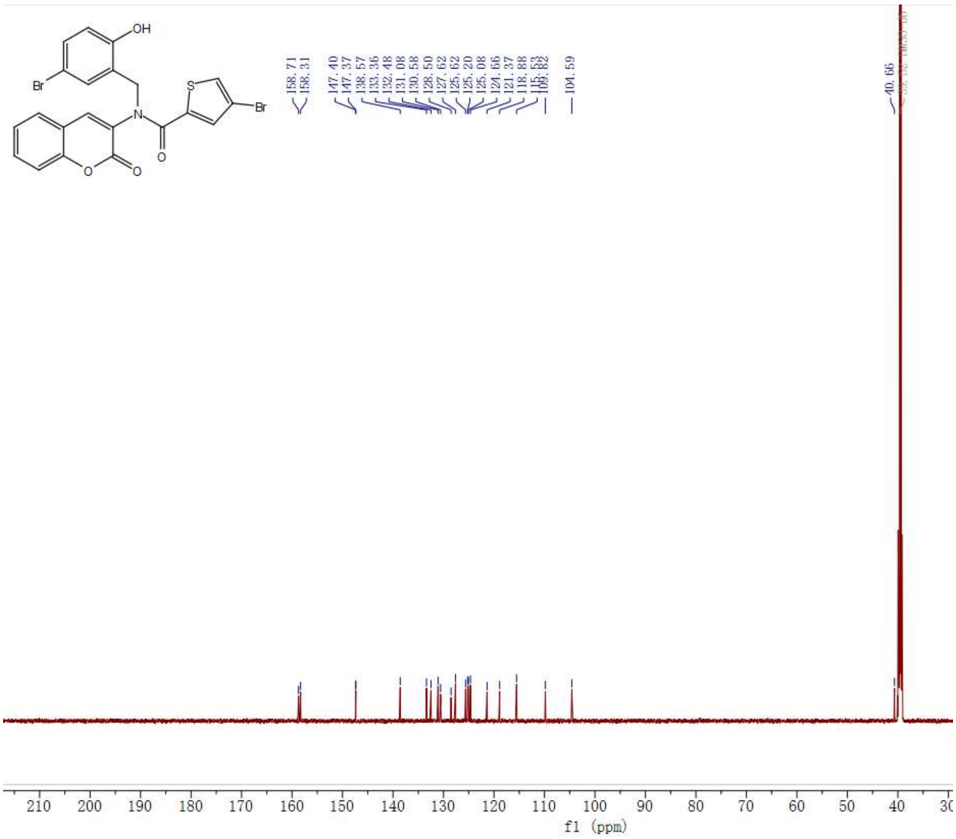

^{1}H NMR of compound 4g.

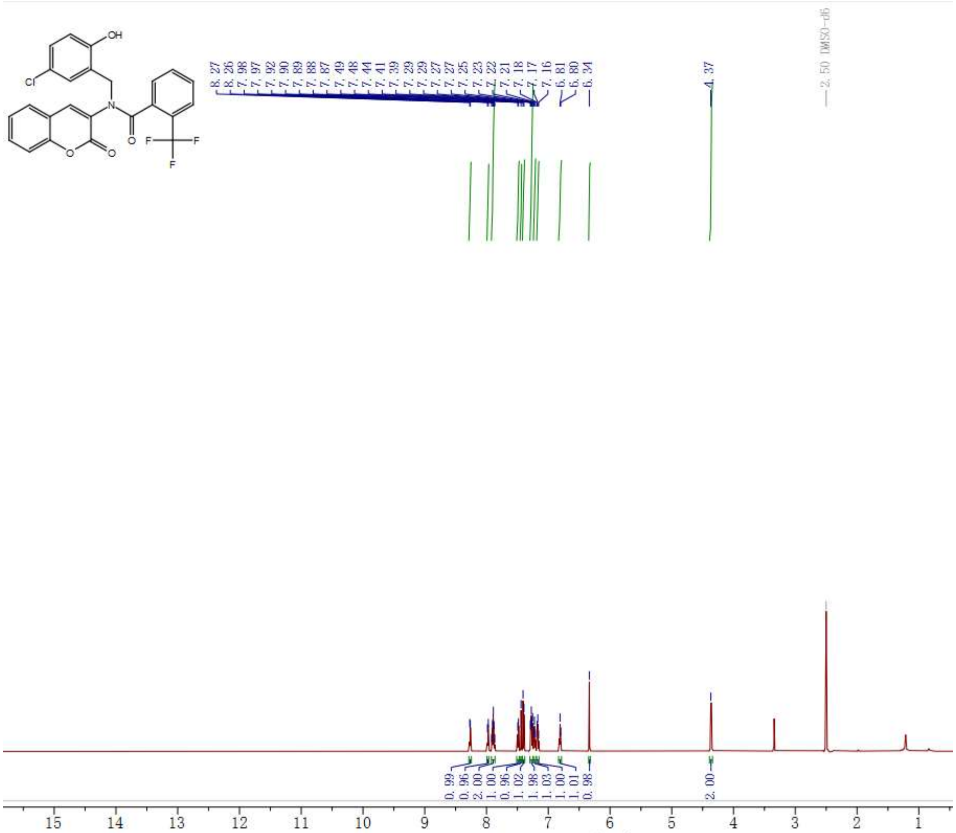

^{13}C NMR of compound 4g.

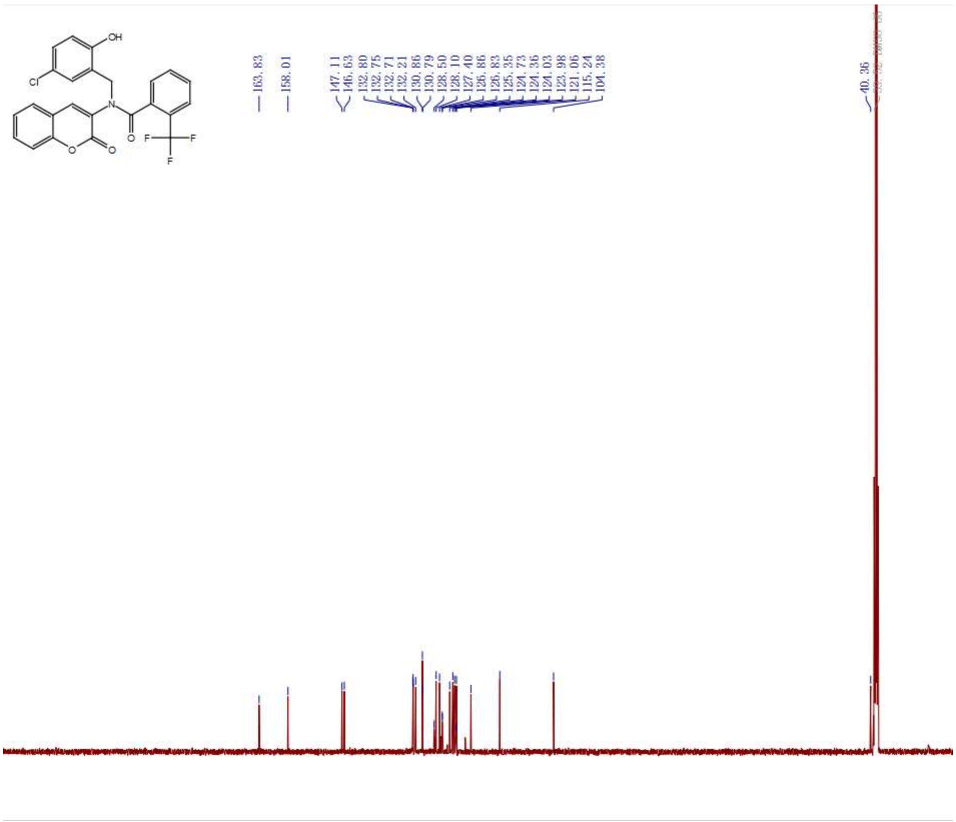

^{1}H NMR of compound 4h.

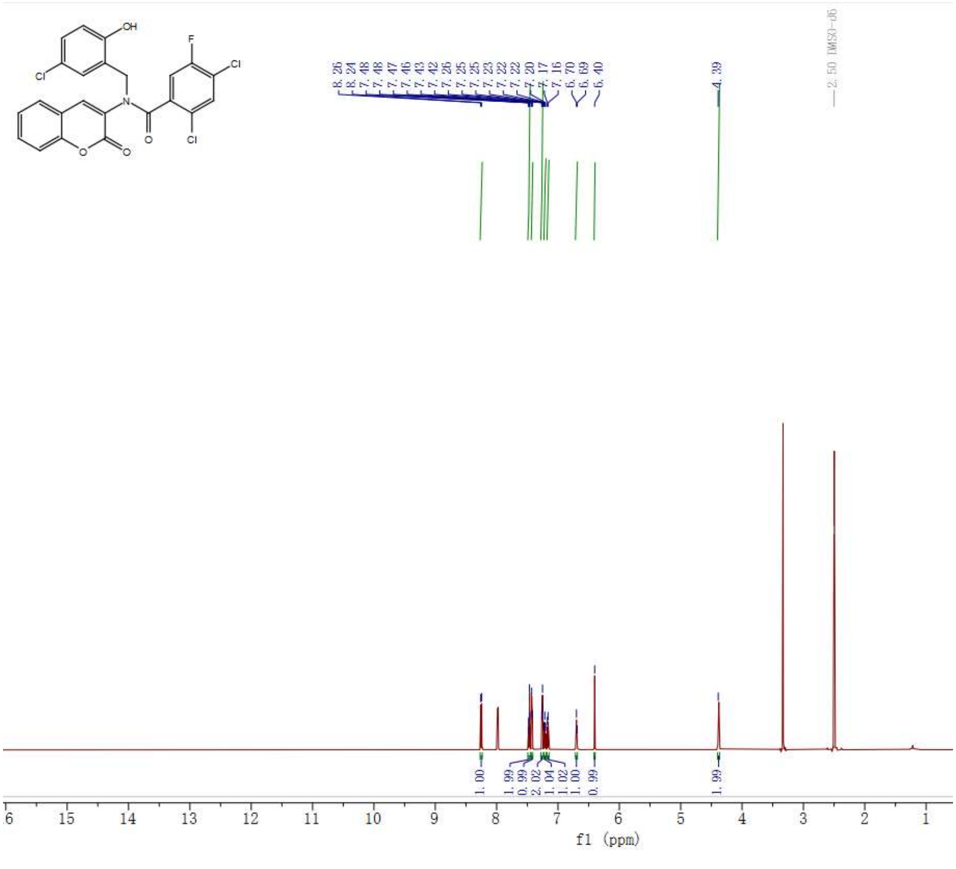

^{13}C NMR of compound 4h.

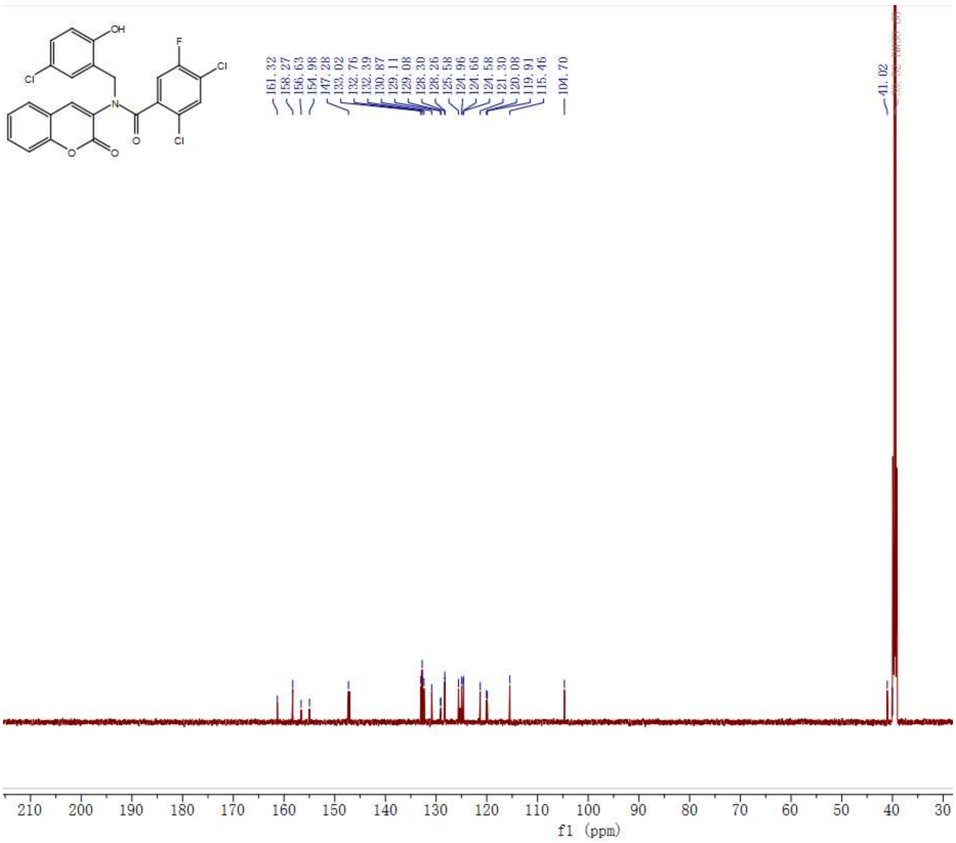

^{1}H **NMR of compound 4i.**

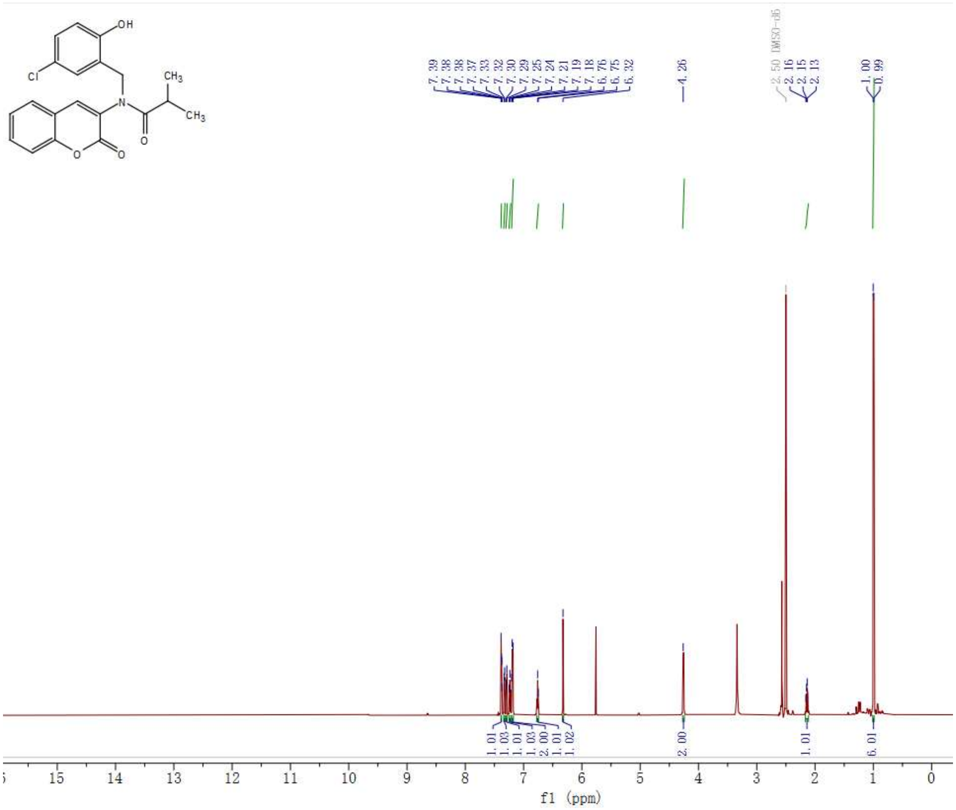

^{13}C **NMR of compound 4i.**

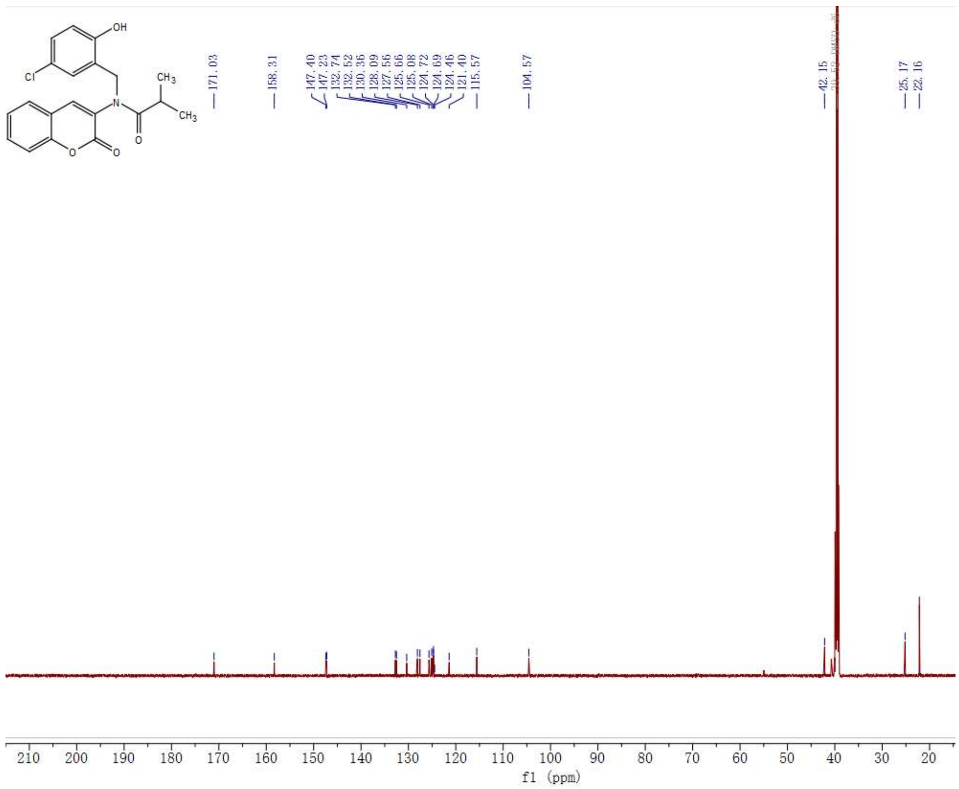

^{1}H NMR of compound 4j.

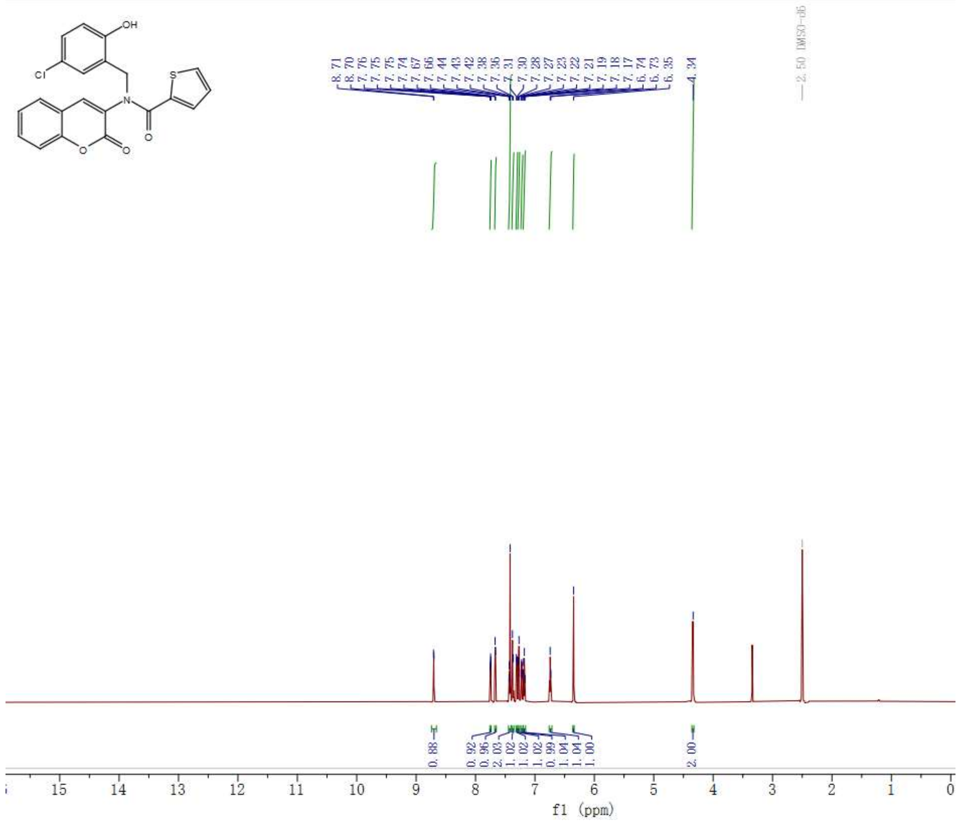

^{13}C NMR of compound 4j.

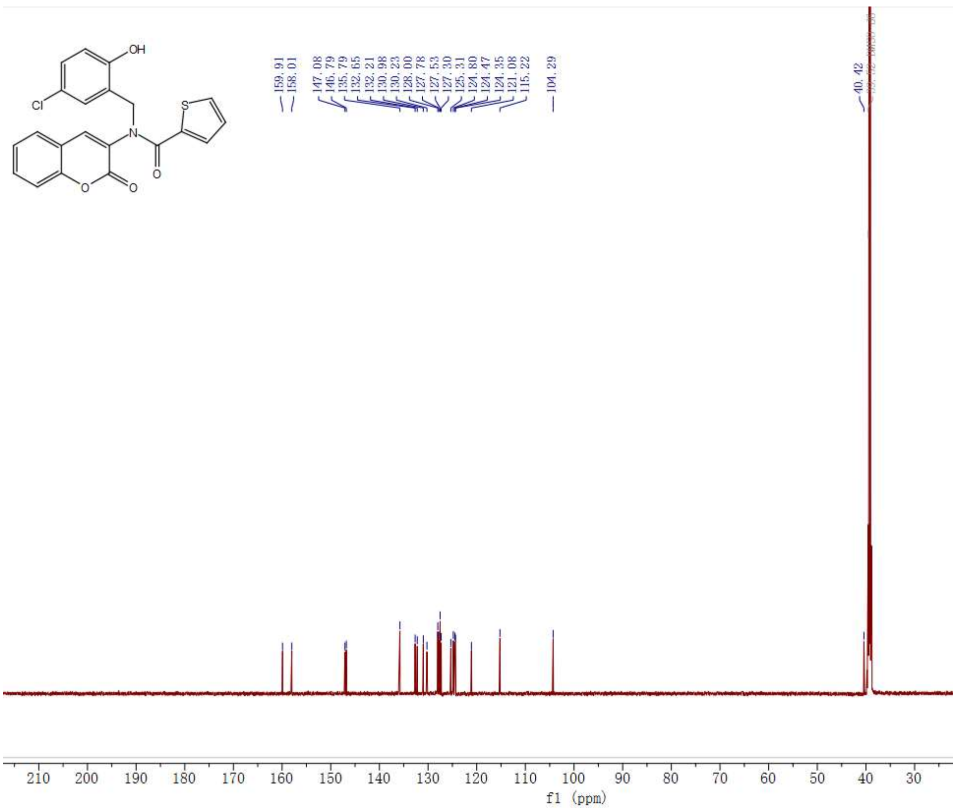

^{1}H NMR of compound 4k.

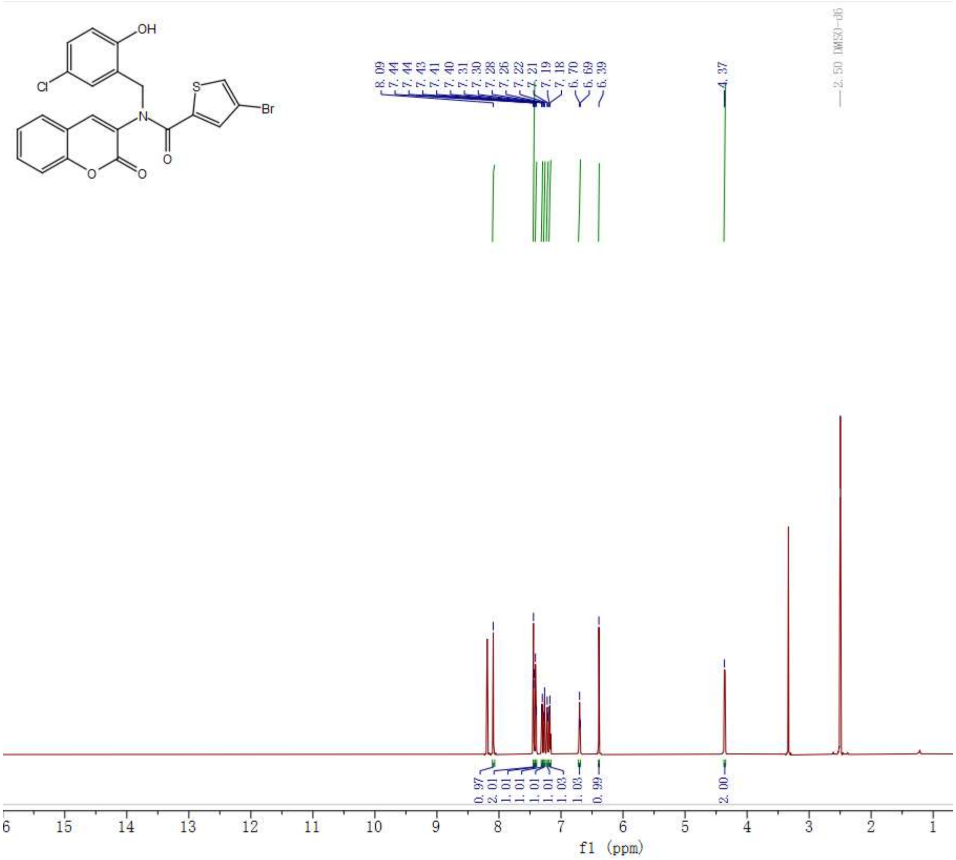

^{13}C NMR of compound 4k.

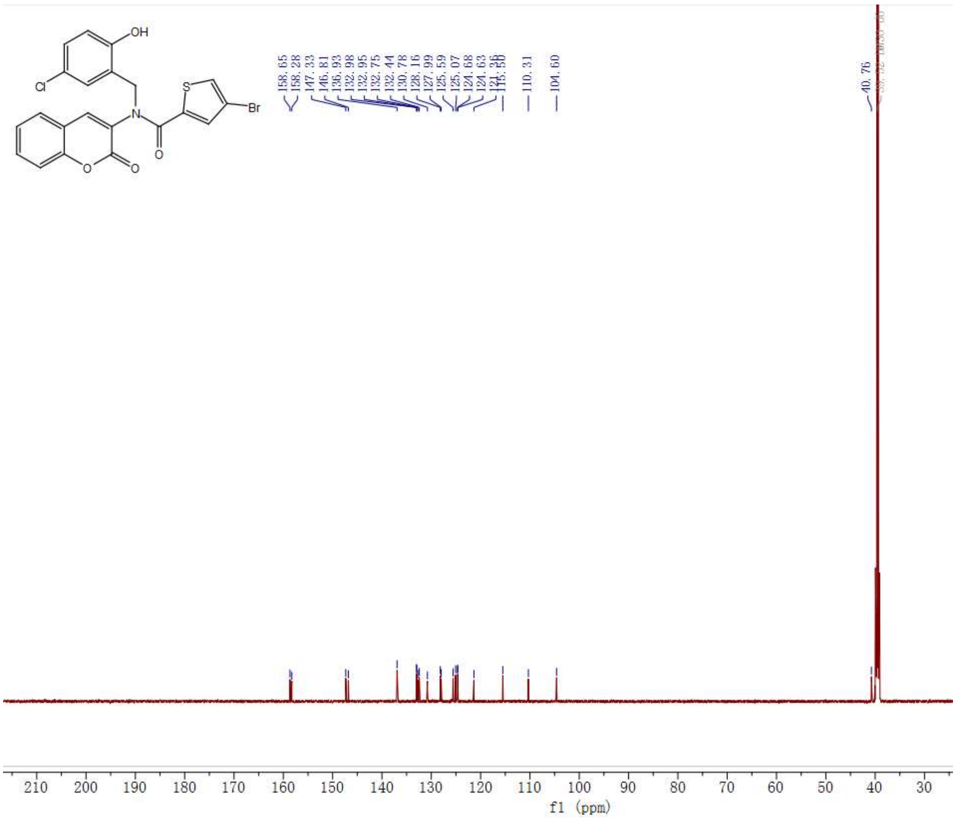

^{1}H NMR of compound 4l.

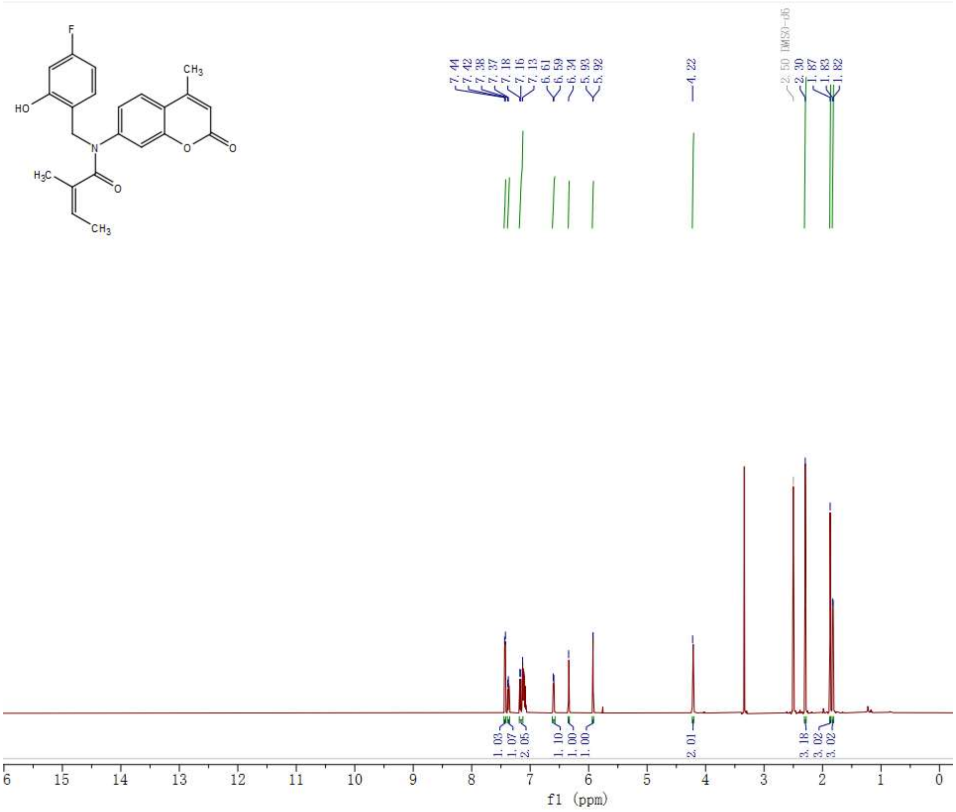

^{13}C NMR of compound 4l.

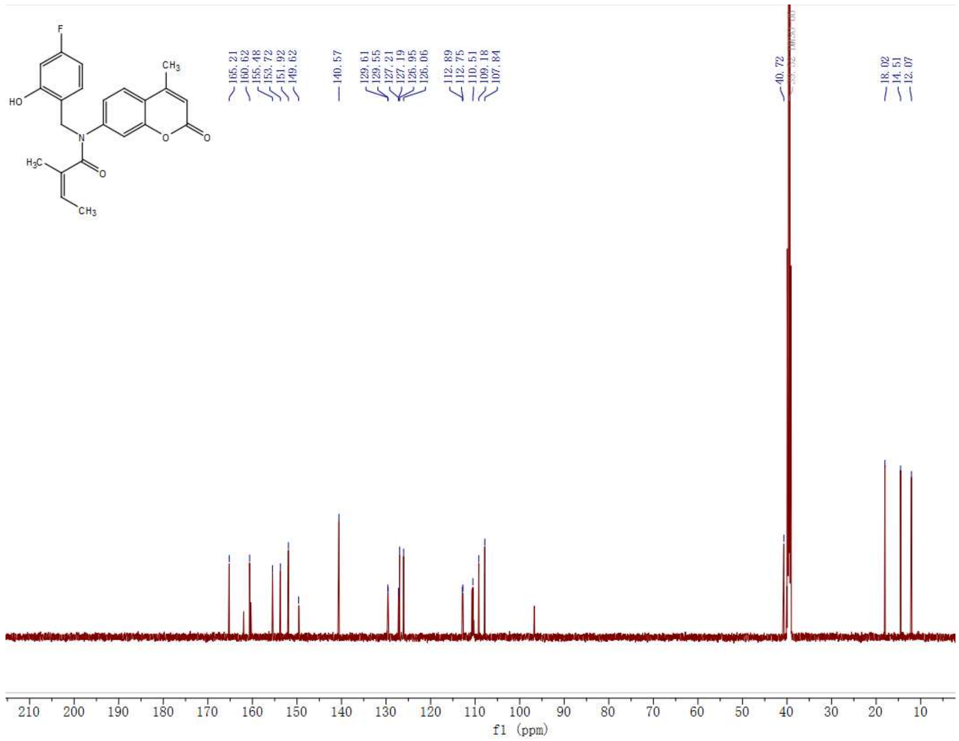

^{1}H NMR of compound 4m.

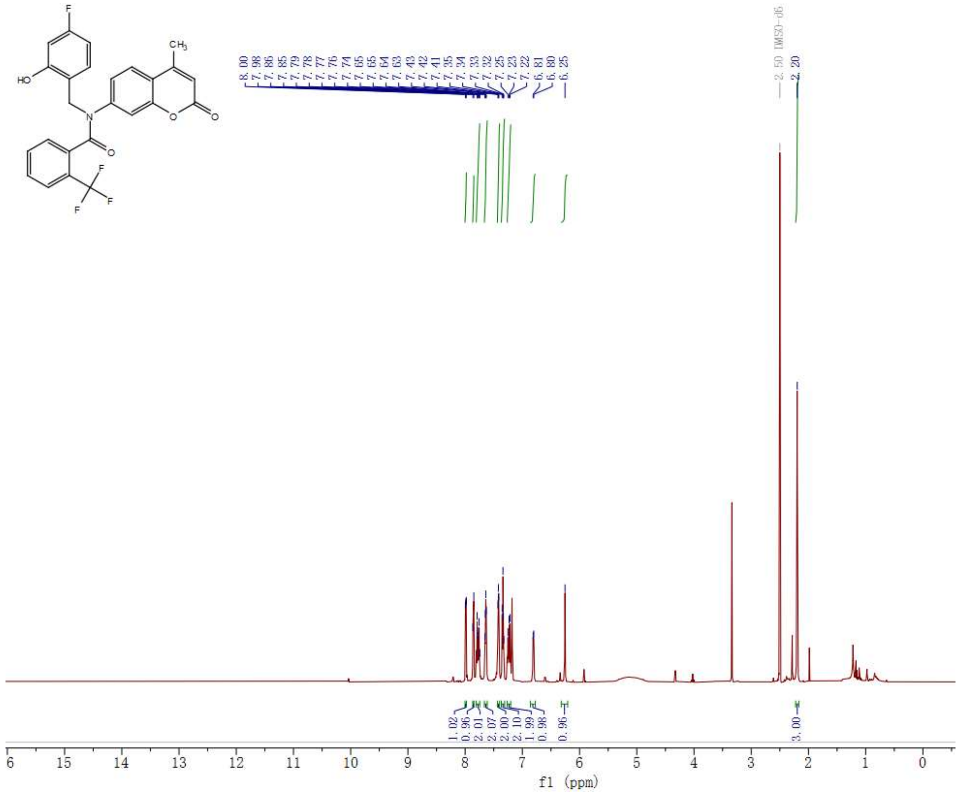

^{13}C NMR of compound 4m.

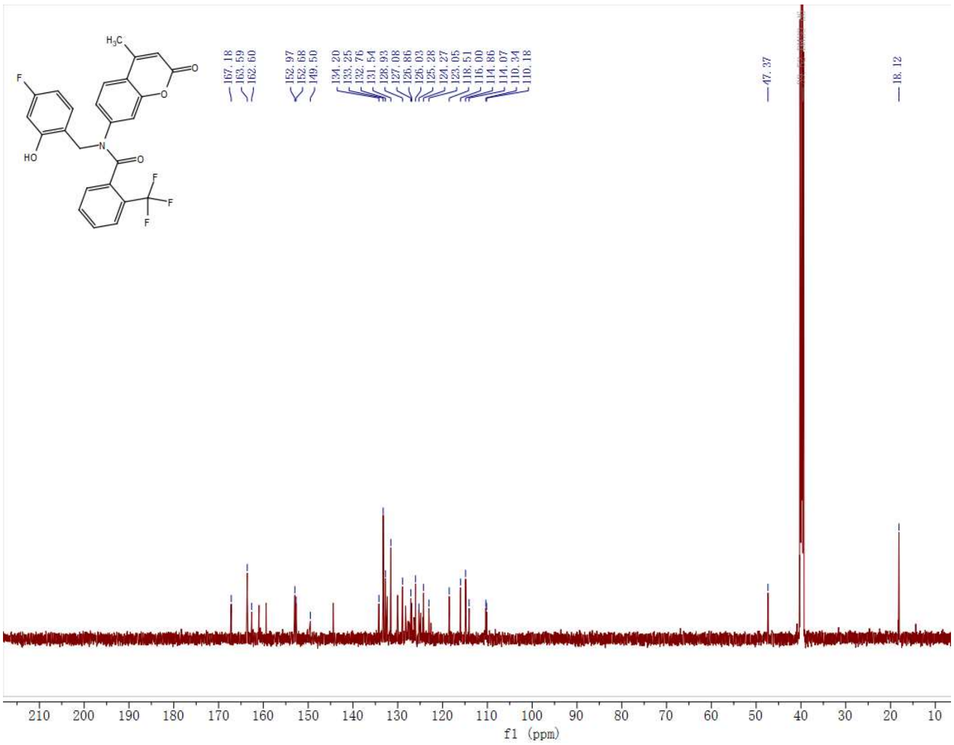

^{1}H NMR of compound 4n.

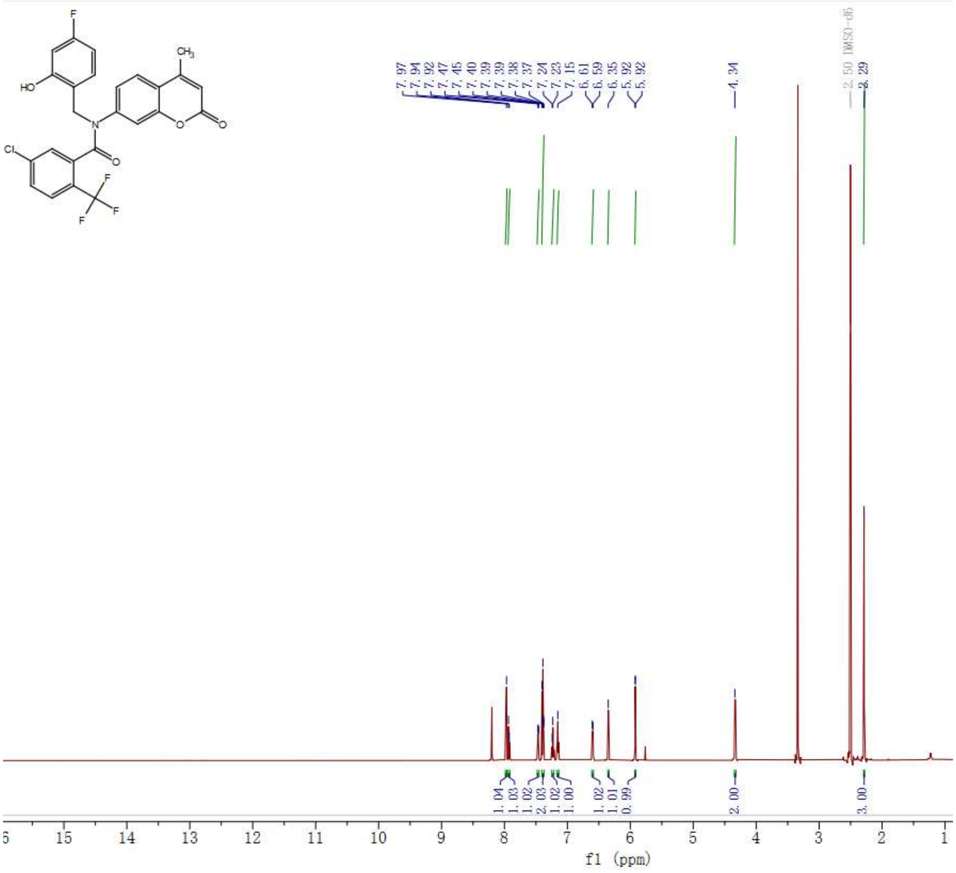

^{13}C NMR of compound 4n.

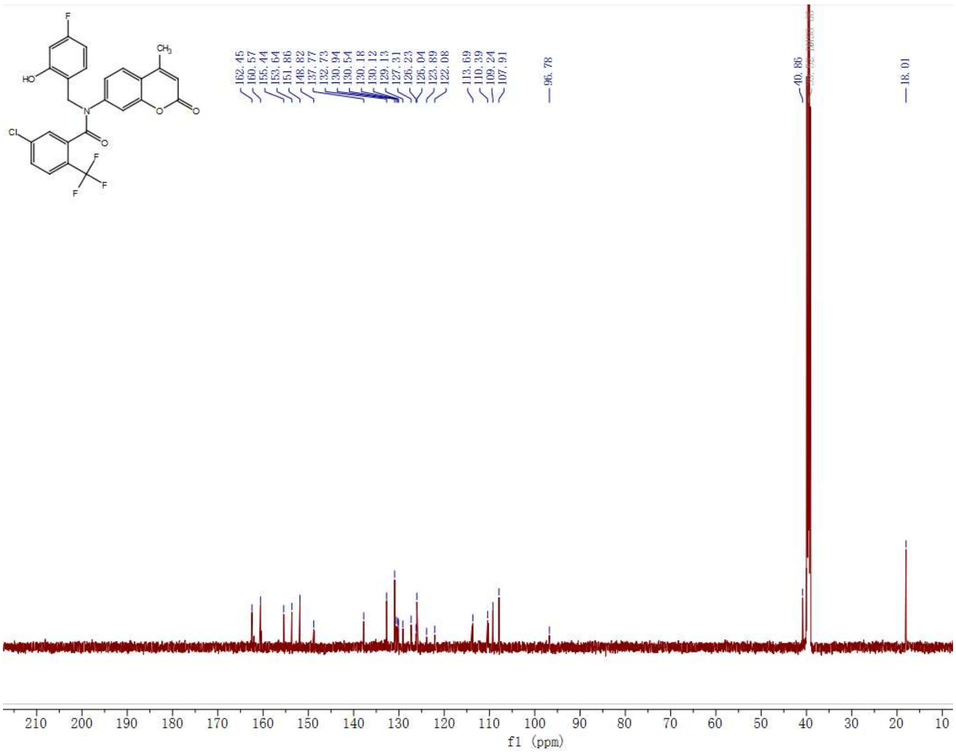

^{1}H NMR of compound 4o.

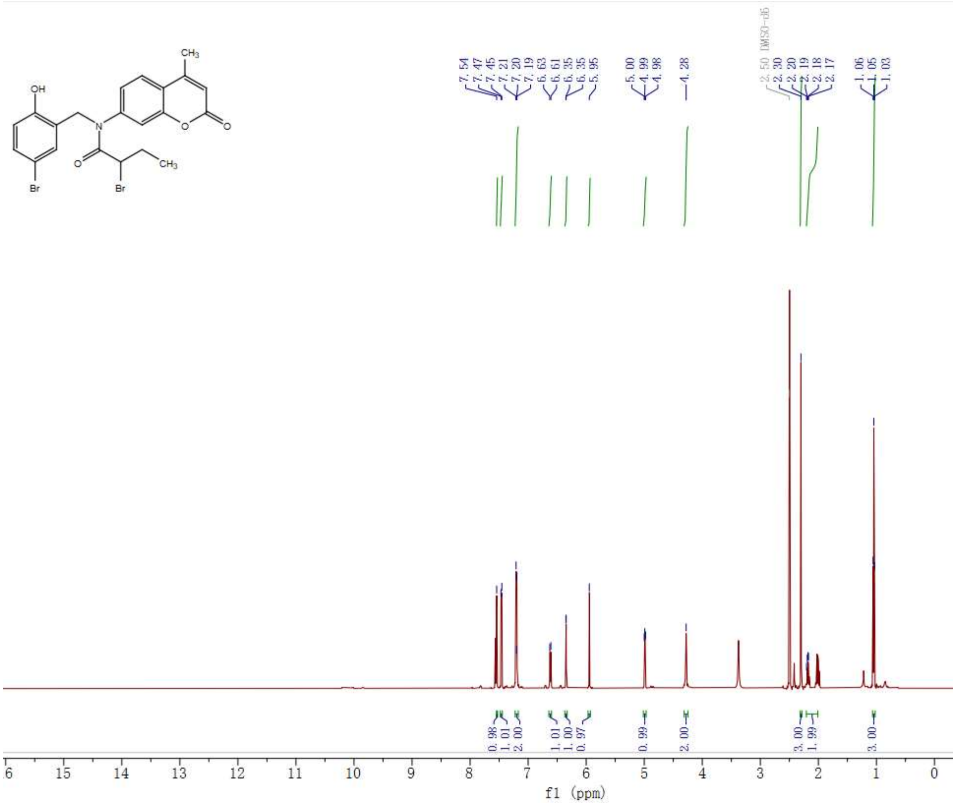

^{13}C NMR of compound 4o.

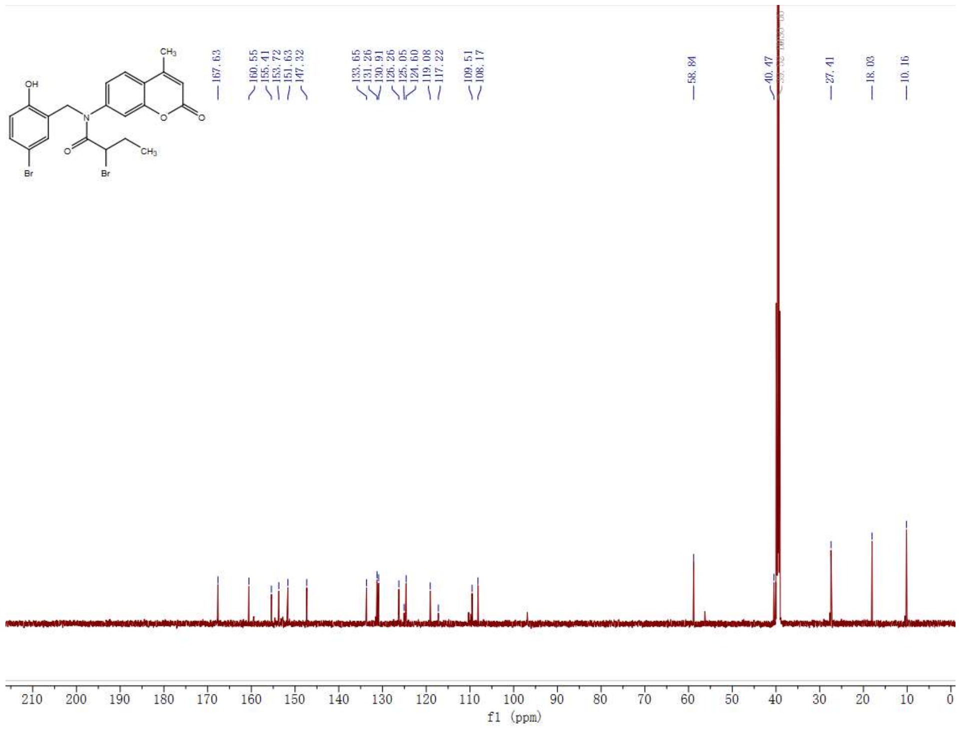

HPLC of compound 4o

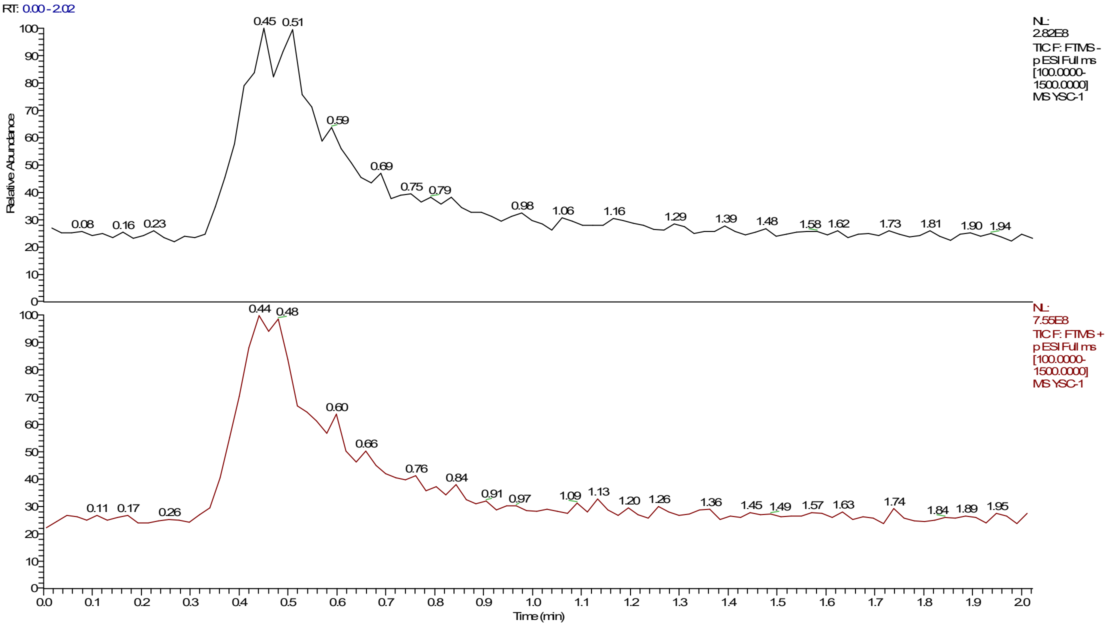

HRMS of compound 4o

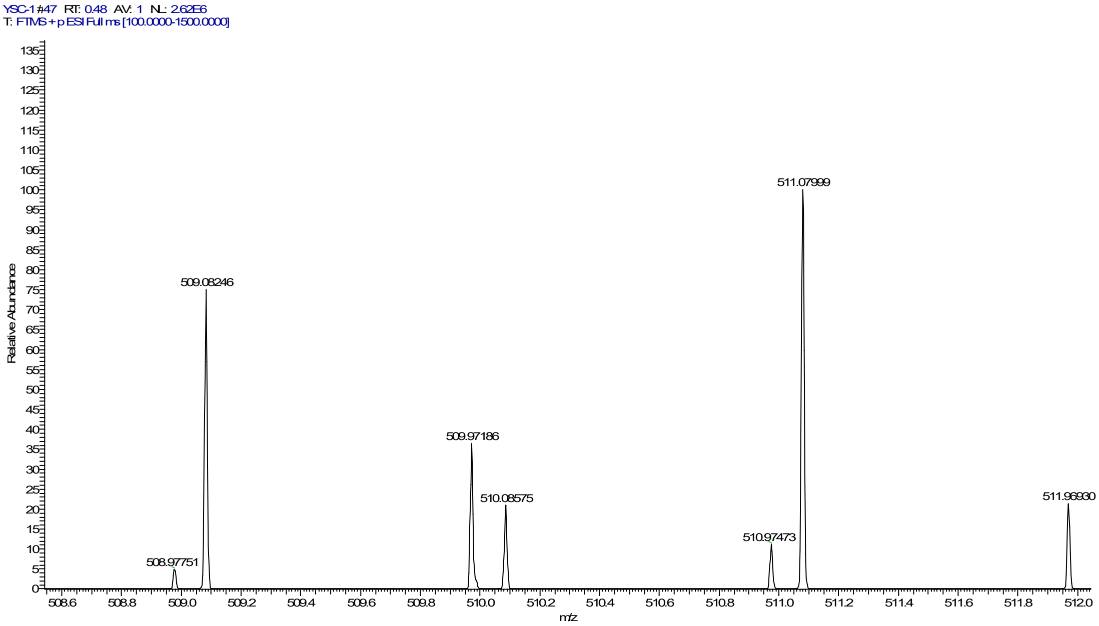

^{1}H NMR of compound 4p.

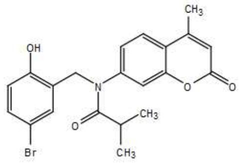

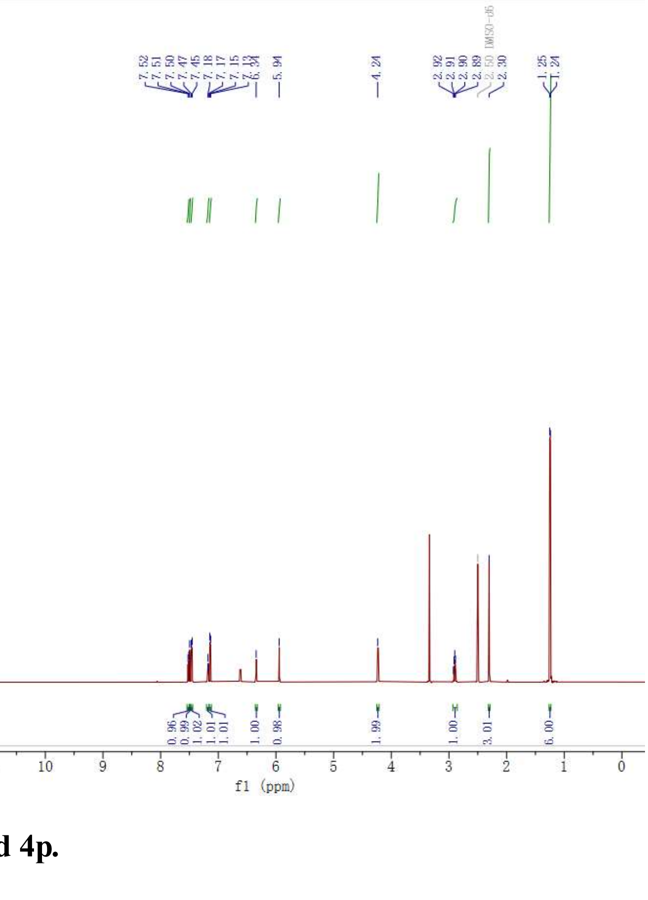

^{13}C NMR of compound 4p.

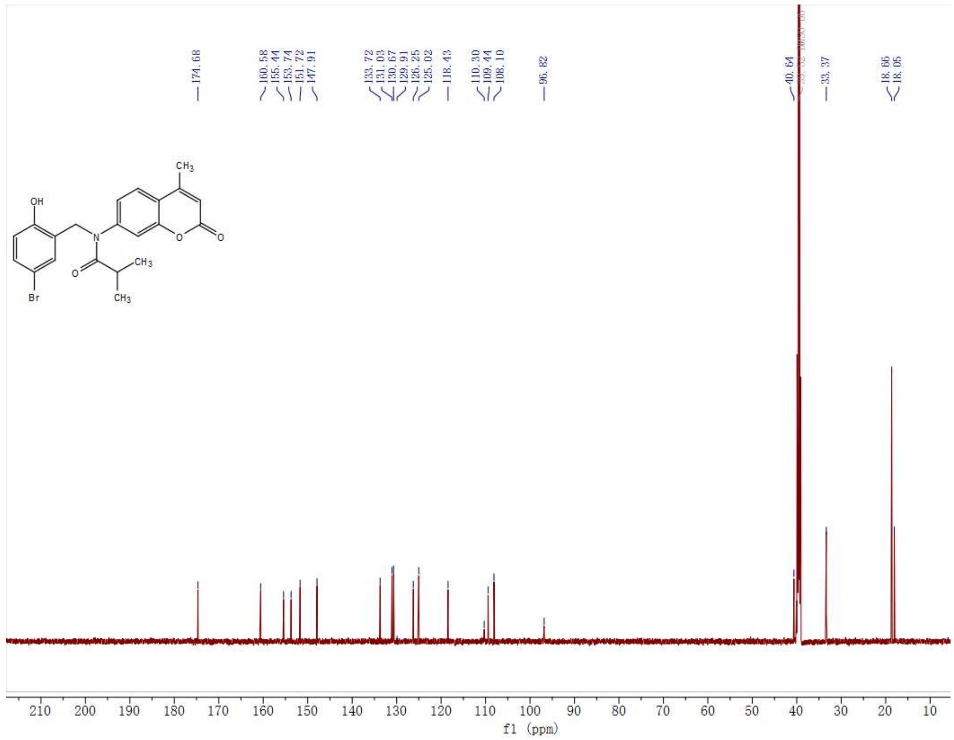

HPLC of compound 4o

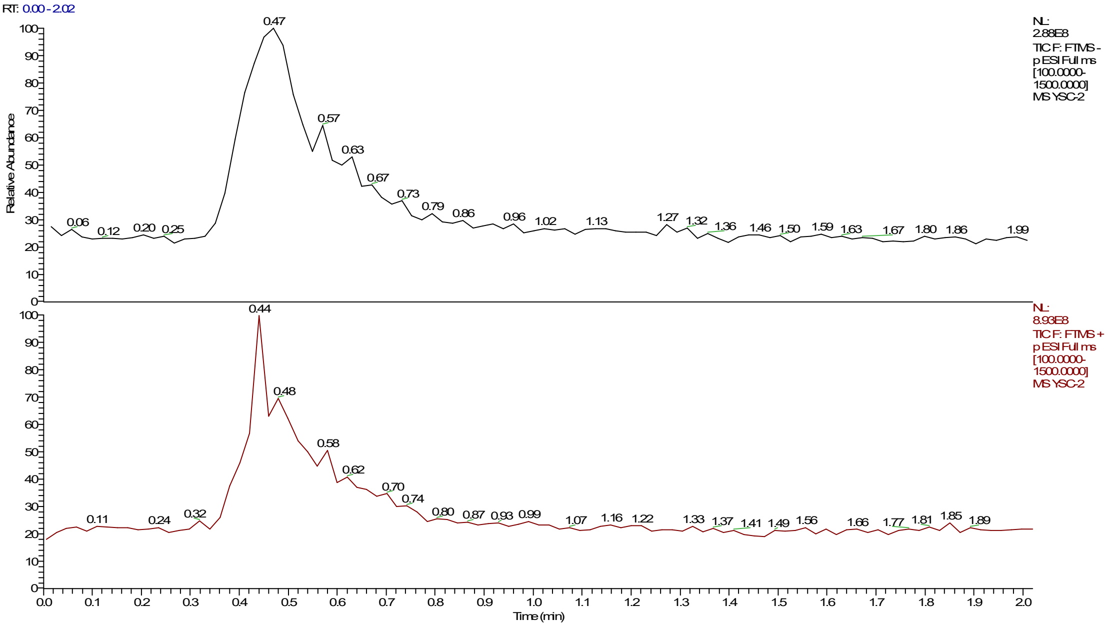

HRMS of compound 4p

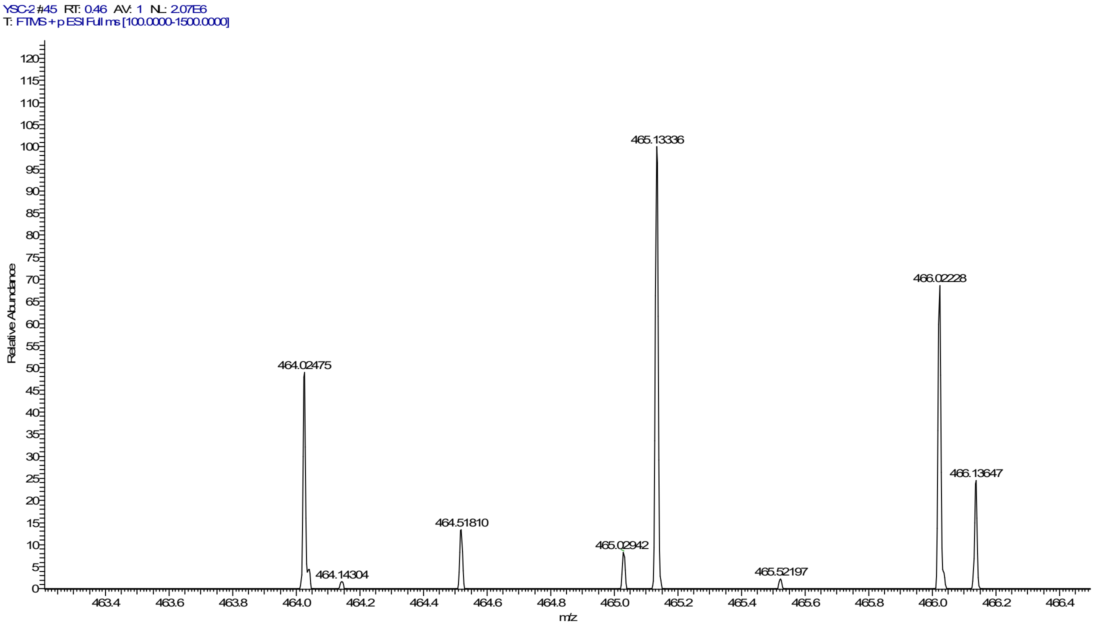

^{1}H NMR of compound 4q.

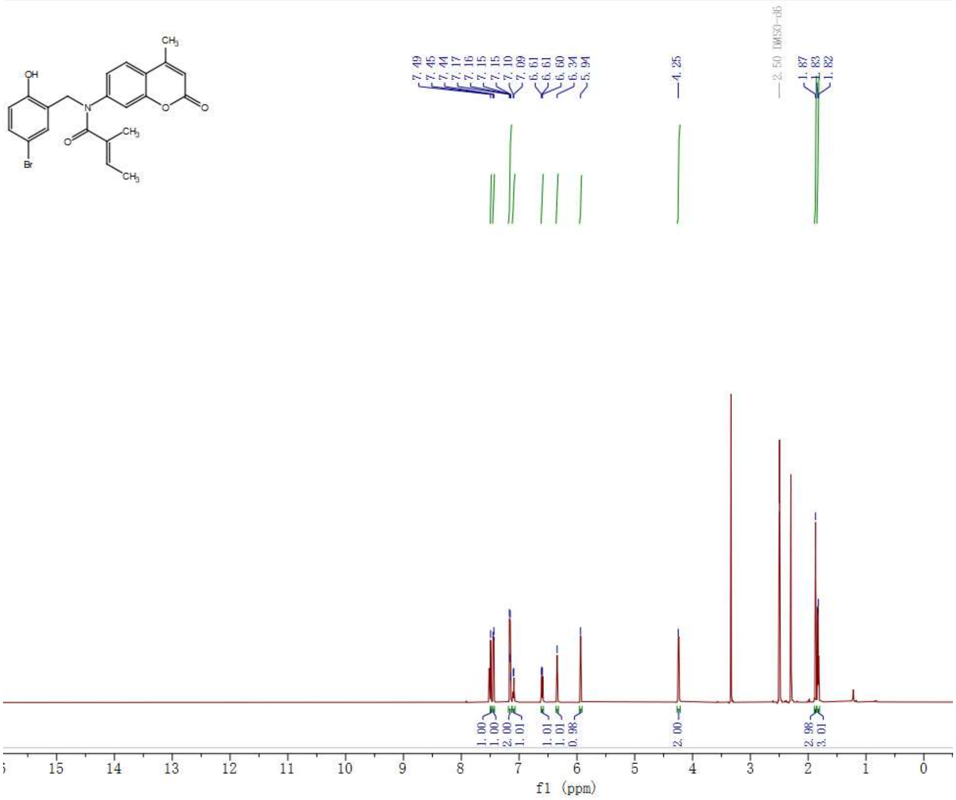

^{13}C NMR of compound 4q.

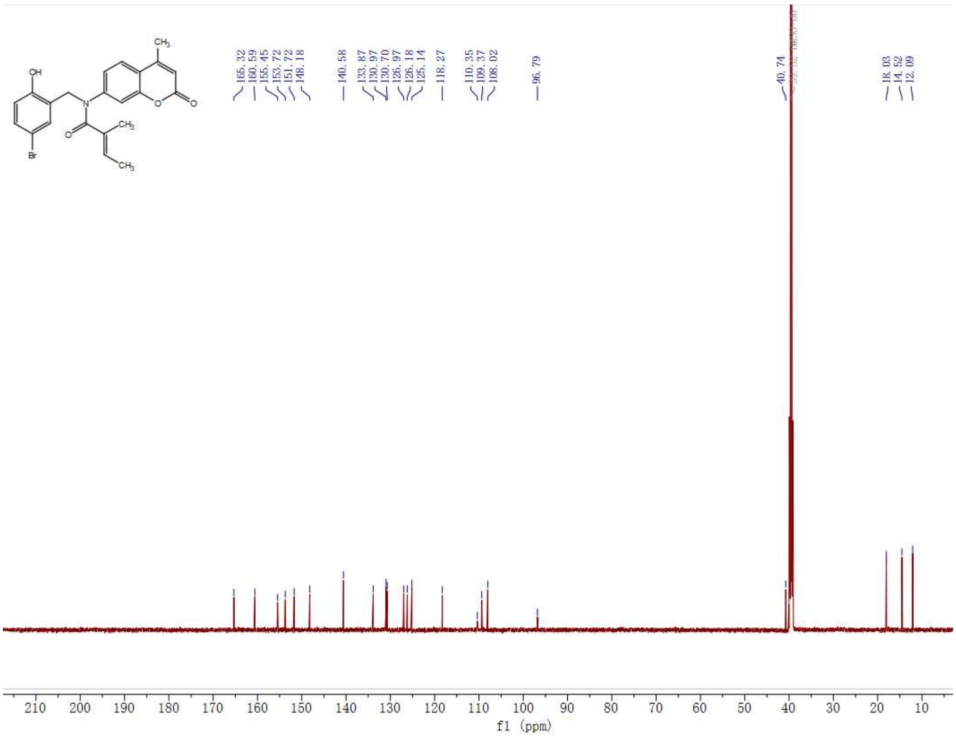

¹H NMR of compound 4r.

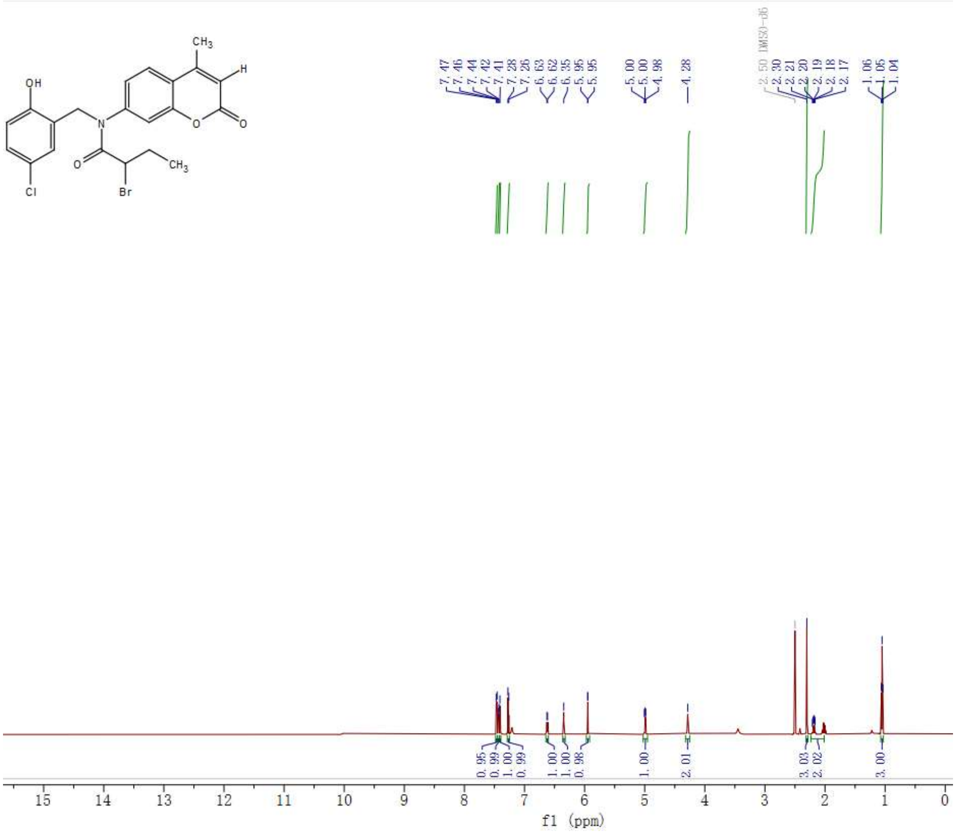

¹³C NMR of compound 4r.

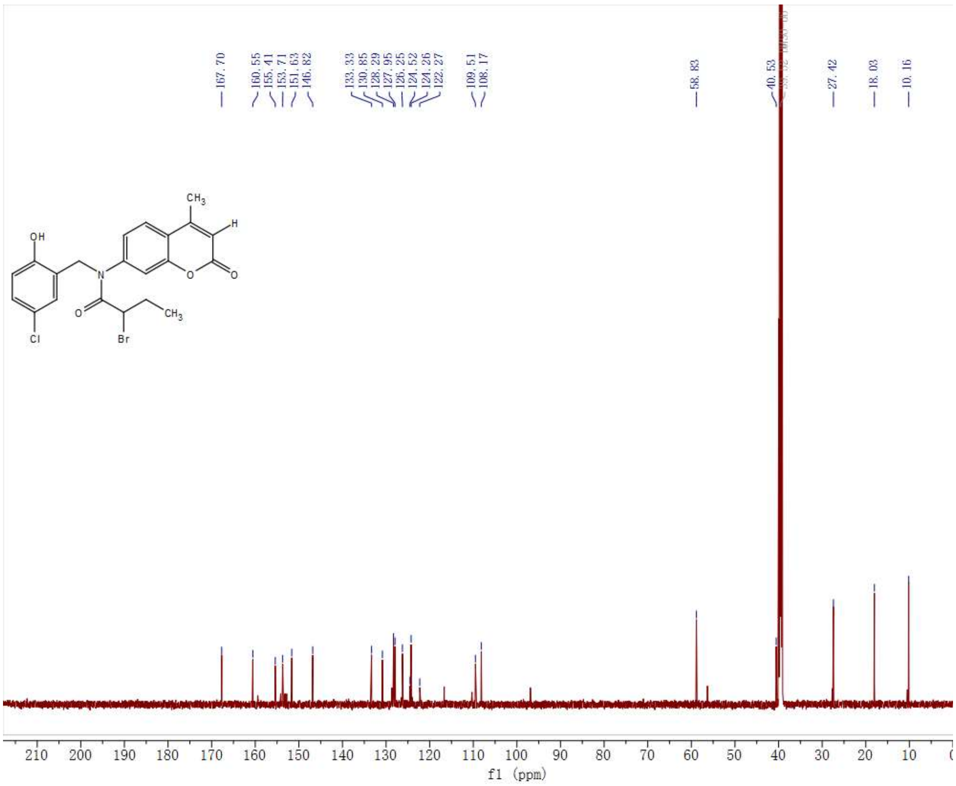

HPLC of compound 4r

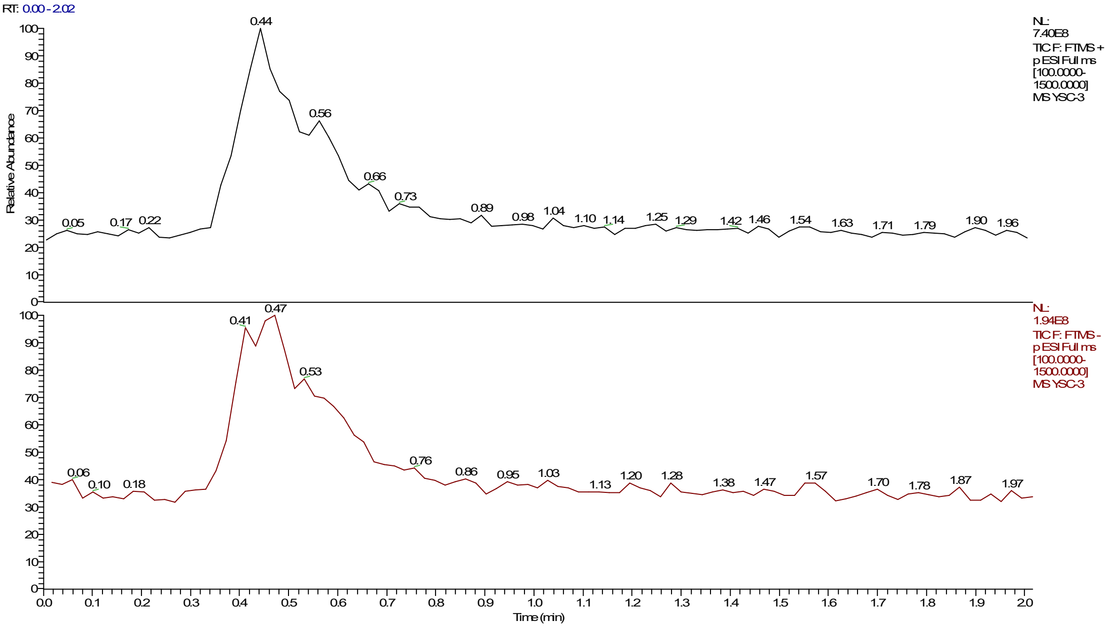

HRMS of compound 4r

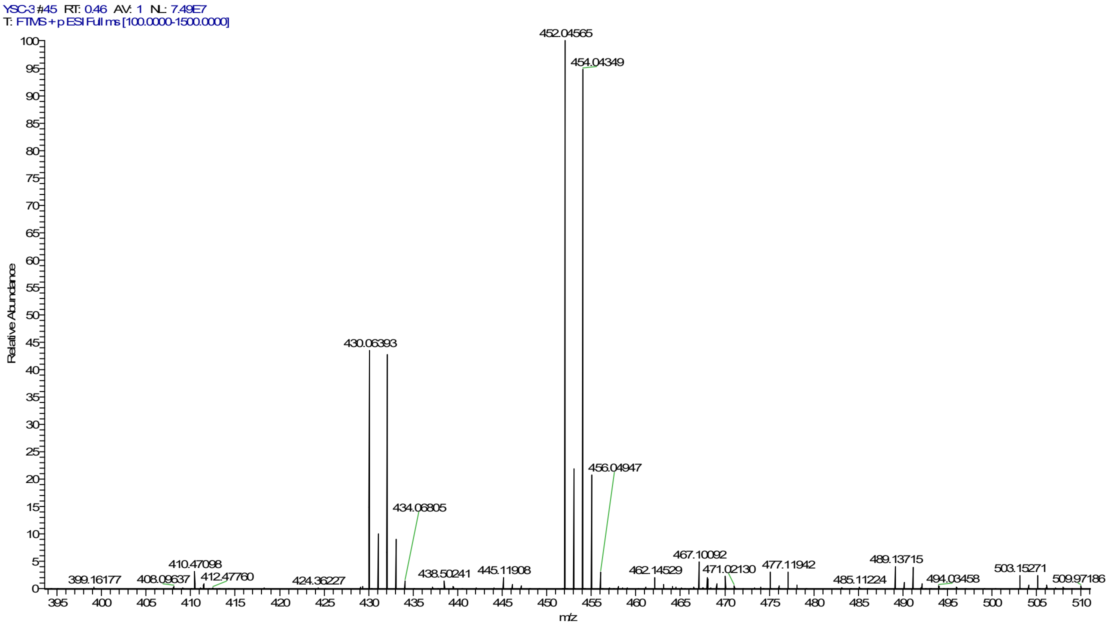

^{1}H NMR of compound 4s.

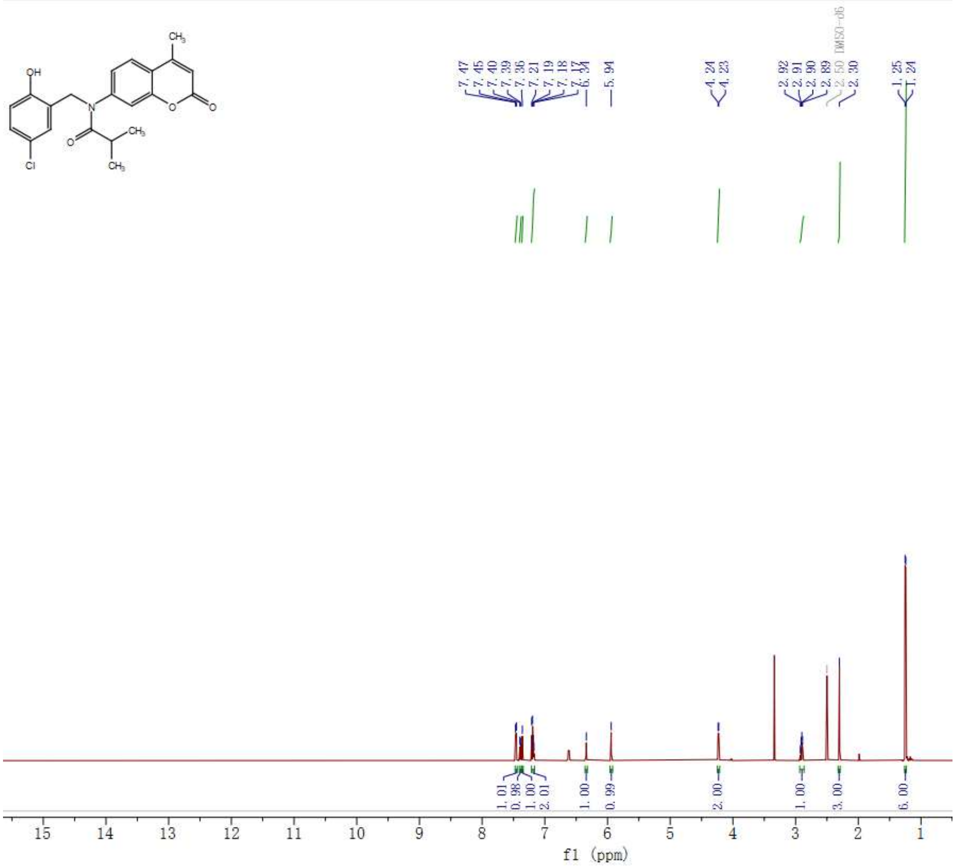

^{13}C NMR of compound 4s.

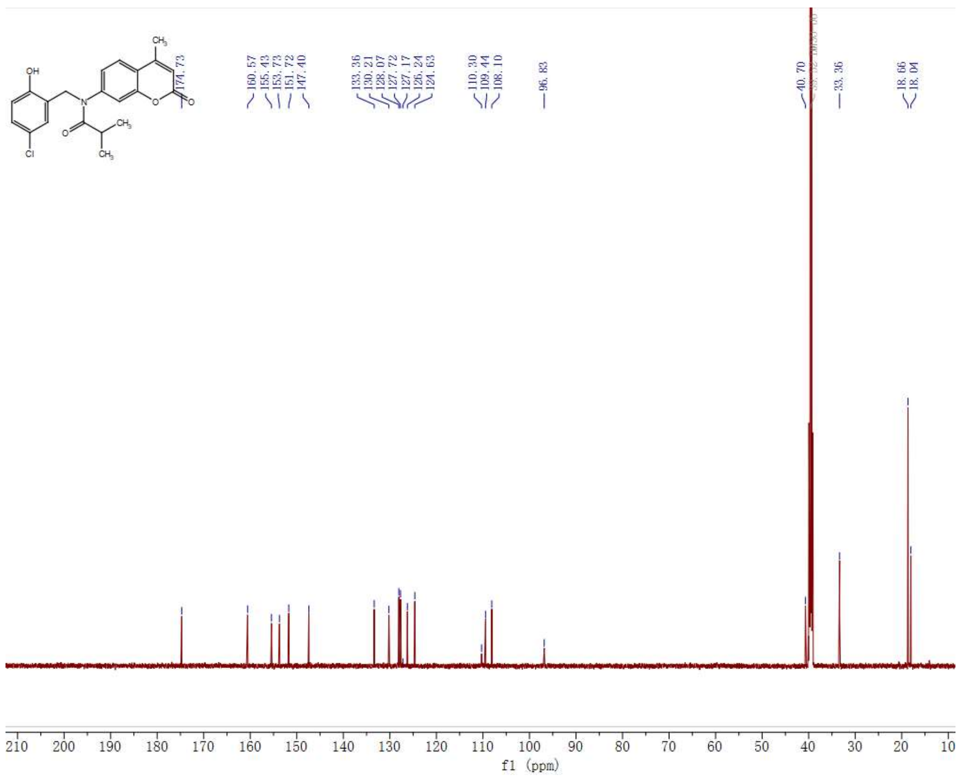

HPLC of compound 4s

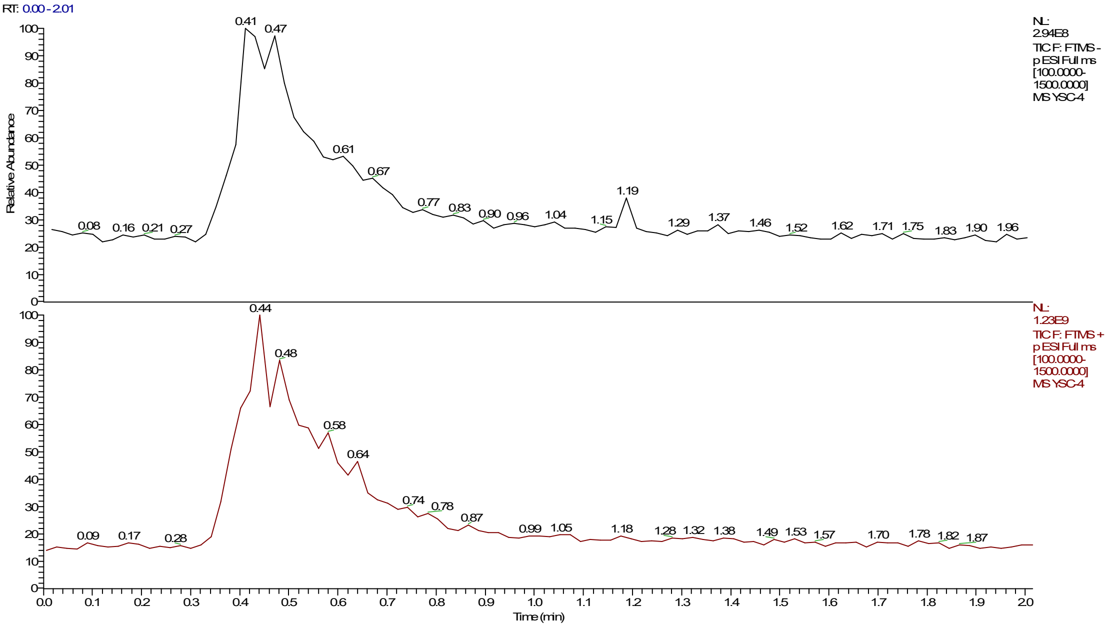

HRMS of compound 4s

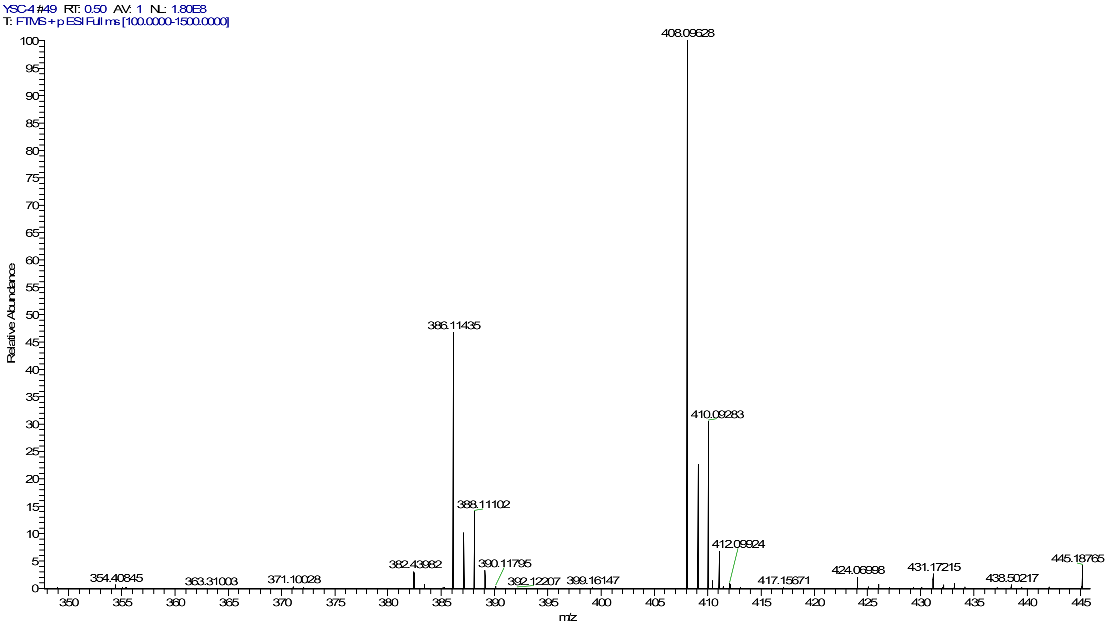

^{1}H NMR of compound 4t.

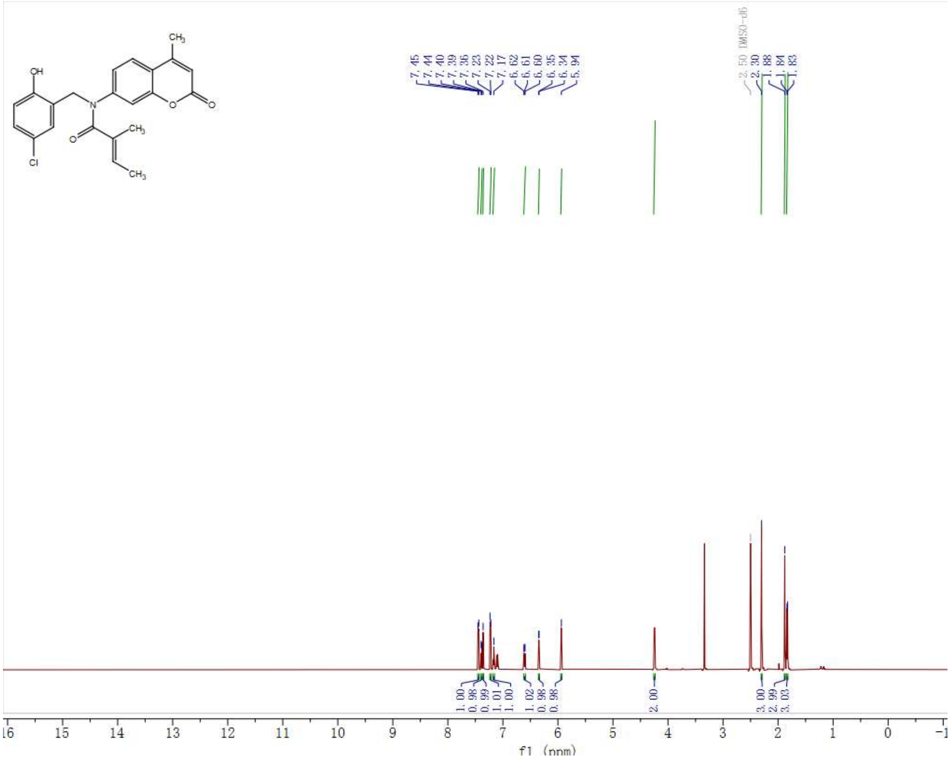

^{13}C NMR of compound 4t.

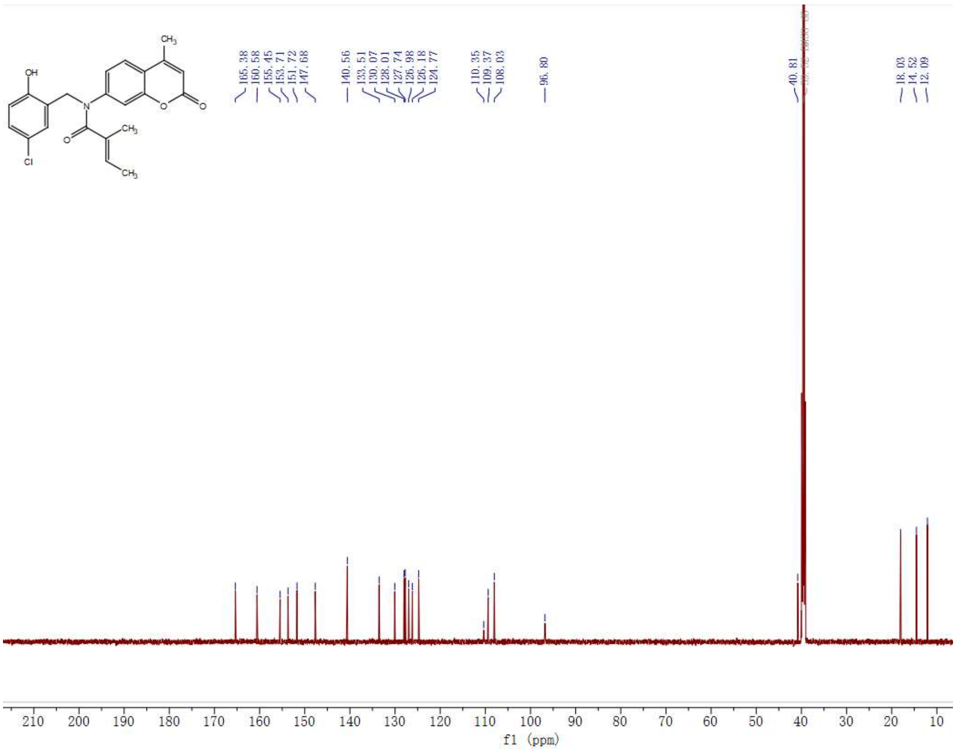

Selectivity screening against HDAC1, HDAC2, and HDAC6:

To clarify the selectivity of the synthesized compounds for HDAC isoforms, compound 4s was selected as a representative molecule and docked against HDAC1 (PDB ID: 5ICN), HDAC2 (PDB ID: 4LXZ), and HDAC6 (PDB ID: 5EDU) under identical conditions. The optimal docking scores are summarized in Table 1. For HDAC1 and HDAC2, the docking scores (S values, kcal/mol) of compound 4s were close to or exceeded those of the positive control SAHA.

The docking scores of compound 4s against HDAC1, HDAC2, and HDAC6 were –5.64, –5.60, and –5.86 kcal/mol, respectively. Despite the similar scores, binding mode analysis revealed that 4s adopted a typical inhibitory conformation with zinc-coordinating residues and the hydrophobic channel in HDAC1 and HDAC2 (Figure 1A/C, Figure 2A/E). In contrast, while 4s was able to enter the active site of HDAC6, it failed to effectively engage the catalytic core residues and was affected by steric hindrance from Phe620 (Figure 2C/G). SAHA exhibited equal or higher scores and more extensive interactions across all isoforms. These results suggest that 4s may act as a class I HDAC-selective inhibitor. In summary, the molecular docking results indicate that compound 4s may possess selective inhibitory activity against class I HDACs (HDAC1/2) while exhibiting weaker inhibition against HDAC6.

Table S1. Molecular docking scores of compound 4s with three HDAC proteins (HDAC1, HDAC2, and HDAC6).

Target	PDB ID	The best docking score of 4s	The best docking score of SAHA
HDAC1	5ICN	-5.6438	-5.9836
HDAC2	4LXZ	-5.5998	-5.5796
HDAC6	5EDU	-5.8643	-7.0859

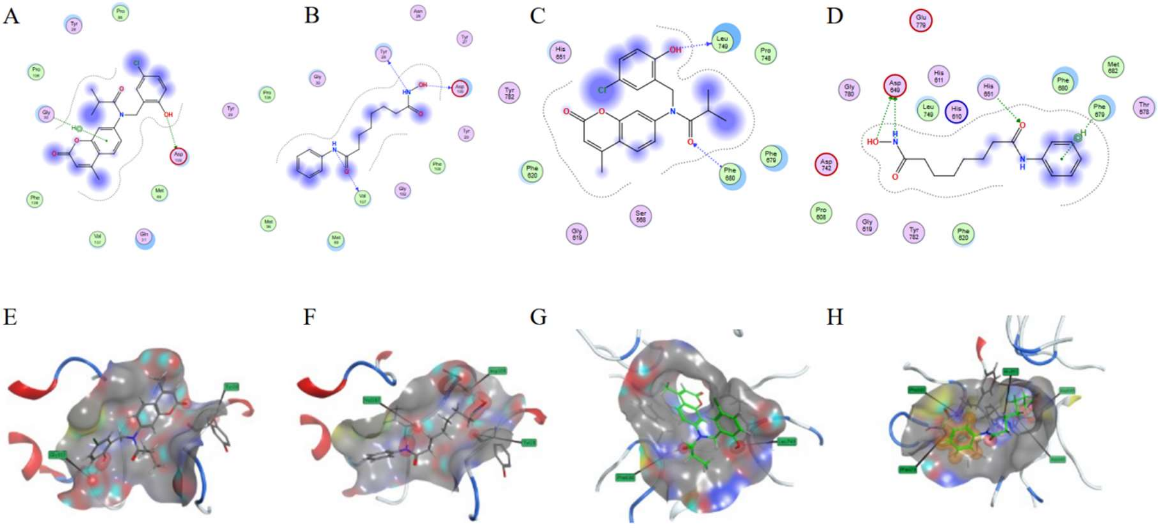

Figure.S1 Molecular docking of compound 4s and SAHA with HDAC2 and HDAC6 (PDB: 4LXZ and 5EDU). (A) 2D diagram of 4s-4LXZ; (B) 2D diagram of SAHA-4LXZ; (C) 2D diagram of 4s-5EDU; (D) 2D diagram of SAHA-5EDU; (E) 3D diagram of 4s-4LXZ; (F) 3D diagram of SAHA-4LXZ; (G) 3D diagram of 4s-5EDU; (H) 3D diagram of SAHA-5EDU.
